# Introduction of the *Exocelina ekari*-group with descriptions of 22 new species from New Guinea (Coleoptera, Dytiscidae, Copelatinae)

**DOI:** 10.3897/zookeys.250.3715

**Published:** 2012-12-13

**Authors:** Helena V. Shaverdo, Suriani Surbakti, Lars Hendrich, Michael Balke

**Affiliations:** 1 Naturhistorisches Museum, Burgring 7, A-1010 Vienna, Austria; 2 Department of Biology, Universitas Cendrawasih, Jayapura, Papua, Indonesia; 3 Zoologische Staatssammlung München, Münchhausenstraße 21, D-81247 Munich, Germany; 4 Zoologische Staatssammlung München, Münchhausenstraße 21, D-81247 Munich, Germany and GeoBioCenter, Ludwig-Maximilians-University, Munich, Germany

**Keywords:** *Exocelina ekari*-group, Copelatinae, Dytiscidae, new species, lectotype designation, New Guinea

## Abstract

The *Exocelina ekari*-group is here introduced and defined mainly on the basis of a discontinuous outline of the median lobe of the aedeagus. The group is known only from New Guinea (Indonesia and Papua New Guinea). It contained four species to date: *Exocelina astrophallus* (Balke, 1998), *Exocelina atowaso* (Shaverdo, Sagata & Balke, 2005), *Exocelina munaso* (Shaverdo, Sagata & Balke, 2005), and *Exocelina polita* (Sharp, 1882). Twenty two new species are described herein: *Exocelina alexanderi* **sp. n.**, *Exocelina anggiensis* **sp. n.**, *Exocelina arfakensis* **sp. n.**, *Exocelina bifida* **sp. n.**, *Exocelina brahminensis* **sp. n.**, *Exocelina bundiensis* **sp. n.**, *Exocelina edeltraudae* **sp. n.**, *Exocelina ekari* **sp. n.**, *Exocelina eme* **sp. n.**, *Exocelina evelyncheesmanae* **sp. n.**, *Exocelina hansferyi* **sp. n.**, *Exocelina irianensis* **sp. n.**, *Exocelina kakapupu* **sp. n.**, *Exocelina knoepfchen* **sp. n.**, *Exocelina oceai* **sp. n.**, *Exocelina pseudosoppi* **sp. n.**, *Exocelina soppi* **sp. n.**, *Exocelina unipo* **sp. n.**, *Exocelina utowaensis* **sp. n.**, *Exocelina waigeoensis* **sp. n.**, *Exocelina weylandensis* **sp. n.**, and *Exocelina wondiwoiensis* **sp. n.** The lectotype of *Copelatus politus* Sharp, 1882 is designated. A checklist and identification key to all species of the group are provided and important diagnostic characters (habitus, color, male antennae and protarsomeres 4–5, median lobes and parameres) are illustrated. Data on the distribution and habitat requirements are given. Representatives of the *Exocelina ekari*-group are so far mostly known from lowland to lower montane habitats of the northern and central parts of New Guinea, the group is less diverse in higher altitudes.

## Introduction

*Exocelina* Broun, 1886 is the second largest genus of the subfamily Copelatinae and is distributed in the Oriental, Australian, and Pacific Regions. At present, it includes 94 valid species. Fifteen species occur in Australia and New Zealand (Balke et al. 2004a, 2004b, 2007, Nilsson and Fery 2006, Nilsson 2007, Watts and Humphreys 2009), 36 species in New Caledonia (Wewalka et al. 2010), one species in China (Balke and Bergsten 2003), and one species in Hawaii (Nilsson and Fery 2006, Nilsson 2007). New Guinea is the core of the biodiversity of the genus (Balke 1998, 1999, Shaverdo et al. 2005). Phylogenetic analyses, based on molecular data, substantiate New Guinean *Exocelina* as a monophyletic group, which emerged from a single colonization event by an Australian species that led to a radiation of very high number of New Guinean species (Balke et al. 2004a, 2007). At present, 41 species are described from the island, which is only a small part of its real extraordinary rich *Exocelina* fauna. Extensive fieldwork and taxonomic investigation revealed the existence of more than 90 additional new species which are under study now.

Especially the New Guinean *Exocelina* species are superficially very similar to each other, and only closer inspection reveals stunning morphological diversity. It is difficult to build a species-group structure of the genus. Therefore, previous studies defined only four species groups which included 17 species: the *Exocelina ullrichi*-group, *Exocelina rivulus*-group (Balke 1998), *Exocelina broschii*-group (Shaverdo et al. 2005), and *Exocelina* *aipo*(*me*)-group (Balke 1998, Balke et al. 2007). Our examination of recently obtained material of New Guinean *Exocelina* showed that, based on morphology of male genitalia, many species can be organized into the *Exocelina ekari*-group. It was first mentioned by Balke et al. (2007: 511–512) for three described species: *Exocelina astrophallus* (Balke, 1998), *Exocelina atowaso* (Shaverdo, Sagata & Balke, 2005), and *Exocelina munaso* (Shaverdo, Sagata & Balke, 2005) and five undescribed species. Phylogenetic analysis of these species, based on DNA sequence data, showed that they form a monophyletic group (Balke et al. 2007). During our study, one more of the described species, *Exocelina polita* (Sharp, 1882), was placed into the *Exocelina ekari*-group. From the recently studied material, more than 40 new species also were found to belong to this group. Twenty two of them are described in the present paper and the remaining species will be treated in further publications. The present work is also aimed to define the *Exocelina ekari*-group, to provide an identification key to its described species, as well as information about their distribution and habitats.

We fully embrace new technology: wiki-engine powered species pages were automatically created by ZooKeys with the publication of this article. These species pages are versioned and can and will be enriched with further information from time to time, such as habitat photographs, videos, and links to public repositories of DNA sequence data which we currently analyse. Our species pages are on species-id.net portal.

## Material and methods

The present work is based on the material from the following collections:

**ANIC** Australian National Insect Collection, CSIRO Entomology, Canberra, Australia (Dr T.A. Weir)

**BMNH** The Natural History Museum, London, UK (Mrs C. Taylor)

**CASk** collection of Andre Skale, Hof/Saale, Germany

**CLH** collection of Lars Hendrich, Munich, Germany (property of NHMW)

**MZB** Museum Zoologicum Bogoriense, Cibinong, Indonesia (Mrs P. Lupiyaningdyah)

**NARI** Papua New Guinea National Insect Collection, Port Moresby, PNG (Mr Mark Ero)

**NHMW** Naturhistorisches Museum Wien, Vienna, Austria (Dr M.A. Jäch)

**SMNS **Staatliches Museum für Naturkunde Stuttgart (Dr W. Schawaller)

**ZSM** Zoologische Staatsammlung München, Munich, Germany (Dr M. Balke)

All specimen data are quoted as they appear on the labels attached to the specimens. Label text is cited using quotation marks. Comments in square brackets are ours. We extracted DNA and obtained DNA sequence data for some of the species/specimens, marked with individual DNA extraction numbers (e.g. “256 DNA M. Balke”). All types of the herein described specimens are provided with red labels. The female specimens, identification of which is difficult or sometimes impossible, are included in the type series only when collected with males of respective species and do not show external morphological differences from them. If two or more morphologically similar species collected together (males found together), their females were not included in the types series of the respective species but were mentioned under additional material.

Measurements were taken with a Wild M10 stereomicroscope. The following abbreviations were used: TL (total body length), TL-H (total body length without head), MW (maximum body width), and hw (handwritten). Number of the ventral setae of the male protarsomere 5 is given only for one specimen of each species, which was mounted on a glass slide (see below) for drawing. This character was found not very useful for the species identification since it is possible to make a general statement of the setation pattern (short/long, dense/sparse) but not to count them with certainty at the magnification of normal dissecting scopes. The potential phylogenetic information content of this character will be studied in a further work.

Drawings were made with the aid of a camera lucida attached to a Leica DM 2500 microscope. For detailed study and drawing, antennae, protarsi, and genitalia were removed and mounted on glass slides with DMHF (dimethyl hydantoin formaldehyde) as temporary preparations. The drawings were scanned and edited, using the software Adobe Illustrator CS5.1. Arrangement of the figures follows the species order in the key.

The terminology to denote the orientation of the genitalia (ventral for median lobe and dorsal and external for paramere) follows Miller and Nilsson (2003). The term “sternite 7” is used to indicate the last abdominal sternite (ventrite). Administrative divisions of Indonesia and Papua New Guinea follow information from Wikipedia (2012).

## Diagnosis of the *Exocelina ekari*-group

This group is newly introduced herein, and the following diagnostic characters of the group are proposed:

- beetles small or middle-sized (TL-H 3.0–5.2 mm);

- habitus oblong-oval (broadest approximately at elytral middle), with rounded pronotal and elytral sides, body outline continuous;

- pronotum short, trapezoidal, with posterior angles not drawn backwards;

- coloration red, reddish-brown to piceous, mainly uniform, sometimes with paler head and pronotum and darker elytra;

- microreticulation and punctation of dorsal suface very fine to strongly impressed, so that beetles shiny to matt dorsally;

- metacoxae and abdominal sternites 2–6 (and 7 in males) with thin, almost longitudinal striae/strioles;

- pronotum and elytra without striae or strioles;

- pronotum with or without lateral bead;

- male antennomeres simple or antennomeres 3–10 differently modified;

- male protarsomeres 1–3 not expanded laterally;

- male protarsomere 4 cylindrical, narrow, with small to large anterolateral hook;

- male protarsomere 5 not modified: long and narrow, without expansion and concavity, ventrally with two sparse rows of relatively short setae;

- median lobe of aedeagus with discontinuous outline in ventral view and sometimes in lateral view;

- ventral sclerite of median lobe more or less deeply divided on the middle, in some species like two long subequal ventral sclerites, because of strong sclerotisation of its lateral sides;

- apical part of median lobe with or without setae;

- paramere with or without notch on dorsal side;

- paramere with long setae occupying whole dorsal side, in most species these setae denser and stronger on subdistal part of paramere and can be of different length and shape.

**Figure 1.**
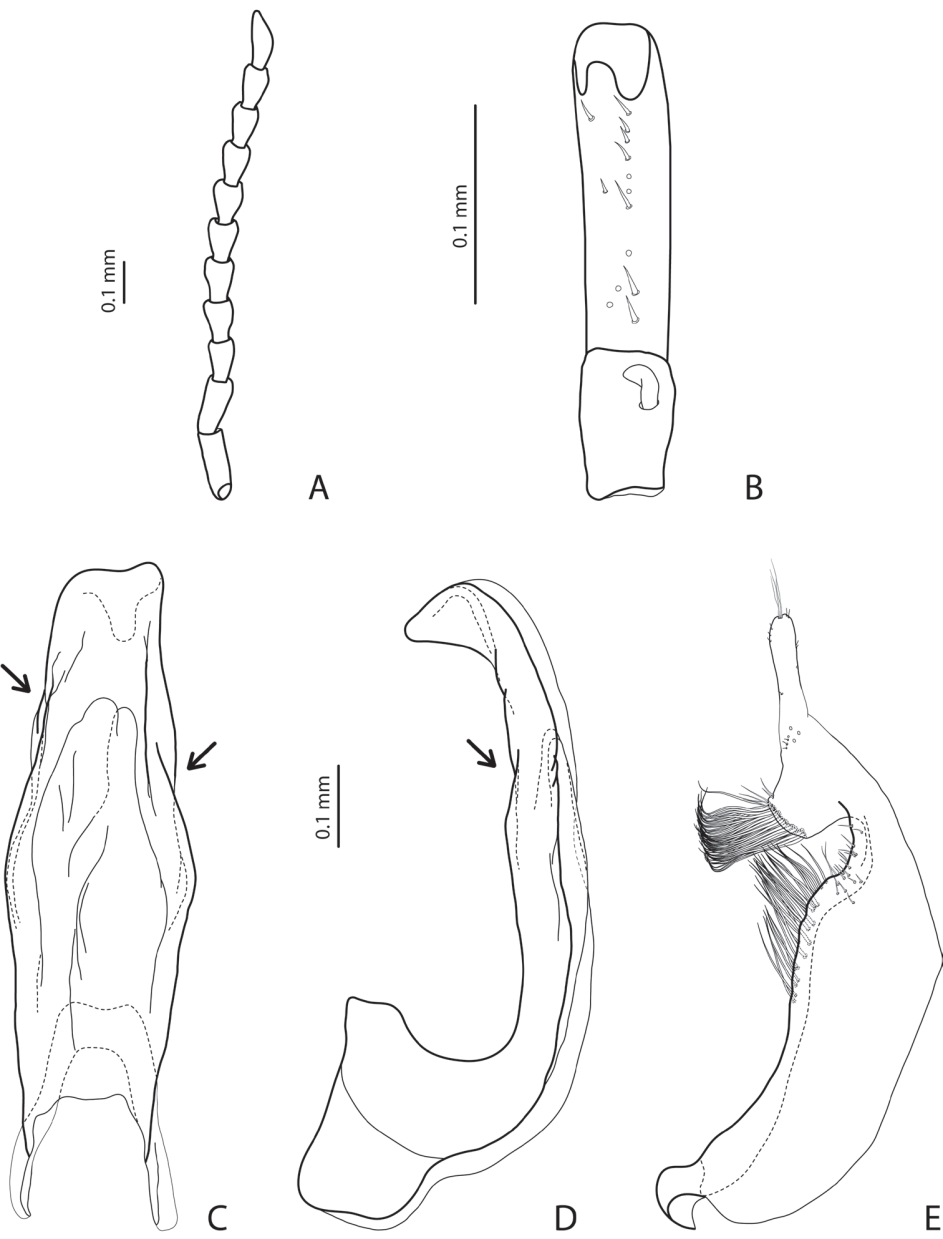
*Exocelina oceai* sp. n. **A** male antenna **B** protarsomeres 4–5 in ventral view **C** median lobe in ventral view **D** median lobe in lateral view **E** paramere in external view.

**Figures 2–3.**
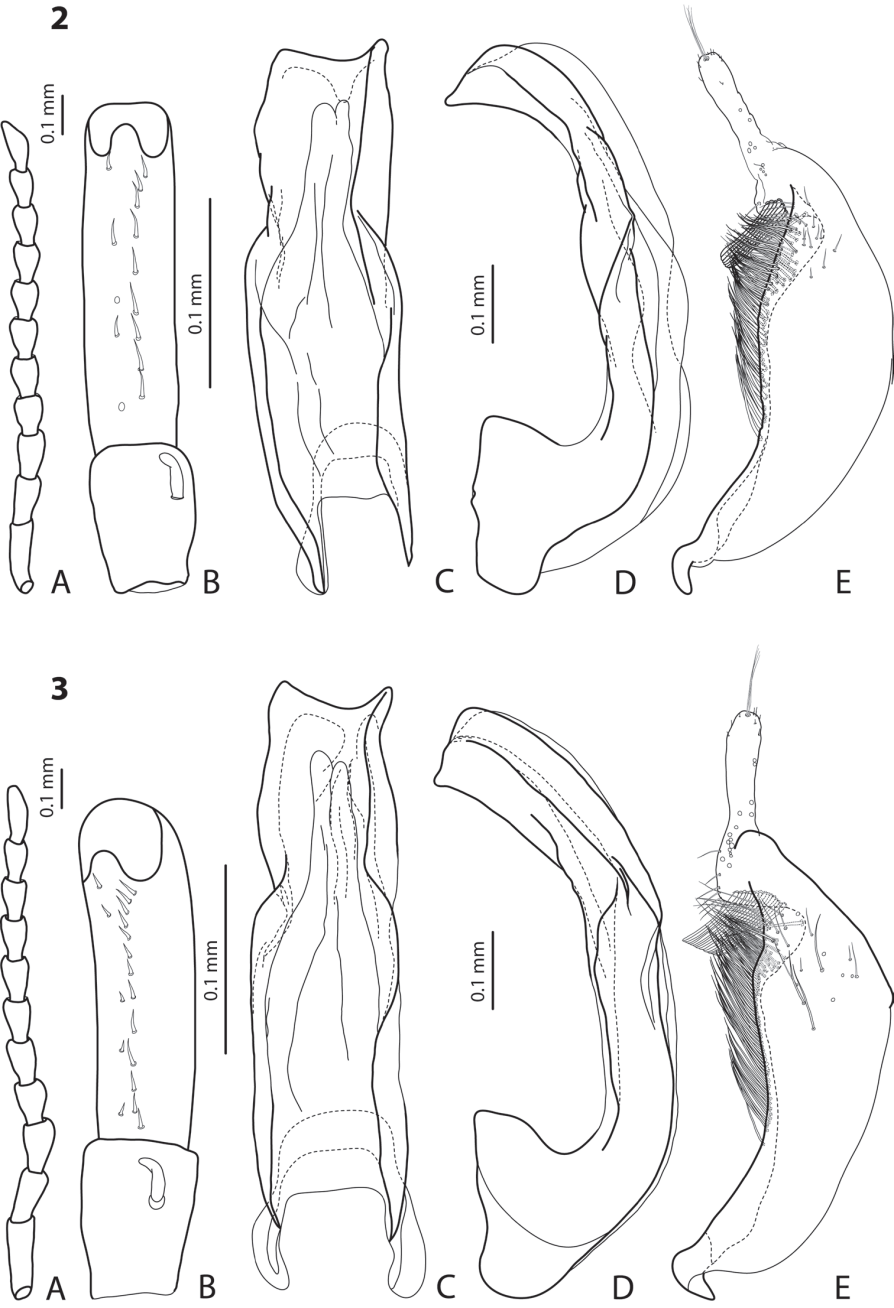
**2** *Exocelina waigeoensis* sp. n. **3** *Exocelina evelyncheesmanae* sp. n. **A** male antenna **B** protarsomeres 4–5 in ventral view **C** median lobe in ventral view **D** median lobe in lateral view **E** paramere in external view.

**Figure 4.**
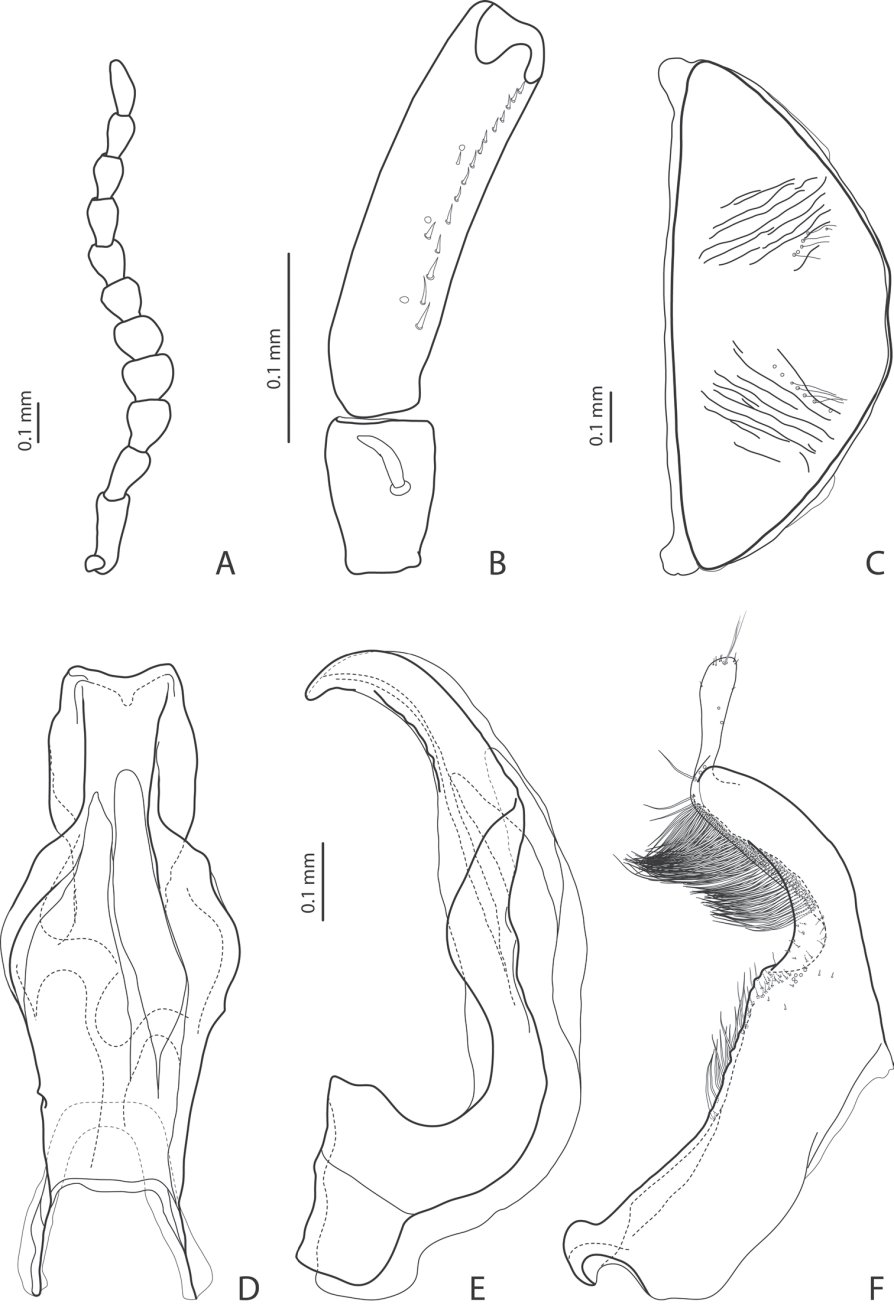
*Exocelina edeltraudae* sp. n. **A** male antenna **B** protarsomeres 4–5 in ventral view **C** abdominal sternite 7 **D** median lobe in ventral view **E** median lobe in lateral view **F** paramere in external view.

The main diagnostic character of the group is discontinuous outline of the median lobe of the aedeagus in ventral view and sometimes in lateral view (showed with arrows in Figs 1C, D). The discontinuous outline of the median lobe is present due to curved, plicate, or corrugated lateral sides of the lobe. It can be differently developed, for example very strongly, forming submedian constriction, as e.g. in Figs 2C, 3C, and 4D, or rather weakly as e.g. in Figs 7C, 14D, and 17C.

### Checklist and distribution of the species of the *Exocelina ekari*-group

**Table:** Abbreviations: IN – Indonesia, PNG – Papua New Guinea.

1. *Exocelina alexanderi* sp. n.	IN: West Papua: Manokwari
2. *Exocelina anggiensis* sp. n.	IN: West Papua: Manokwari
3. *Exocelina arfakensis* sp. n.	IN: West Papua: Manokwari
4. *Exocelina astrophallus* (Balke, 1998)	PNG: Madang
5. *Exocelina atowaso* (Shaverdo, Sagata & Balke, 2005)	PNG: Madang, East Sepik, Enga
6. *Exocelina bifida* sp. n.	IN: Papua: Jayawijaya
7. *Exocelina brahminensis* sp. n.	PNG: Sandaun, East Sepik, Madang, Morobe
8. *Exocelina bundiensis* sp. n.	PNG: Madang, Eastern Highlands
9. *Exocelina edeltraudae* sp. n.	PNG: Western and Southern Highlands
10. *Exocelina ekari* sp. n.	IN: Papua: Nabire, Paniai
11. *Exocelina eme* sp. n.	IN: Papua: Jayawijaya
12. *Exocelina evelyncheesmanae* sp. n.	IN: West Papua: Raja Ampat
13. *Exocelina hansferyi* sp. n.	PNG: Morobe
14. *Exocelina irianensis* sp. n.	IN: Papua: Nabire, Paniai
15. *Exocelina kakapupu* sp. n.	IN: Papua: Nabire, Paniai
16. *Exocelina knoepfchen* sp. n.	PNG: Eastern Highlands
17. *Exocelina munaso* (Shaverdo, Sagata & Balke, 2005)	PNG: Simbu, Eastern Highlands
18. *Exocelina oceai* sp. n.	IN: Papua: Nabire, Paniai
19. *Exocelina polita* (Sharp, 1882)	IN: West Papua: Manokwari
20. *Exocelina pseudosoppi* sp. n.	IN: Papua: Nabire, Paniai
21. *Exocelina soppi* sp. n.	IN: Papua: Nabire, Paniai
22. *Exocelina unipo* sp. n.	IN: Papua: Nabire, Paniai
23. *Exocelina utowaensis* sp. n.	IN: Papua: Nabire, Paniai
24. *Exocelina waigeoensis* sp. n.	IN: West Papua: Raja Ampat
25. *Exocelina weylandensis* sp. n.	IN: Papua: Nabire, Paniai
26. *Exocelina wondiwoiensis* sp. n.	IN: West Papua: Teluk Wondama

## Species descriptions

### 1. Exocelina alexanderi

Shaverdo, Hendrich & Balke sp. n.

urn:lsid:zoobank.org:act:73F610DB-B03A-4313-B00B-71F61E40FD69

http://species-id.net/wiki/Exocelina_alexanderi

Figs 8A-E , 34

#### Type locality.

Indonesia: West Papua Province: Manokwari Regency, Arfak Mts., Tetaho area, Iranmeba.

#### Type material.

*Holotype*: male “IRIAN JAYA: Vogelkop Tetaho area, Iranmeba 1500-1700 m, 25.3.1993 leg. A. Riedel” (NHMW). *Paratypes*: 8 males, 6 females with the same label as the holotype, 1 male additionally with a green label “DNA M.Balke 3255” (NHMW, ZSM). 1 male “IRIAN JAYA: Vogelkop Testega-Meydoudga 1100 m, 4.4.1993 leg. A. Riedel” (NHMW). 1 male “Testegal / Iranmeba” [hw] (ZSM).

#### Diagnosis.

Beetle middle-sized, dark brown to piceous; pronotum with distinct lateral bead; male antennomeres 3 and 4 evidently larger than other, with external margin expanded (antennomeres triangular, elongated); male protarsomere 4 with small (only slightly larger than more laterally situated large seta), thin, slightly curved anterolateral hook; median lobe with strong submedian constriction in ventral view, apex of median lobe almost truncate in lateral view and broad in ventral view; paramere with notch on dorsal side and subdistal part short and small, with not numerous, relatively short, thick, and flattened setae. The species is well recognizable by the modified antennae of the males.

#### Description.

*Size and shape*: Beetle middle-sized (TL-H 3.9–4.05 mm, TL 4.35–4.5 mm, MW 2.1–2.2 mm), with oblong-oval habitus, broadest at elytral middle. *Coloration*: Dorsally dark brown to piceous, with paler (reddish) anterior margin and vertex of head, lateral sides of pronotum, and narrow bands along elytral suture; head appendages yellowish to reddish-brown, legs slightly darker (Fig. 34).

*Surface sculpture*: Head with dense punctation (spaces between punctures 1–3 times size of punctures), evidently finer and sparser anteriorly; diameter of punctures smaller than diameter of cells of microreticulation. Pronotum with finer, sparser, and more evenly distributed punctation than on head. Elytra with very sparse and fine punctation, almost invisible. Head, pronotum, and elytra with strongly impressed microreticulation, dorsal surface shiny but duller than of *Exocelina oceai* sp. n. Head with microreticulation stronger. Metaventrite and metacoxa distinctly microreticulate, metacoxal plates with longitudinal strioles and transverse wrinkles. Abdominal sternites with distinct microreticulation, strioles, and fine sparse punctation, coarser and denser on two last abdominal sternites.

*Structures*: Pronotum with distinct lateral bead. Base of prosternum and neck of prosternal process with distinct ridge, without anterolateral extensions. Blade of prosternal process lanceolate, narrow, convex, with distinct bead and few setae; neck and blade of prosternal process evenly jointed. Abdominal sternite 7 broadly rounded apically.

*Male*: Antennomeres 3–6 strongly enlarged, antennomeres 3 and 4 evidently larger than other, with external margin expanded (antennomeres triangular, elongated), 7–9 somewhat enlarged (Fig. 8A); antennomeres 3–7 rugose ventrally. Protarsomere 4 with small, thin, slightly curved anterolateral hook. Protarsomere 5 ventrally with anterior row of 14 short setae and posterior row of 7 short setae (Fig. 8B). Abdominal sternite 7 with 10–15 lateral striae on each side. Median lobe with strong submedian constriction in ventral view, apex of median lobe almost truncate in lateral view and broad in ventral view (Figs 8C, D). Paramere with notch on dorsal side and subdistal part short and small, with not numerous, relatively short, thick, and flattened setae (Fig. 8E).

*Female*: Antennae simple, abdominal sternite 7 without striae.

#### Distribution.

Indonesia: West Papua Province: Manokwari Regency. This species is known from the eastern Bird’s head only. Iranmeba and Testega are situated some 20–30 km west of Anggi-Lakes in the Arfak Mountains (Fig. 50).

#### Etymology.

The species is named for friend and most enthusiastic explorer of New Guinea’s entomofauna, Alexander Riedel (Karlsruhe, Germany), who discovered this species. The species name is a noun in the genitive case.

### 2. Exocelina anggiensis

Shaverdo, Hendrich & Balke sp. n.

urn:lsid:zoobank.org:act:10D09225-4873-499F-9BA2-0A870DF117A8

http://species-id.net/wiki/Exocelina_anggiensis

Figs 9A–E , 35

#### Type locality.

Indonesia: West Papua Province: Manokwari Regency, Arfak Mts., Anggi, Iray, 01°20.51'S, 133°55.64'E.

#### Type material.

*Holotype*: male “Indonesia: Papua, Anggi, forest above Iray 1, 2000m, 2.ii.1994, 01.20.512S, 133.55.642E, Balke (BH 06)” (ZSM). *Paratypes*: 5 males, 8 females with same labels as the holotype, one of them additionally with a green label “DNA M.Balke 1272” (NHMW, ZSM). 1 female “Indonesia: Papua, Anggi, Iray, 1880m, 1.ii.1994, 01.18.224S, 133.54.009E, Balke (BH 05)”, “DNA M.Balke 1271” [green] (ZSM).

#### Diagnosis.

Beetle middle-sized, reddish-brown to brown; submatt; pronotum with lateral bead; male antennomeres 3–4 strongly enlarged and triangular (3 larger than 4), 5–6 distinctly enlarged, 7 somewhat enlarged; male protarsomere 4 with small (only slightly larger than more laterally situated large seta), thin, slightly curved anterolateral hook; median lobe with strong submedian constriction in ventral view, apex of median lobe elongate in lateral view and narrower in ventral view; paramere with notch on dorsal side and subdistal part short and small, with not numerous, relatively short, thick, and flattened setae.

#### Description.

*Size and shape*: Beetle middle-sized (TL-H 3.9–4.3 mm, TL 4.25–4.7 mm, MW 2.05–2.3 mm), with oblong-oval habitus, broadest at elytral middle. *Coloration*: Head red to reddish-brown, with brown to dark brown areas behind eyes and “V”-like spot in middle, pronotum red to reddish-brown, with two darker areas laterally on disc (left and right from middle line), in some specimens jointed in middle, elytra reddish brown to dark brown, with reddish lines along sutura, head appendages yellowish red to red, legs red to reddis-brown, slightly darker distally; two paratypes with more uniform brown coloration (Fig. 35).

*Surface sculpture*: Whole dorsal surface with dense and coarse punctation (spaces between punctures 1–3, often 1–2, times size of punctures). Punctation slightly sparser on elytra (spaces between punctures 1–4 times size of punctures). Pronotum and elytra with strongly impressed microreticulation, dorsal surface, thus, submatt. Head with microreticulation stronger. Metaventrite and metacoxa distinctly microreticulate, metacoxal plates with longitudinal strioles and transverse wrinkles. Abdominal sternites with distinct microreticulation, strioles, and fine, rather dense punctation, coarser and denser on two last abdominal sternites.

*Structures*: Pronotum with distinct lateral bead, sides somewhat more rounded. Base of prosternum and neck of prosternal process with distinct ridge, not smooth and slightly rounded anteriorly, without anterolateral extensions. Blade of prosternal process lanceolate, relatively narrow, slightly convex, with distinct bead and few setae; neck and blade of prosternal process evenly jointed. Abdominal sternite 7 broadly rounded apically.

*Male*: Antennomeres 3–4 strongly enlarged and triangular (3 larger than 4), 5–6 distinctly enlarged, 7 somewhat enlarged (Fig. 9A); antennomeres 3–6 rugose ventrally. Protarsomere 4 with small (only slightly larger than more laterally situated large seta), thin, slightly curved anterolateral hook. Protarsomere 5 ventrally with anterior row of 9 short setae and posterior row of 7 short setae (Fig. 9B). Abdominal sternite 7 with 8–10 lateral striae on each side. Median lobe with strong submedian constriction in ventral view, apex of median lobe elongate in lateral view and narrower in ventral view (Figs 9C, D). Paramere with notch on dorsal side and subdistal part short and small, with not numerous, relatively short, thick, and flattened setae (Fig. 9E).

*Female*: Dorsal punctation denser and coarser than in males, antennae simple, abdominal sternite 7 without striae.

#### Distribution.

Indonesia: West Papua Province: Manokwari Regency. The species is known only from the type locality (Fig. 50).

#### Etymology.

The species is named for the type area, Anggi. The name is an adjective in the nominative singular.

### 3. Exocelina arfakensis

Shaverdo, Hendrich & Balke sp. n.

urn:lsid:zoobank.org:act:0DA24F49-2FC3-4A96-9C40-609CCC9B7C69

http://species-id.net/wiki/Exocelina_arfakensis

Figs 10A–E , 36

#### Type locality.

Indonesia: West Papua Province: Manokwari Regency, Arfak Mts., Sijoubreg Village near Mokwam, 01°06.56'S, 133°54.61'E.

#### Type material.

*Holotype*: male “Indonesia: Papua, Sijoubreg Vill. nr Mokwam, 1450-1600m, 26.i.1994, 01.06.561S, 133.54.606E, Balke (BH 02)” (ZSM). *Paratypes*: 22 males, 18 females with the same labels as the holotype, one male additionally with a green label “DNA M.Balke 1268” (NHMW, ZSM). 8 males, 5 females “Indonesia: Papua, Manokwari-Minyambou, 1630m, 6.ii.1994, 01.06.115S, 133.53.261E, Balke (BH 08)”, one male additionally with a green label “DNA M.Balke 1273” (NHMW, ZSM). 3 males, 3 females “W-PAPUA Manokwari Prov. vic. Mokwam (Siyoubrig), 1400-1800m, 01°06.26'S, 133°54.41'E 24.-28.II.2007 leg. A. Skale” (CASk). 1 female “W-PAPUA Manokwari Prov. Mokwam, 1400-1800m S 01°06"43'S, 133°54"68'E 24.-28.II.2007 leg. A. Skale” (CASk).

#### Diagnosis.

Beetle middle-sized, piceous, shiny but with evident dorsal punctation; pronotum with distinct lateral bead; male antennomeres 3–4 strongly enlarged and triangular (3 larger than 4), 5–6 distinctly enlarged, 7 somewhat enlarged; male protarsomere 4 with small, thick (evidently thicker and slightly longer than more laterally situated large seta), slightly curved anterolateral hook; median lobe with strong submedian constriction in ventral view, apex of median lobe elongate in lateral view and narrower in ventral view; paramere with notch on dorsal side and subdistal part short and small, with not numerous, relatively short, thick, and flattened setae.

#### Description.

*Size and shape*: Beetle middle-sized (TL-H 3.7–4.1 mm, TL 4.1–4.5 mm, MW 2.05–2.2 mm), with oblong-oval habitus, broadest at elytral middle. *Coloration*: Dorsally piceous, with dark brown pronotal sides; head appendages yellow to red, legs darker (Fig. 36).

*Surface sculpture*: Head with very dense, coarse punctation (spaces between punctures 1–2 times size of punctures), especially on vertex. Pronotum and elytra with punctation much finer and sparser. Pronotum and elytra with somewhat stronger impressed microreticulation, but dorsal surface still shiny. Head with microreticulation stronger. Metaventrite and metacoxa distinctly microreticulate, metacoxal plates with longitudinal strioles and transverse wrinkles. Abdominal sternites with distinct microreticulation, strioles, and fine sparse punctation, coarser and denser on two last abdominal sternites.

*Structures*: Pronotum with distinct lateral bead. Base of prosternum and neck of prosternal process with distinct ridge, rounded and smooth anteriorly, with small anterolateral extensions. Blade of prosternal process lanceolate, relatively broad, convex, with distinct bead and few setae; neck and blade of prosternal process evenly jointed. Abdominal sternite 7 broadly rounded apically.

*Male*: Antennomeres 3–4 strongly enlarged and triangular (3 larger than 4), 5–6 distinctly enlarged, 7 somewhat enlarged (Fig. 10A); antennomeres 3–7 rugose ventrally. Protarsomere 4 with small, thick (evidently thicker and slightly longer than more laterally situated large seta), slightly curved anterolateral hook. Protarsomere 5 ventrally with anterior row of 11 short setae and posterior row 5 short setae (Fig. 10B). Abdominal sternite 7 with 6–8 lateral striae on each side. Median lobe with strong submedian constriction in ventral view, apex of median lobe elongate in lateral view and narrower in ventral view (Figs 10C, D). Paramere with notch on dorsal side and subdistal part short and small, with not numerous, relatively short, thick, and flattened setae (Fig. 10E).

*Female*: Dorsal punctation slightly coarser, antennae simple, sternite 7 without striae.

#### Distribution.

Indonesia: West Papua Province: Manokwari Regency. The species is known only from the Arfak Mountains, the eastern part of Bird’s head (Fig. 50).

#### Etymology.

The species is named for the type area, Arfak Mountains. The name is an adjective in the nominative singular.

### 4. Exocelina astrophallus

(Balke, 1998)

http://species-id.net/wiki/Exocelina_astrophallus

Fig. 27

Copelatus (Papuadytes) astrophallus Balke, 1998: 324.

#### Type locality.

Papua New Guinea: Madang Province, 1–3 km SE Brahman.

#### Type material examined.

*Paratypes*: 5 males “PAPUA NEW GUINEA Madang, 1-3 km SE Brahman, June 21 1991. D. Larson” (NHMW).

#### Additional material.

1 male “PAPUA NEW GUINEA Madang Pr. Brahmin, 150 m, 26IX2002, M Balke (PNG 24)”, “273 DNA M Balke” (ZSM). 6 males, 5 females, 7 exs. “Papua New Guinea: Madang, Usino, 260m, 15.iii.2007, 05.31.125S, 145.25.316E, Kinibel (PNG 158)”, one male additionally with a green label “DNA M. Balke 3320” (ZSM). 4 males “Papua New Guinea: Madang, Keki-Sewan, Adalbert Mts., 300m 30.xi.1994, 04.40.558S, 145.27.187E, Binatang Boys, (PNG 121)”, one of them additionally with a green label “DNA M. Balke 1529” (ZSM). 6 males, 8 females, 10 exs. “Papua New Guinea: Madang, Highway nr. Madang, ford, 80m, 26.xi./2.-3.xii.1994, 05.24.405S, 145.38.213E, Binatang Boys, (PNG 117)”, one of them additionally with a green label “DNA M. Balke 4090” (NHMW, ZSM). 6 males, 3 females “Papua New Guinea: Madang, nr. Brahmin, 200m, 25.xi.1994, nr. 05.47.026S, 145.24.131E, Balke & Kinibel (PNG 116)” (ZSM). 17 males, 11 females “Papua New Guinea: Madang, Wannang, 270m, 31.x.2008, 05.15.458S 145.02.389, Posman, (PNG187)”, one of them additionally with a green label “DNA M. Balke 4167” (NHMW, ZSM).

#### Diagnosis.

Beetle middle-sized, piceous, with paler pronotum (especially on margins) and head, teneral specimens reddish-brown, submatt; pronotum with distinct lateral bead; male antennomeres simple; male protarsomere 4 with middle-sized, thick, evidently curved anterolateral hook; median lobe short and with extremely strongly discontinuous (broken and curved) outline; paramere with shallow notch on dorsal side and subdistal part elongate, with dense, long, thin setae.

#### Additions and corrections to the description.

For the complete description see Balke (1998).

*Size and shape*: Beetle middle-sized (TL-H 3.8–3.9 mm, TL 4.05–4.2 mm, MW 2.0–2.15 mm), with oblong-oval habitus, broadest at elytral middle, with elytral apex slightly rounded. *Coloration*: piceous, with paler pronotum (especially on margins) and head, teneral specimens pale reddish brown to dark brown, with paler posterior part of head and lateral sides of pronotum (Fig. 27).

*Surface sculpture*: Head, pronotum, and elytra with distinct microreticulation and punctuation, dorsal surface, thus, submatt. Metaventrite and metacoxa distinctly microreticulate, metacoxal plates with longitudinal strioles and transverse wrinkles. Abdominal sternites with distinct microreticulation, strioles, and fine sparse punctation, coarser and denser on two last abdominal sternites.

*Structures*: Pronotum with distinct lateral bead. Base of prosternum and neck of prosternal process with sharp ridge, without anterolateral extensions. Blade of prosternal process lanceolate, rather narrow, strongly convex, with distinct bead and few setae; neck and blade of prosternal process evenly jointed.

*Male*: Antenna simple. Protarsomere 4 with middle-sized, thick, evidently curved anterolateral hook. Protarsomere 5 ventrally with anterior row of 36 longer setae and posterior row of 14 shorter setae. Median lobe short and with extremely strongly discontinuous (curved, plicate) outline. Paramere with shallow notch on dorsal side and subdistal part elongate, with dense, long, thin setae. See Figs 37, 46, 64 in Balke (1998).

*Female*: Without evident differences in external morphology from male, except for abdominal sternite 7 without striae.

#### Distribution.

Papua New Guinea. The species is known only from the Madang Province (Fig. 50).

### 5. Exocelina atowaso

(Shaverdo, Sagata & Balke, 2005)

http://species-id.net/wiki/Exocelina_atowaso

Fig. 25

Papuadytes atowaso Shaverdo, Sagata & Balke, 2005: 275.

#### Type locality.

Papua New Guinea: Madang Province, river below Bundi, approximately 05°44.85'S, 145°16.83'E.

#### Type material examined.

*Holotype*: male “PAPUA NEW GUINEA Madang Pr. below Bundi, 500 m, 26IX2002, M. Balke (PNG 23)”, “267 DNA M Balke” [green label] (BMNH). **Additional material. Madang:** 5 males, 4 females “Papua New Guinea: Madang, below Bundi, 500 m, 26.IX.2002 Balke & Sagata (PNG 023)” (NHMW, ZSM). **East Sepik/Enga:** 1 male “DNA M. Balke 4918”, “Papua New Guinea East Sepik, Lembena, 198m, 3.ix.2009, 04 46.974S 143 56.995E, Ibalim & Plus (PNG 243)” (ZSM). 1 ex. “Papua New Guinea East Sepik, Lembena, 198m, 3.ix.2009, 04 46.974S 143 56.995E, Ibalim & Plus (PNG 243)” (ZSM). 1 male “PNG 246” [hw] (ZSM). 1 male “Papua New Guinea East Sepik, Lembena, 335m, 10.ix.2009, 04 56.859S 143 59.375E, Ibalim & Plus (PNG 250)” (ZSM).

#### Diagnosis.

Beetle middle-sized, piceous, with paler head, shiny (Fig. 25); pronotum with distinct lateral bead; male antennomeres simple; male protarsomere 4 with middle-sized, slender, evidently curved anterolateral hook; median lobe with very strong submedian constriction and small subapical processes in ventral view and with almost rounded apex bearing setae in lateral view; paramere without notch on dorsal side, with relatively dense, long, thin setae.

#### Additions and corrections to the description.

For the complete description and illustrations see Shaverdo et al. (2005).

*Size*: Beetle middle-sized (TL-H 3.9–4.1 mm, TL 4.2–4.5 mm, MW 2.05–2.3 mm).

*Male*: Protarsomere 4 with middle-sized, slender, evidently curved anterolateral hook. Median lobe with very strong submedian constriction and small subapical processes in ventral view and with almost rounded apex bearing setae in lateral view. Paramere without notch on dorsal side, with relatively dense, long, thin setae. See Figs 9, 14a, b in Shaverdo et al. (2005).

*Female*: Without evident differences in external morphology from male, except for abdominal sternite 7 without striae.

#### Distribution and habitat.

Papua New Guinea. The species is known from Madang, East Sepik, and Enga Provinces (Fig. 50). For the habitat description see Shaverdo et al. (2005).

### 6. Exocelina bifida

Shaverdo, Hendrich & Balke sp. n.

urn:lsid:zoobank.org:act:C84D4E22-1C4F-447E-BADE-9353EE078FCF

http://species-id.net/wiki/Exocelina_bifida

Figs 15A–E , 41

#### Type locality.

Indonesia: Papua Province: Jayawijaya Regency, Borme, Tarmlu, approximately 04°24'S, 140°25'E.

#### Type material.

*Holotype*: male “IRIAN JAYA: Borme Tarmlu 1500m 6.9.1993”, “ca. 140°25'E, 04°24'S leg. M. Balke (4-6)” (NHMW). *Paratypes*: 6 males, 3 females with the same label as the holotype, one of the females additionally with a green label “DNA M.Balke 3256” (NHMW). 3 males “IRIAN JAYA: Borme Tarmlu 1500m 6.9.1993”, “ca. 140°25'E, 04°24'S leg. M. Balke (4)” (NHMW).

#### Diagnosis.

Beetle small, dark brown to piceous, shiny; pronotum without lateral bead; male antennomeres simple; male protarsomere 4 with large, thick, strongly curved anterolateral hook; median lobe with strong submedian constriction and apex bifid: with small dorsal extension; paramere with notch on dorsal side and subdistal part elongate, with dense, long, thin setae. The species is well recognizable by its characteristic male genitalia.

#### Description.

*Size and shape*: Beetle small (TL-H 3.5–3.75 mm, TL 3.9–4.25 mm, MW 1.8–2.0 mm), with oblong-oval habitus, broadest at elytral middle. *Coloration*: Head reddish-brown to piceous, paler anteriorly; pronotum dark brown to piceous, with paler sides, yellowish in anterior angles; elytra dark brown to piceous, with reddish-brown sutural bands; head appendages yellow to yellowish-red, legs distally darker (Fig. 41).

*Surface sculpture*: Head with dense punctation (spaces between punctures 1–3 times size of punctures), evidently finer and sparser anteriorly; diameter of punctures smaller than diameter of cells of microreticulation. Pronotum with much finer and sparser punctation than on head. Elytra with very sparse and fine punctation, almost invisible. Head, pronotum, and elytra with weakly impressed microreticulation, dorsal surface, thus, shiny. Head with microreticulation stronger. Metaventrite and metacoxa distinctly microreticulate, metacoxal plates with longitudinal strioles and transverse wrinkles. Abdominal sternites with distinct microreticulation, strioles, and fine sparse punctation, coarser and denser on two last abdominal sternites.

*Structures*: Pronotum without lateral bead. Base of prosternum and neck of prosternal process with distinct ridge, anteriorly less rounded, smooth, with small anterolateral extensions. Blade of prosternal process lanceolate, relatively broad, slightly convex, with distinct bead and few setae; neck and blade of prosternal process evenly jointed. Abdominal sternite 7 broadly rounded apically.

*Male*: Antenna simple (Fig. 15A). Protarsomere 4 with large, thick, strongly curved anterolateral hook. Protarsomere 5 ventrally with anterior row of 14 short setae and posterior row of 3 short setae (Fig. 15B). Abdominal sternite 7 with 6–10 lateral striae on each side. Median lobe with strong submedian constriction and apex bifid: with small dorsal extension (Figs 15C, D). Paramere with notch on dorsal side and subdistal part elongate, with dense, long, thin setae (Fig. 15E).

*Female*: Without evident differences in external morphology from male, except for abdominal sternite 7 without striae.

#### Distribution.

Indonesia: Papua Province, Jayawijaya Regency. This species is known only from the type locality (Fig. 50).

#### Etymology.

The name refers to the bifid distalodorsal part of the median lobe. The name is an adjective in the nominative singular.

### 7. Exocelina brahminensis

Shaverdo, Hendrich & Balke sp. n.

urn:lsid:zoobank.org:act:E31C6D32-6B87-4E19-B676-94F6EE79CA84

http://species-id.net/wiki/Exocelina_brahminensis

Figs 21A–E , 47

#### Type locality.

Papua New Guinea: Madang Province, Adalbert Mts., near Keki, 04°42.30'S, 145°25.09'E.

#### Type material.

*Holotype*: male “Papua New Guinea: Madang, Adalbert Mts., creek nr Keki, 790m, 28.xi.1994, 04.42.300S, 145.25.089E, Binatang Boys leg. (PNG 53a)” (ZSM). *Paratypes*: **Madang:** 20 males, 27 females with the same label as the holotype (NARI, NHMW, ZSM). 2 males “Papua New Guinea: Madang, Brahmin, 150 m, 26.IX.2002 Balke & Sagata (PNG 024)” (ZSM). 6 males, 1 female “Papua New Guinea: Madang, Akameku-Brahmin, Bismarck Range, 750m, 25.xi.1994, nr 05.49.307S, 145.24.389E, Balke & Kinibel (PNG 114)”, one of them additionally with a green label “DNA M.Balke 1524” (NHMW, ZSM). 20 males “Papua New Guinea: Madang, Usino, 260m, 15.iii.2007, 05.31.125S, 145.25.316E, Kinibel (PNG 158)”, one of them additionally with a green label “DNA M.Balke 3319” (ZSM). 1 male, 1 female “Papua New Guinea: Madang, Highway nr. Madang, ford, 80m, 26.xi./2.-3.xii.1994, 05.24.405S, 145.38.213E, Binatang Boys, (PNG 117)”, the male additionally with a green label: “DNA M.Balke 1523” (ZSM). 1 male, 2 female “Papua New Guinea: Madang, Mt. Tapo, 180 m, ii.2008 5 24.11.00 S 145 36 17 16 E, BRC leg. (PNG 178)” (ZSM). 2 males, 2 females “Papua New Guinea: Madang, Wannang, 230m 3.x.2008, 05.17.235S, 145.06.160E, Posman (PNG188)”, one of them additionally with a green label “DNA M.Balke 3760” (ZSM). 3 males, 6 females “Papua New Guinea: Madang, Wannang, 270m, 31.x.2008, 05.15.458S 145.02.389, Posman, (PNG187)”, one of them additionally with a green label: “DNA M. Balke 4168” (NHMW, ZSM). 29 males, 24 females “Papua New Guinea: Madang, Adalbert Mts., Sewan- Keki, 700m, 4.v.1994, 04.42.215S, 145.25.154E, Balke & Manaono (PNG 51)” (NHMW, ZSM). 14 males, 11 females “Papua New Guinea: Madang, Adalbert Mts., Keki, 850m, 5.v.1994, nr 04.42.300S, 145.25.089E, Balke & Manaono (PNG 52)” (NHMW, ZSM). 45 males, 29 females “Papua New Guinea: Madang, Adalbert Mts., below Keki, 790m, 5.v.1994, 04.42.300S, 145.25.089E, Balke & Manaono (PNG 53)” (NHMW, ZSM). 2 males, 6 females “Papua New Guinea: Madang, Adalbert Mts., Keki to Sewan, 650m, 7.v.1994, 04.41.802S, 145.25.460E, Balke (PNG 54)”, one of them additionally with a green label “DNA M.Balke 1298” (ZSM). 15 males, 16 females “Papua New Guinea: Madang, Keki, Adalbert Mts., 400m, 29.xi.1994, 04.43.058S, 145.24.437E, Binatang Boys, (PNG 119)” (NHMW, ZSM). 44 males, 35 females “Papua New Guinea: Madang, Keki-Sewan, Adalbert Mts., 700m, 30.xi.1994 nr 04.41.802S, 145.25.460E Binatang Boys (PNG 120)” (NHMW, ZSM). **Sandaun:** 2 males “Papua New Guinea: Sandaun, Toricelli Mts., village below Sibilanga Stn., 400m, 18.iv.1994, nr 03.26.750S, 142.29.949E, Balke (PNG 42)”, one of them additionally with a green label “DNA M.Balke 1293” (ZSM). 13 males, 8 females “Papua New Guinea: Sandaun, Toricelli Mts., 2h walk fr Sibilanga Stn., 350m, 19.iv.1994, 03.39.121S, 142.29.991E, Balke (PNG 44)” (NHMW, ZSM). **East Sepik:** 2 males, 2 females “Papua New Guinea: East Sepik, Prince Alexander Mts., Wewak, 400m, 21.iv.1994, 03.37.319S, 143.36.764E, Balke (PNG 45)”, one of them additionally with a green label “DNA M.Balke 1294” (NHMW, ZSM). **Morobe:** 1 male “Papua New Guinea: Morobe, Herzog Mts., Bundun, 700-800m, 2.iv.1994, 06.51.598S, 146.37.07E, Balke & Sagata (PNG 27)” (ZSM).

#### Additional material.

8 females “Papua New Guinea: Madang, Keki, Adalbert Mts., 400m, 29.xi.1994, 04.43.058S, 145.24.437E, Binatang Boys, (PNG 119)” (NHMW, ZSM). 8 females “Papua New Guinea: Madang, Keki-Sewan, Adalbert Mts., 700m, 30.xi.1994 nr 04.41.802S, 145.25.460E Binatang Boys (PNG 120)” (NHMW, ZSM). These females most likely belong to *Exocelina brahminensis* sp. n. However, the sure separation from *Exocelina larsoni* (Balke, 1998) is not possible because of more strongly developed traces of a lateral bead of the pronotum.

#### Diagnosis.

Beetle small, piceous, with reddish brown head and pronotum, shiny; pronotum without lateral bead or with weak traces of lateral bead; male antennomeres simple; male protarsomere 4 with large, thick, strongly curved anterolateral hook; median lobe with weak submedian constriction in ventral view; paramere with strong notch on dorsal side, with notch tip sharply pointed, and subdistal part elongate, with upper setae thin and more numerous and lower setae shorter, thick, and flattend. The species is similar to *Exocelina eme* sp. n., from which it differs by simple male antennae and the shape of the male genialia, especially by the strong notch of the paramere and its sharply poined tip.

#### Description.

*Size and shape*: Beetle small (TL-H 3.15–3.3 mm, TL 3.5–3.65 mm, MW 1.7–1.8 mm), with oblong-oval habitus, broadest at elytral middle. *Coloration*: Head reddish-brown, darker posterior eyes; pronotum reddish-brown, with slightly darker disc; elytra piceous, with dark brown sutural bands; head appendages yellow to yellowish-red, legs distally darker, to dark brown (Fig. 47).

*Surface sculpture*: as in *Exocelina eme* sp. n.

*Structures*: Pronotum without lateral bead or with weak traces of lateral bead. Base of prosternum and neck of prosternal process with distinct ridge, anteriorly smooth, without anterolateral extensions. Blade of prosternal process lanceolate, relatively broad, convex, with distinct bead and few setae; neck and blade of prosternal process evenly jointed. Abdominal sternite 7 broadly rounded apically.

*Male*: Antenna simple (Fig. 21A). Protarsomere 4 with large, thick, strongly curved anterolateral hook. Protarsomere 5 ventrally with anterior row of 16 short setae and posterior row with 7 short setae (Fig. 21B). Abdominal sternite 7 with 6–13 lateral striae on each side. Median lobe with weak submedian constriction in ventral view (Fig. 21C). Paramere with strong notch on dorsal side, with notch tip sharply pointed, and subdistal part elongate, with upper setae thin and more numerous and lower setae shorter, thick, and flattend (Fig. 21E).

*Female*: Traces of the pronotal lateral bead developed stronger in some specimens; abdominal sternite 7 without striae.

#### Distribution.

Papua New Guinea. This species is wide distributed in the Momase Region: Sandaun, East Sepik, Madang, and Morobe Provinces (Fig. 50).

#### Etymology.

The name refers to the village Brahmin where this species was first discovered. The species name is an adjective in the nominative singular.

### 8. Exocelina bundiensis

Shaverdo, Hendrich & Balke sp. n.

urn:lsid:zoobank.org:act:0234AC51-E602-4674-9277-8272A60D32B7

http://species-id.net/wiki/Exocelina_bundiensis

Figs 6A–E , 32

#### Type locality.

Papua New Guinea: Eastern Highlands Province, Akameku-Brahmin, Bismarck Range, 05°54.28'S, 145°22.27'E.

#### Type material.

*Holotype*: male “Papua New Guinea: Eastern Highlands, Akameku-Brahmin, Bismarck Range, 1900m, 23.xi.1994, 05.54.284S, 145.22.271E, Balke & Kinibel (PNG 108)” (ZSM). *Paratypes*: **Eastern Highlands:** 46 males, 19 females with same labels as the holotype, one of them additionally with a green label “DNA M.Balke 1398” (NARI, NHMW, ZSM). 36 males, 25 females “Papua New Guinea: Eastern Highlands, Akameku-Brahmin, Bismarck Range, 1500m, 24.xi.1994, 05.51.964S, 145.23.604E, Balke & Kinibel (PNG 111)” (NHMW, ZSM). **Madang:** 2 males “PAPUA NEW GUINEA: Madang, Bundi small streams Jun 25/91 Larson” (ANIC, NHMW).

#### Diagnosis.

Beetles small, dark brown, dorsally less strongly punctate than *Exocelina hansferyi*, submatt; pronotum with lateral bead; male antennomeres 3–5 enlarged, 6–8 slightly enlarged; male protarsomere 4 with middle-sized, slender, evidently curved anterolateral hook; median lobe with strong submedian constriction and proximal part narrower, apex of median lobe narrower in lateral view; paramere with distinct notch on dorsal side and subdistal part elongate, with less numerous, long, thin setae.

#### Description.

*Size and shape*: Beetle small (TL-H 3.3–3.7 mm, TL 3.6–4.1 mm, MW 1.9–2.1 mm), with oblong-oval habitus, broadest at elytral middle. *Coloration*: Dark brown with reddish brown anterior margin of head, lateral sides and posterior margin of pronotum, head appendages, and legs (Fig. 32).

*Surface sculpture*: Similar to *Exocelina hansferyi*, but with less strong punctation and microreticulation. Head with very dense punctation (spaces between punctures 1–2 times size of punctures), evidently finer and sparser anteriorly; diameter of punctures less than diameter of cells of microreticulation. Pronotum with finer, sparser punctation than on head. Elytra with finer, sparser and more evenly distributed punctation than on pronotum. Pronotum and elytra with rather strongly impressed microreticulation, dorsal surface, thus, submatt. Head with microreticulation stronger. Metaventrite and metacoxa distinctly microreticulate, metacoxal plates with longitudinal strioles and transverse wrinkles. Abdominal sternites with distinct microreticulation, strioles, and fine, rather dense punctation, coarser and denser on two last abdominal sternites.

*Structures*: Pronotum with distinct lateral bead. Base of prosternum and neck of prosternal process with distinct ridge, without anterolateral extensions. Blade of prosternal process lanceolate, rather narrow, slightly convex, with distinct bead and few setae; neck and blade of prosternal process evenly jointed. Abdominal sternite 7 slightly truncate apically.

*Male*: Antennomeres 3–5 enlarged, 6–8 slightly enlarged (Fig. 6A). Protarsomere 4 with middle-sized, slender, evidently curved anterolateral hook. Protarsomere 5 ventrally with anterior row (double apically) of 21 short setae and posterior row of 7 short setae (Fig. 6B). Abdominal sternite 7 with 7–11 lateral striae on each side. Median lobe with strong submedian constriction and proximal part narrower in ventral view, apex of median lobe narrower in lateral view (Figs 6C, D). Paramere with distinct notch on dorsal side and subdistal part elongate, with less numerous, long, thin setae (Fig. 6E).

*Female*: Antennae simple, abdominal sternite 7 without striae.

#### Distribution.

Papua New Guinea. The species is known from Madang and Eastern Highlands Provinces (Fig. 50).

#### Etymology.

The species is named after the village Bundi where it was discovered. The name is an adjective in the nominative singular.

### 9. Exocelina edeltraudae

Shaverdo, Hendrich & Balke sp. n.

urn:lsid:zoobank.org:act:C89AD87B-C775-429E-AC74-706262F679F5

http://species-id.net/wiki/Exocelina_edeltraudae

Figs 4A–F , 30

#### Type locality.

Papua New Guinea: Western Highlands Province, Kurumul, 6 km SW Kudjip, 05°53.43'S, 144°36.60'E.

#### Type material.

*Holotype*: male “Papua New Guinea: Western Highlands, Kurumul, 6Km SW Kudjip, small stream, 1584m, 13.vi.1994, 05.53.426S, 144.36.600E, John (PNG 78)” (ZSM). *Paratypes*: **Western Highlands:** 11 males with the same label as the holotype, one of them additionally with a green label “DNA M.Balke 1341” (NHMW, ZSM). 2 males “Papua New Guinea: Western Highlands, Mt. Hagen town area, 1600m, 7.xii.1994 05.49.745S, 144.22.357E Balke & Kinibel (PNG 131)” (ZSM). **Southern Highlands:** 3 males “PAPUA N.G.: 6.-9.5.1998 Southern Highl. Prov. Tari-Koroba, Hedemari [Hedamali] 1700-1900 m, leg. Riedel” (NHMW). 1 male, 3 females “Papua New Guinea: Southern Highlands, Tari Komo road, 10km N Hides Gas, 1700m, 13.v.1994, Balke (PNG 61)” (ZSM). 12 males, 8 females “Papua New Guinea: Southern Highlands, Tari to Koroba, 1600m, 15.v.1994, 05.46.500S, 142.50.000E, Balke (PNG 65)” (NARI, NHMW, ZSM). 6 males, 1 female “Papua New Guinea: Southern Highlands, Koroba, 1600m, 15.v.1994, 05.41.854S, 142.43.836E, Balke (PNG 66)” (NHMW, ZSM).

#### Diagnosis.

Beetle middle-sized, piceous, submatt; pronotum with distinct lateral bead; male sternite 7 slightly to distinctly concave apically; male antennomeres 3–5 distinctly enlarged, almost equally in size and shape, antennomeres 6–8 enlarged; male protarsomere 4 with middle-sized, slender, evidently curved anterolateral hook; median lobe with very strong submedian constriction and proximal part very broad in ventral view, apex of median lobe pointed and strongly curved downwards in lateral; paramere with distinct notch on dorsal side and subdistal part elongate, with numerous, dense, long, thin setae. The species is well recognizable by the modified antennae of the males and the shape of the median lobe.

#### Description.

*Size and shape*: Beetle middle-sized (TL-H 3.85–4.2 mm, TL 4.15–4.55 mm, MW 2.1–2.55 mm), with oblong-oval habitus, broadest at elytral middle. *Coloration*: Dorsally piceous, with dark brown anterior margin of head and narrowly pronotal sides; head appendages and legs brown, legs distally darker (Fig. 30).

*Surface sculpture*: Head with dense, coarse punctation (spaces between punctures 1–3 times size of punctures), especially on vertex. Pronotum with punctation much finer, sparser, and more evenly distributed than on head. Elytra with extremely sparse and fine punctation. Pronotum and elytra with strongly impressed microreticulation, dorsal surface, thus, submatt. Head with microreticulation stronger. Metaventrite and metacoxa distinctly microreticulate, metacoxal plates with longitudinal strioles and transverse wrinkles. Abdominal sternites with distinct microreticulation, strioles, and fine sparse punctation, coarser and denser on two last abdominal sternites.

*Structures*: Pronotum with distinct lateral bead. Base of prosternum and neck of prosternal process with distinct ridge, rounded and smooth anteriorly, with small anterolateral extensions. Blade of prosternal process lanceolate, relatively narrow, convex, with distinct bead and few setae; neck and blade of prosternal process evenly jointed. Abdominal sternite 7 broadly rounded or concave apically.

*Male*: Antennomeres 3–5 distinctly enlarged, almost equally in size, antennomeres 6–8 enlarged (Fig. 4A), antennomeres 3–7 rugose ventrally. Protarsomere 4 with middle-sized, slender, evidently curved anterolateral hook. Protarsomere 5 ventrally with anterior row of 15 short setae and posterior row of 5 short setae (Fig. 4B). Abdominal sternite 7 with 8–11 lateral striae on each side, slightly to distinctly concave apically (Fig. 4C). Median lobe with very strong submedian constriction and proximal part very broad in ventral view, apex of median lobe pointed and strongly curved downwards in lateral view (Figs 4D, E). Paramere with distinct notch on dorsal side and subdistal part elongate, with numerous, dense, long, thin setae (Fig. 4F).

*Female*: Antennae simple, abdominal sternite 7 broadly rounded apically, without striae.

#### Distribution.

Papua New Guinea. The species is known from Western Highlands and Southern Highlands Provinces (Fig. 50).

#### Etymology.

Dedicated to Edeltraud Tötzl, senior author’s mother-in-law: “With my sincere thankfulness. Without your help with my children, my scientific work was not possible during last six years”. The species name is a noun in the genitive case.

### 10. Exocelina ekari

Shaverdo, Hendrich & Balke sp. n.

urn:lsid:zoobank.org:act:D4B6619E-6B2A-41E7-BC52-81A4C15A9CA2

http://species-id.net/wiki/Exocelina_ekari

Figs 16A–E , 42

Papuadytes ekari Balke, Pons, Ribera, Sagata & Vogler, 2007: 511 (as group name, nomen nudum).

#### Type locality.

Indonesia: Papua Province: Nabire/Paniai Regencies, road Nabire-Enarotali, 55^th^ km, 03°29.80'S, 135°43.89'E. *Note*: the road only goes up to Enarotali, Ilaga is much further in the mountains, therefore people now refer to the road as Nabire-Enarotali.

#### Type material.

*Holotype*: male “IR90-11: W. New Guinea, Trek Nabire-Ilaga, km55, 19.-25.ix.1990, Balke” (NHMW). *Paratypes*: 3 males with the same label as the holotype (ZSM). 1 male ”IRIAN JAYA: Paniai Prov. road Nabire-Ilaga, km 54 10.9.1996, 800m leg. M. Balke (96 # 20)” (NHMW), 2 males “IRIAN JAYA: Paniai Prov. road Nabire-Ilaga, km 54 10.9.1996, 900m leg. M. Balke (96 # 19)” (NHMW).

#### Additional material.

10 females “IRIAN JAYA: Paniai Prov. road Nabire-Ilaga, km 54 10.9.1996, 900m leg. M. Balke (96 # 19)” (NHMW). These females are most likely a mixture of three species: *Exocelina ekari* sp. n., *Exocelina weylandensis* sp. n., and *Exocelina kakapupu* sp .n. Also see the paragraph of *Exocelina irianensis* sp. n.

#### Diagnosis.

Beetle small, dark brown, with slightly paler anterior part of head and pronotal sides, shiny; pronotum without lateral bead; male antennomeres 3–10 stout; male protarsomere 4 with large, thick, strongly curved anterolateral hook; median lobe with strong submedian constriction and apical part very broad in ventral view and slightly flattened in lateral view; paramere with notch on dorsal side and subdistal part short and small, with not numerous, relatively short, thick, and flattened setae.

#### Description.

*Size and shape*: Beetle small (TL-H 3.4–3.7 mm, TL 3.75–4.05 mm, MW 1.75–1.95 mm), with oblong-oval habitus, broadest at elytral middle. *Coloration*: Head dark brown, reddish anteriorly, in one specimen blackish posterior eyes; pronotum dark brown to blackish-brown, with reddish sides or only their anterior parts; elytra dark brown; head appendages yellow to yellowish-red, legs distally darker, hind legs to dark brown (Fig. 42).

*Surface sculpture*: Head with dense punctation (spaces between punctures 1–3 times size of punctures), evidently finer and sparser anteriorly; diameter of punctures smaller than diameter of cells of microreticulation. Pronotum with much finer and sparser punctation than on head. Elytra with very sparse and fine punctation, almost invisible. Head, pronotum, and elytra with weakly impressed microreticulation, dorsal surface, thus, shiny. Head with microreticulation stronger. Metaventrite and metacoxa distinctly microreticulate, metacoxal plates with longitudinal strioles and transverse wrinkles. Abdominal sternites with distinct microreticulation, strioles, and fine sparse punctation, coarser and denser on two last abdominal sternites.

*Structures*: Pronotum without lateral bead or with weak traces of lateral bead. Base of prosternum and neck of prosternal process with distinct ridge, anteriorly rounded, smooth, with very small anterolateral extensions. Blade of prosternal process lanceolate, relatively broad, convex, with distinct bead and few setae; neck and blade of prosternal process evenly jointed. Abdominal sternite 7 broadly rounded apically.

*Male*: Antennomeres 3–10 stout (Fig. 16A). Protarsomere 4 with large, thick, strongly curved anterolateral hook. Protarsomere 5 ventrally with anterior row of 10 short setae and posterior row of 5 short setae (Fig. 16B). Abdominal sternite 7 with 1–5 lateral striae on each side. Median lobe with strong submedian constriction and apical part very broad in ventral view and somehow flattened in lateral view (Figs 16C, D). Paramere with notch on dorsal side and subdistal part short and small, with not numerous, relatively short, thick, and flattened setae (Fig. 16E).

*Female*: Antennae more slender, abdominal sternite 7 without striae.

#### Distribution.

Indonesia: Papua Province: Nabire and Paniai Regencies. This species is known only from the type locality area (Fig. 50).

#### Etymology.

This species is named for the native community (Ekari people) which inhabits the area from which the specimens have been collected. The name is a noun in the nominative standing in apposition.

### 11. Exocelina eme

Shaverdo, Hendrich & Balke sp. n.

urn:lsid:zoobank.org:act:E5E19993-6A44-447F-9B39-5E2DAD8AC9BA

http://species-id.net/wiki/Exocelina_eme

Figs 20A–E , 46

#### Type locality.

Indonesia: Papua Province: Jayawijaya Regency, Emdoman, 04°14'S, 139°55'E.

#### Type material.

*Holotype*: male “IRIAN JAYA: 29.9.1993 Eme Gebiet Emdoman, 800m”, “ca. 139°55'E, 04°14'S leg. M. Balke (24)” (NHMW). *Paratypes*: 1 male, 1 female with the same label as the holotype (NHMW). 1 female “IRIAN JAYA: 29.9.1993 Eme Gebiet Emdoman, 800-1000m”, “ca. 139°55'E, 04°14'S leg. M. Balke (25)”, “DNA M.Balke 3257” [green label] (NHMW).

#### Diagnosis.

Beetle small, piceous, shiny; pronotum without lateral bead; male antennomeres 3-10 slightly stout; male protarsomere 4 with large, thick, strongly curved anterolateral hook; median lobe with weak submedian constriction in ventral view; paramere with notch on dorsal side and subdistal part elongate, with upper setae thin and less numerous and lower setae long, thick, somewhat flattend, and curved at apex.

#### Description.

*Size and shape*: Beetle small (TL-H 3.4–3.45 mm, TL 3.85–3.9 mm, MW 1.75–1.9 mm), with oblong-oval habitus, broadest at elytral middle. *Coloration*: Head piceous, reddish-brown to dark brown anteriorly; pronotum piceous, with reddish-brown to dark brown sides; elytra piceous, with dark brown sutural bands; head appendages yellow to yellowish-red, legs distally darker, to dark brown (Fig. 46).

*Surface sculpture*: Head with dense punctation (spaces between punctures 1–3 times size of punctures), evidently finer and sparser anteriorly; diameter of punctures smaller than diameter of cells of microreticulation. Pronotum with much finer and sparser punctation than on head. Elytra with very sparse and fine punctation, almost invisible. Head, pronotum, and elytra with weakly impressed microreticulation, dorsal surface, thus, shiny. Head with microreticulation stronger. Metaventrite and metacoxa distinctly microreticulate, metacoxal plates with longitudinal strioles and transverse wrinkles. Abdominal sternites with distinct microreticulation, strioles, and fine sparse punctation, coarser and denser on two last abdominal sternites.

*Structures*: Pronotum without lateral bead or with weak traces of lateral bead in females. Base of prosternum and neck of prosternal process with distinct ridge, anteriorly smooth, without anterolateral extensions. Blade of prosternal process lanceolate, narrow, convex, with distinct bead and few setae; neck and blade of prosternal process evenly jointed. Abdominal sternite 7 broadly rounded apically.

*Male*: Antennomeres 3–10 slightly stout (Fig. 20A). Protarsomere 4 with large, thick, strongly curved anterolateral hook. Protarsomere 5 ventrally with anterior row of 14 short setae and posterior row of 6 short setae (Fig. 20B). Abdominal sternite 7 with 5–8 lateral striae on each side. Median lobe with weak submedian constriction in ventral view (Fig. 20C). Paramere with notch on dorsal side and subdistal part elongate, with upper setae thin and less numerous and lower setae long, thick, somewhat flattend, and curved at apex (Fig. 20E).

*Female*: Antenna slightly more slender; pronotum with weak traces of lateral bead; abdominal sternite 7 without striae.

#### Distribution.

Indonesia: Papua Province, Jayawijaya Regency. This species is known only from the type locality (Fig. 50).

#### Etymology.

The species is named for the Eme River, from a tributary of which it has been collected. The name is a noun in the nominative singular standing in apposition.

### 12. Exocelina evelyncheesmanae

Shaverdo, Hendrich & Balke sp. n.

urn:lsid:zoobank.org:act:EB409648-65E2-4022-940A-73515D7FCECF

http://species-id.net/wiki/Exocelina_evelyncheesmanae

Figs 3A–E , 29

#### Type locality.

Indonesia: West Papua Province: Raja Ampat Regency, Waigeo Island, Mountain Nok.

#### Type material.

*Holotype*: male “N.DUTCH NEW GUINEA: Waigeu.Camp 1.Mt.Nok. 2,500 ft. v.1938. L.E.Cheesman. B.M.1938-593.” (BMNH). *Paratypes*: 19 males, 16 females with the same labels as the holotype (BMNH, NHMW). 3 males, 2 females “N.DUTCH NEW GUINEA: Waigeu. Mt.Nok. Camp 2. (Buffelhorn.)vi.1938. L.E.Cheesman. B.M.1938-593.” (BMNH). 22 males, 24 females “N.DUTCH NEW GUINEA: Waigeu. Camp Nok. 2,500 ft. iv.1938. L.E.Cheesman. B.M.1938-593.” (BMNH, NHMW). 18 males, 44 females “Indonesia: Papua, Waigeo, Waifoi, Mt. Nok, 500m, 11.ii.1994, 00.05.076S, 130.44.586E, Balke (BH 11)”, one of them additionally with a green label “DNA M.Balke 1276” (NHMW, ZSM).

#### Diagnosis.

Beetle middle-sized, dark brown to piceous (teneral specimens reddish-brown), shiny; pronotum with lateral bead; male antennomeres 3–7 very slightly enlarged, antennomere 3 slightly more triangular than other antennomeres; male protarsomere 4 with middle-sized, slender, evidently curved anterolateral hook; median lobe with strong submedian constriction in ventral view and truncate apex in lateral view; paramere with notch on dorsal side and subdistal part short and small, with less numerous, relatively short, thick, and flattened setae. The species occurs together with *Exocelina waigeoensis* sp. n. and can be distinguished from it by its larger size (also females) and the shape of the medial lobe.

#### Description.

*Size and shape*: Beetle middle-sized (TL-H 3.75–4.1 mm, TL 4.05–4.45 mm, MW 1.9–2.2 mm), with oblong-oval habitus, broadest at elytral middle. *Coloration*: Head reddish-brown, darker medially and posterior eyes, to piceous, with dark brown anterior margin, pronotum reddish-brown to piceous, paler on sides (in teneral specimens anterior angles yellowish-red), elytra dark brown to piceous, seldom with reddish sutural bands, head appendages yellow to yellowish-red, legs distally darker than head appendages, hind legs dark brown (Fig. 29).

*Surface sculpture*: Head with dense punctation (spaces between punctures 1–4 times size of punctures), evidently finer and sparser anteriorly; diameter of punctures smaller than diameter of cells of microreticulation. Pronotum with finer, sparser, and more evenly distributed punctation than on head. Elytra with very sparse and extremely fine punctation. Pronotum and elytra with weakly impressed microreticulation, dorsal surface, thus, shiny. Head with microreticulation stronger. Metaventrite and metacoxa distinctly microreticulate, metacoxal plates with longitudinal strioles and transverse wrinkles. Abdominal sternites with distinct microreticulation, strioles, and fine sparse punctation, coarser and denser on two last abdominal sternites.

*Structures*: Pronotum with distinct lateral bead, absent in anterior angles. Base of prosternum and neck of prosternal process with distinct ridge, smooth and slightly rounded anteriorly (less than in *Exocelina waigeoensis* sp. n.), without anterolateral extensions. Blade of prosternal process lanceolate, relatively narrow, convex, with distinct bead and few setae; neck and blade of prosternal process evenly jointed. Abdominal sternite 7 broadly rounded apically.

*Male*: Antennomeres 3–7 very slightly enlarged, antennomere 3 slightly more triangular than other antennomeres (Fig. 3A); antennomeres 3–5 rugose ventrally. Protarsomere 4 with middle-sized, slender, evidently curved anterolateral hook. Protarsomere 5 ventrally with anterior row of 13 short setae and posterior row of 5–6 short setae (Fig. 3B). Abdominal sternite 7 with 5–12 lateral striae on each side. Median lobe with strong submedian constriction in ventral view and truncate apex in lateral view (Figs 3C, D). Paramere with notch on dorsal side and subdistal part short and small, with less numerous, relatively short, thick, and flattened setae (Fig. 3E).

*Female*: Antennae more slender, abdominal sternite 7 without striae.

#### Distribution.

Indonesia: West Papua Province: Raja Ampat Regency. The species is known only from the type locality (Fig. 50).

#### Etymology.

The species is named for the incredible collector and adventurer, Miss Lucy Evelyn Cheesman who discovered this species. The species name is a noun in the genitive case.

### 13. Exocelina hansferyi

Shaverdo, Hendrich & Balke sp. n.

urn:lsid:zoobank.org:act:E61F6212-054D-4010-B44F-9138A8A48026

http://species-id.net/wiki/Exocelina_hansferyi

Figs 5A-E , 31

#### Type locality.

Papua New Guinea: Morobe Province, Herzog Mts., Wagau, 06°51.07'S, 146°48.07'E.

#### Type material.

*Holotype*: male “Stn. No. 150.”, “NEW GUINEA: Morobe Dist., Herzog Mts., Vagau, C. 4,000 ft. 4-17.i.1965”, “M.E. Bacchus. B. M. 1965-120” (BMNH). *Paratypes*: 21 males, 13 females with the same labels as the holotype (BMNH, NHMW, ZSM).

#### Diagnosis.

Beetle small, yellowish-red to brown, dorsally strongly punctate, submatt; pronotum with lateral bead; male antennomeres 3–5 enlarged, 6–8 slightly enlarged; male protarsomere 4 with middle-sized, slender, evidently curved anterolateral hook; median lobe with strong submedian constriction and proximal part narrower, apex of median lobe rather broad in lateral view; paramere with shallow notch on dorsal side and subdistal part elongate, with less numerous, long, thin setae.

#### Description.

*Size and shape*: Beetle small (TL-H 3.45–3.75 mm, TL 3.8–4.1 mm, MW 1.9–2.1 mm), with oblong-oval habitus, broadest at elytral middle. *Coloration*: Teneral beetles yellow to yellowish red, mature ones reddish-brown to brown with reddish anterior part of head, pronotal sides, and sutural bands of elytra; head appendages yellow to red, legs darker distally, especially hind legs (Fig. 31).

*Surface sculpture*: Head with very dense punctation (spaces between punctures 1–2 times size of punctures), evidently finer and sparser anteriorly; diameter of punctures equal diameter of cells of microreticulation. Pronotum and elytra with finer, sparser, and more evenly distributed punctation than on head. Pronotum and elytra with rather strongly impressed microreticulation, dorsal surface, thus, submatt. Head with microreticulation stronger. Metaventrite and metacoxa distinctly microreticulate, metacoxal plates with longitudinal strioles and transverse wrinkles. Abdominal sternites with distinct microreticulation, strioles, and fine, rather dense punctation, coarser and denser on two last abdominal sternites.

*Structures*: Pronotum with distinct lateral bead. Base of prosternum and neck of prosternal process with distinct ridge, smooth and slightly rounded anteriorly, with small anterolateral extensions. Blade of prosternal process lanceolate, rather narrow, slightly convex, with distinct bead and few setae; neck and blade of prosternal process evenly jointed. Abdominal sternite 7 slightly truncate apically.

**Figure 5.**
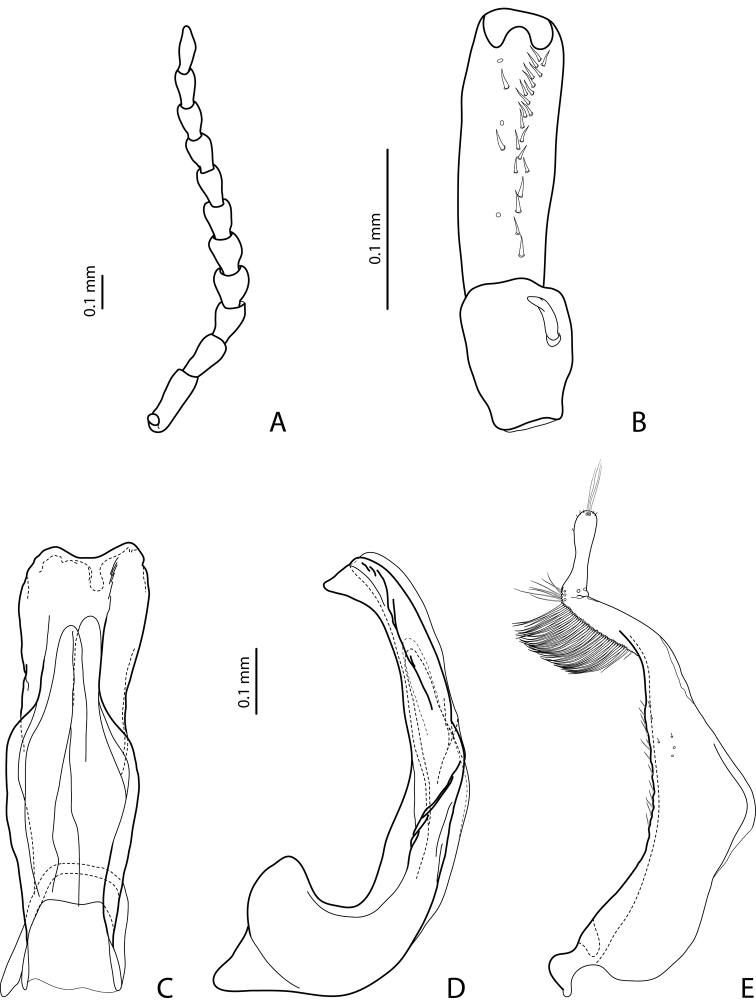
*Exocelina hansferyi* sp. n. **A** male antenna **B** protarsomeres 4–5 in ventral view **C** median lobe in ventral view **D** median lobe in lateral view **E** paramere in external view.

*Male*: Antennomeres 3–5 enlarged, 6–8 slightly enlarged (Fig. 5A). Protarsomere 4 with middle-sized, slender, evidently curved anterolateral hook. Protarsomere 5 ventrally with anterior row (double apically) of 21 short setae and posterior row of 5 short setae (Fig. 5B). Abdominal sternite 7 with 3–8 lateral striae on each side. Median lobe with strong submedian constriction and proximal part narrower in ventral view, apex of median lobe rather broad in lateral view (Figs 5C, D). Paramere with shallow notch on dorsal side and subdistal part elongate, with less numerous, long, thin setae (Fig. 5E).

*Female*: Antennae simple, abdominal sternite 7 without striae.

#### Distribution.

Papua New Guinea: Morobe Province. The species is known only from the type locality (Fig. 50).

#### Etymology.

The species is named after our friend and colleague Dr. Hans Fery (Berlin). The name is a noun in the genitive case.

### 14. Exocelina irianensis

Shaverdo, Hendrich & Balke sp. n.

urn:lsid:zoobank.org:act:9B389DDF-9732-4BFC-B539-212FFDB16D5F

http://species-id.net/wiki/Exocelina_irianensis

Figs 12A–E , 38

#### Type locality.

Indonesia: Papua Province: Nabire/Paniai Regencies, road Nabire-Enarotali, 54^th^ km, 03°29.52'S, 135°43.91'E.

#### Type material.

*Holotype*: male “West New Guinea/Paniai Prov./IR19 track Nabire-Ilaga km 54 Basecamp, 750–800m, 16.–27.7.1991 leg: Balke & Hendrich” (CLH). *Paratypes*: 7 males, 6 females with the same label as the holotype (CLH). 8 males “IR90-11: W. New Guinea, Trek Nabire-Ilaga, km55, 19.–25.ix.1990, Balke” (ZSM). 9 males “W.-Neuguinea/Paniai Prov. Strasse Nabire-Ilaga km 54 700m, 22.–25.9.1990/IR 11 leg: Balke & Hendrich” (ZSM, CLH). 3 males “IRIAN JAYA: Paniai Prov. road Nabire-Ilaga, km 54 26./27.8.1996, 750–800m leg. M. Balke (96 # 2)” (NHMW). 1 male “IRIAN JAYA: Paniai Prov. road Nabire-Ilaga, km 54 30.8.1996, 750m leg. M. Balke (96 # 9)”, “DNA M.Balke 3264” [green] (NHMW). 31 males “IRIAN JAYA: Paniai Prov. road Nabire-Ilaga, km 117 Unipo, 2.9.1996, 150m leg. M. Balke (96 # 12)” (NHMW). 4 males “IRIAN JAYA: Paniai Prov. road Nabire-Ilaga, km 54 10.9.1996, 800m leg. M. Balke (96 # 20)”, 1 male of them with a green label “DNA M.Balke 3265” (NHMW). 3 males, 2 females “IRIAN JAYA: Nabire Prov. Nabire-Ilaga, km 35 Kali Cemara, 27.9.1997 leg. M. Balke (# 5)” (NHMW). 1 male, 1 female “IRIAN JAYA: Nabire Prov. rd. Nabire-Ilaga, Km 54 ca. 750m, X.1997 leg. Balke” (NHMW). 5 exs. “Irian Jaya: Nabire distr., road Nabire-Ilaga, km 54, 03 29'517"S, 135 43'913"E, 750m, iv.1998 M. Balke leg.” (NHMW). 13 males “Indonesia: Papua, Road Nabire-Enarotali KM 55, 774m, 22.x.2011, 03 29.796S, 135 43.885E, Uncen (PAP09)”, two of them additionally with labels “DNA M. Balke 4906”, “DNA M. Balke 4907” (NHMW, ZSM). 1 male “Indonesia: Papua, Road Nabire-Enarotali KM 80, 250m, 22.x.2011, 03 33.860S, 135 46.473E, Uncen (PAP12)” (ZSM). 1 male “Indonesia: Papua, Road Nabire-Enarotali KM 95, 160m, 22.x.2011, 03 34.193S, 135 49.246E, Uncen (PAP13)” (ZSM). 12 males “Indonesia: Papua, Road Nabire-Enarotali KM 52, 555m, 23.x.2011, 03 30.107S, 135 42.971E, Uncen (PAP17)” (MZB, NHMW, ZSM). 1 male, 1 female “Indonesia: Papua, Road Nabire-Enarotali KM 40, 350m, 23.x.2011, 03 29.314S, 135 41.188E, Uncen (PAP18)” (ZSM).1 female “Indonesia: Papua, Road Nabire-Enarotali KM 62, 340m, 22.x.2011, 03 31.684S, 135 42.802E, Uncen (PAP11)”, “DNA M. Balke 4917”, *note*: accessory of this female to the species is based on DNA data (MZB).

#### Additional material.

29 females “IRIAN JAYA: Paniai Prov. road Nabire-Ilaga, km 117 Unipo, 2.9.1996, 150m leg. M. Balke (96 # 12)” (NHMW). These females are most likely a mixture of two species: *Exocelina irianensis* sp. n. and *Exocelina unipo* sp. n. 13 females “Indonesia: Papua, Road Nabire-Enarotali KM 55, 774m, 22.x.2011, 03 29.796S, 135 43.885E, Uncen (PAP09)” (NHMW, ZSM). These females are most likely a mixture of two species: *Exocelina irianensis* sp. n. and *Exocelina weylandensis* sp. n. 12 females “Indonesia: Papua, Road Nabire-Enarotali KM 80, 250m, 22.x.2011, 03 33.860S, 135 46.473E, Uncen (PAP12)” (NHMW, ZSM). These females are most likely a mixture of three species: *Exocelina irianensis* sp. n., *Exocelina soppi* sp. n., and one additional new species. 13 females “Indonesia: Papua, Road Nabire-Enarotali KM 95, 160m, 22.x.2011, 03 34.193S, 135 49.246E, Uncen (PAP13)” (NHMW, ZSM). These females are most likely a a mixture of two species: *Exocelina irianensis* sp. n. and *Exocelina unipo* sp. n. 21 females “Indonesia: Papua, Road Nabire-Enarotali KM 52, 555m, 23.x.2011, 03 30.107S, 135 42.971E, Uncen (PAP17)” (NHMW, ZSM). These females are most likely a mixture of two species: *Exocelina irianensis* sp. n. and *Exocelina soppi* sp. n. 9 females “IRIAN JAYA: Paniai Prov. road Nabire-Ilaga, km 54 10.9.1996, 800m leg. M. Balke (96 # 20)”. These females are most likely a mixture of four species: *Exocelina irianensis* sp. n., *Exocelina ekari* sp. n., *Exocelina weylandensis* sp. n., and *Exocelina kakapupu* sp. n. 8 females “IRIAN JAYA: Paniai Prov. road Nabire-Ilaga, km 54 26./27.8.1996, 750–800m leg. M. Balke (96 # 2)” (NHMW). These females are most likely a mixture of two species: *Exocelina irianensis* sp. n. and *Exocelina kakapupu* sp. n. 8 females “W.-Neuguinea/Paniai Prov. Strasse Nabire-Ilaga km 54 700m, 22.–25.9.1990/IR 11 leg: Balke & Hendrich” (ZSM, CLH). These females are most likely a mixture of three species: *Exocelina irianensis* sp. n., *Exocelina weylandensis* sp. n., and *Exocelina soppi* sp. n.

#### Diagnosis.

Beetle small, reddish-brown to dark brown, usually with paler head and pronotal sides; shiny; pronotum without lateral bead; male antennomeres 3–5 distinctly enlarged; male protarsomere 4 with large, thick, evidently curved anterolateral hook; median lobe with very strong submedian constriction, distal and proximal parts equally broad, and symmetrical apex in ventral view; paramere with shallow notch on dorsal side and subdistal part short and small, with not numerous, relatively short, thick, and flattened setae. The species differs from other ones by the pronotum without lateral bead and modified antennae of the males.

#### Description.

*Size and shape*: Beetle small (TL-H 3.2–3.85 mm, TL 3.55–4.25 mm, MW 1.7–2.05 mm), with oblong-oval habitus, broadest at elytral middle. *Coloration*: Head uniformly reddish to dark brown, in a few specimens darker posteriorly; pronotum reddish-brown to dark brown, with paler sides, in a few specimens uniformly colored; elytra uniformly reddish-brown to dark brown; head appendages yellowish-red, legs darker (Fig. 38).

*Surface sculpture*: Head with dense punctation (spaces between punctures 1–3 times size of punctures), evidently finer and sparser anteriorly; diameter of punctures smaller than diameter of cells of microreticulation. Pronotum with a little sparser but much finer punctation than on head, almost invisible. Elytra with extremely sparse and fine punctation. Head, pronotum, and elytra with weakly impressed microreticulation, dorsal surface, thus, shiny. Head with microreticulation stronger. Metaventrite and metacoxa distinctly microreticulate, metacoxal plates with longitudinal strioles and transverse wrinkles. Abdominal sternites with distinct microreticulation, strioles, and fine sparse punctation, coarser and denser on two last abdominal sternites.

*Structures*: Pronotum without lateral bead, in some specimens with indistinct traces of bead. Base of prosternum and neck of prosternal process with distinct ridge, without anterolateral extensions. Blade of prosternal process lanceolate, relatively broad, slightly convex, with distinct bead and few setae; neck and blade of prosternal process evenly jointed. Abdominal sternite 7 broadly truncate apically, in some specimens very distinctly, in some less.

*Male*: Antennomeres 3 and 4 strongly enlarged, evidently larger than other, antennomere 5 distinctly enlarged, 6–9 robust (Fig. 12A); antennomeres 3–6 strongly and 7–9 somewhat rugose ventrally. Protarsomere 4 with large, thick, evidently curved anterolateral hook. Protarsomere 5 ventrally with anterior row of 9 short setae and posterior row of 5 short setae (Fig. 12B). Abdominal sternite 7 with 4–7 lateral striae on each side. Median lobe with very strong submedian constriction, distal and proximal parts equally broad, and symmetrical apex in ventral view (Figs 12C). Paramere with shallow notch on dorsal side and subdistal part short and small, with not numerous, relatively short, thick, and flattened setae (Fig. 12E).

*Female*: Antenna simple; traces of bead on pronotal sides are more often observed than in males; abdominal sternite 7 without striae.

#### Distribution.

Indonesia: Papua Province. This species is known from Nabire and Paniai Regencies (Fig. 50).

#### Etymology.

The name is derived from the Biak (northern coast of New Guinea) islanders’ word “Irian”, which means “hot island emerging from the sea” and refers to New Guinea. The species name is an adjective in the nominative singular.

### 15. Exocelina kakapupu

Shaverdo, Hendrich & Balke sp. n.

urn:lsid:zoobank.org:act:C54CD308-6991-4787-ACF7-5C5C2504B596

http://species-id.net/wiki/Exocelina_kakapupu

Figs 22A–E , 48

#### Type locality.

Indonesia: Papua Province: Nabire/Paniai Regencies, road Nabire-Enarotali, 54^th^ km, 03°29.52'S, 135°43.91'E.

#### Type material.

*Holotype*: male “IRIAN JAYA: Paniai Prov. road Nabire-Ilaga, km 54 26./27.8.1996, 750-800m leg. M. Balke (96 # 2)” (NHMW). *Paratypes*: 7 males with the same label as the holotype (NHMW). 2 males “IR 24-W. New Guinea, track Nabire-Ilaga KM 54, basecamp 750m, 25.vii.1991 Balke &Hendrich leg.” (NHMW, ZSM). 1 male “West New Guinea/Paniai Prov./IR 24 track Nabire-Ilaga km 54 basecamp, 750m, 25.7.1991 leg: Balke &Hendrich” (CLH). 1 male “West New Guinea/Paniai Prov./IR 19 track Nabire-Ilaga km 54 Basecamp, 750-800m, 16.-27.7.1991 leg: Balke & Hendrich” (CLH). 1 male “IRIAN JAYA: Paniai Prov. road Nabire-Ilaga, km 54 10.9.1996, 800m leg. M. Balke (96 # 20)” (NHMW), 1 male “IRIAN JAYA: Paniai Prov. road Nabire-Ilaga, km 54 10.9.1996, 900m leg. M. Balke (96 # 19)” (NHMW). 20 males “Indonesia: Papua, Road Nabire-Enarotali KM 60, 640m, 22.x.2011, 03 30.474S 135 42.611E, Uncen (PAP10)”, one of them additionally with a label “DNA M. Balke 4912” (MZB, NHMW, ZSM).

#### Additional material.

12 females “Indonesia: Papua, Road Nabire-Enarotali KM 60, 640m, 22.x.2011, 03 30.474S 135 42.611E, Uncen (PAP10)”, two of them additionally with labels “DNA M. Balke 4909”, “DNA M. Balke 4913” (NHMW, ZSM). These females most likely belong to *Exocelina kakapupu* sp. n. However they are not included in the type series, since four specimens of *Exocelina weylandensis* sp. n. have been collected from this locality too, and, therefore, some of females might belong to this species. Also see in the paragraph of *Exocelina irianensis* sp. n. and *Exocelina ekari* sp. n.

#### Diagnosis.

Beetle small, very similar to *Exocelina soppi* sp. n. and *Exocelina pseudosoppi* sp. n. in size and coloration; pronotum without lateral bead; male antennomeres simple; male protarsomere 4 with large, thick, strongly curved anterolateral hook; median lobe with strong submedian constriction in ventral view and almost truncate apex in lateral view; paramere with notch on dorsal side and elongate subdistal part, with numerous, dense, long, thin setae; setae of proximal part longer, thicker, distinctly visible.

#### Description.

*Size and shape*: Beetle small (TL-H 3.15–3.4 mm, TL 3.55–3.8 mm, MW 1.7–1.85 mm), with oblong-oval habitus, broadest at elytral middle. *Coloration*: as in *Exocelina soppi* sp. n. and *Exocelina pseudosoppi* sp. n (Fig. 48).

*Surface sculpture*: Punctation as in *Exocelina soppi* sp. n. and *Exocelina pseudosoppi* sp. n. but denser and coarser; microreticulation evidently stronger than in these species.

*Structures*: Pronotum without lateral bead or with weak traces of lateral bead. Base of prosternum and neck of prosternal process with distinct ridge, anteriorly rounded, smooth, with very small anterolateral extensions. Blade of prosternal process lanceolate, relatively broad, slightly convex, with distinct bead and few setae; neck and blade of prosternal process evenly jointed. Abdominal sternite 7 broadly truncate apically.

*Male*: Antenna simple (Fig. 22A). Protarsomere 4 with large, thick, strongly curved anterolateral hook. Protarsomere 5 ventrally with anterior row of 13 short setae and posterior row of 3 short setae (Fig. 22B). Abdominal sternite 7 with 2–7 lateral striae on each side. Median lobe with strong submedian constriction in ventral view and almost truncate apex in lateral view (Figs 22C, D). Paramere with notch on dorsal side and elongate subdistal part, with numerous, dense, long, thin setae; setae of proximal part longer, thicker, distinctly visible (Fig. 22E).

*Female*: Without evident differences in external morphology from male, except for abdominal sternite 7 without striae.

#### Distribution.

Indonesia: Papua Province: Nabire and Paniai Regencies. This species is known only from the type locality area (Fig. 50).

#### Etymology.

The Ekari people sometimes called smaller water beetles “kakapupu” (Hendrich and Balke 1992). The species name is a noun in the nominative singular standing in apposition.

### 16. Exocelina knoepfchen

Shaverdo, Hendrich & Balke sp. n.

urn:lsid:zoobank.org:act:24B7B2E7-50F3-46EF-879B-29662C53BD82

http://species-id.net/wiki/Exocelina_knoepfchen

Figs 7A–E , 33

#### Type locality.

Papua New Guinea: Eastern Highlands Province, Kainantu, Yoginofi, 06°21.80'S, 145°45.46'E.

#### Type material.

*Holotype*: male “Papua New Guinea: Eastern Highlands, Kainantu, Yoginofi, 1900m, 9.v.1994, 06.21.799S, 145.45.463E, Balke & Sagata (PNG 55)” (ZSM). *Paratypes*: 40 males, 23 females with the same label as the holotype (NARI, NMHW, ZSM), 1 male, 1 female “VI 79 PNG/EHProv. Umg. Kainantu Onerunka” (NHMW). 1 male “IV 79 PNG/EHProv. Umg. Kainantu Onerunka” (NHMW). 9 males, 5 females “Papua New Guinea: Eastern Highlands, Onerunka, small creek, red soil/rock, 1700 m 21.v.1994, 06.02936S 145.46.874E, John & Balke (PNG 71)” (NHMW, ZSM), one male additionally with a green label “DNA M.Balke 1303”, 1 male “X 79 PNG/EHProv. Um. Kainantu” (NHMW). 1 female “18 VI 79 PNG/EHProv. Umg. Ofafina Jababari Riv.” (NHMW). 9 males, 2 females “Papua New Guinea: Aiyura, 1787m, 15.i.2003, 06.21.411S, 145.54.340E, K. Sagata, (WB5)” (ZSM). 3 males, 4 females “Papua New Guinea: Eastern Highlands, Aiyura, 1670m, 5.iv.1994, 06.21.131S, 145.54.398E, Balke & Sagata (PNG 32)” (ZSM). 6 males, 5 females “Papua New Guinea: Eastern Highlands, Aiyura, ditch in forest, 1670 m, 20.v.1994, 06.21.131S, 145.54.398E, John & Balke (PNG 69)” (NHMW, ZSM). 13 males, 11 females “Papua New Guinea: Eastern Highlands, Aiyura creek, 1670 m, 20.v.1994, 06.21.131S, 145.54.398E, John & Balke (PNG 70)” (NHMW, ZSM). 1 male “Papua New Guinea: Eastern Highlands, Bena-pass to Goroka valley, 1550m, 5.iv.1994, 06.14.567S, 145.29.643E, Balke & Sagata (PNG 33)” (ZSM). 2 males, 3 females “Papua New Guinea: Eastern Highlands, Hogu, 1 km E Mt. Barola, 1900m, 9.v.1994, 06.17.556S, 145.45.036E, Balke & Sagata (PNG 56)” (ZSM).

#### Diagnosis.

Beetle middle-sized, uniformly dark brown or head and pronotum slightly paler; pronotum with distinct lateral bead; male antennomere 3 evidently larger than other; male protarsomere 4 with very small (smaller than more laterally situated large seta), thin, slightly curved anterolateral hook; median lobe with very weak submedian constriction, apex of median lobe almost rounded in lateral view; paramere without notch on dorsal side, with relatively short, sparse, thin setae. The species is well recognizable by its larger size, the modified antennae of the males, and paramere distinctly longer than median lobe.

#### Description.

*Size and shape*: Beetle middle-sized (TL-H 4.5–4.8 mm, TL 4.95–5.3 mm, MW 2.35–2.55 mm), with oblong-oval habitus, broadest at elytral middle.* Coloration*: Dorsally uniformly dark brown or head and pronotum slightly paler, with paler (yellowish-red to reddish-brown) anterior margin of head, lateral sides of pronotum, and narrow bands along elytral suture; head appendages yellowish-red to reddish-brown, legs slightly darker (Fig. 33).

*Surface sculpture*: Head with dense punctation (spaces between punctures 1–3 times size of punctures), evidently finer and sparser anteriorly; diameter of punctures smaller than diameter of cells of microreticulation. Pronotum with finer, sparser, and more evenly distributed punctation than on head. Elytra with very sparse and fine punctation, almost invisible. Head, pronotum, and elytra with weakly impressed microreticulation, dorsal surface, thus, shiny. Head with microreticulation stronger. Metaventrite and metacoxa distinctly microreticulate, metacoxal plates with longitudinal strioles and transverse wrinkles. Abdominal sternites with distinct microreticulation, strioles, and fine sparse punctation, coarser and denser on two last abdominal sternites.

*Structures*: Pronotum with distinct lateral bead. Base of prosternum and neck of prosternal process with strong, sharp ridge, without anterolateral extensions. Blade of prosternal process lanceolate, narrow, convex, with distinct bead and few setae; neck and blade of prosternal process evenly jointed. Abdominal sternite 7 broadly rounded apically.

*Male*: Antennomere 3 strongly enlarged, evidently larger than other, antennomere 4 distinctly enlarged (Fig. 7A); antennomeres 3 and 4 rugose ventrally. Protarsomere 4 with very small (smaller than more laterally situated large seta), thin, slightly curved anterolateral hook. Protarsomere 5 ventrally with anterior row (double apically) of 19 short setae and posterior row of 6 short setae (Fig. 7B). Abdominal sternite 7 with 6–11 lateral striae on each side. Median lobe with very weak submedian constriction in ventral view, apex of median lobe almost rounded in lateral view (Figs 7C, D). Paramere distinctly longer than median lobe, without notch on dorsal side, with relatively short, sparse, thin setae (Fig. 7E).

*Female*: Antennae simple, abdominal sternite 7 without striae.

#### Distribution.

Papua New Guinea. This species is known from Eastern Highlands Province (Fig. 50).

#### Etymology.

The species is named for an old friend of M. Balke. The name is a noun in the nominative singular standing in apposition.

### 17. Exocelina munaso

(Shaverdo, Sagata & Balke, 2005)

http://species-id.net/wiki/Exocelina_munaso

Fig. 24

Papuadytes munaso Shaverdo, Sagata & Balke, 2005: 276.

#### Type locality.

Papua New Guinea: Simbu/Eastern Highlands Provinces, Crater Mountain, Wara Sera Station, 06°43.4'S, 145°05.6'E.

#### Type material examined.

*Holotype*:male “PAPUA NEW GUINEA Simbu/EHPr. Crater Mountain, Wara Sera Station, 800 m, 14IX2002, Balke & Sagata (PNG 10)”, “255 DNA M Balke” [green label] (BMNH). *Paratypes*: 1 male “PNG Simbu/EHPr. Crater Mountain, Sera-Herowana, Wara Hulene, 1000 m, 16IX2002, Balke & Sagata (PNG 17)”, “262 DNA M Balke” [green label] (NHMW).

#### Additional material.

1 female “Papua New Guinea: Simbu/EHP. Crater Mountain, Sera-Herowana, Hulene river, 1000m, 16IX2002, Balke & Sagata (PNG 017)” (ZSM). 1 male “Papua New Guinea: Crater Mountain, Sera-Herowana, Hulene river, 1000m, 16.IX2002, Balke & Sagata (PNG 017)” (ZSM). 2 females “Papua New Guinea: Crater Mountain, Wara Sera Station, 800m, 14IX2002, Balke & Sagata, (PNG 010)” (NHMW, ZSM). 1 male “PAPUA NEW GUINEA Simbu/EHPr. Crater Mountain, Wara Sera Station, 800m, 14IX2002, Balke & Sagata, (PNG 10)” (NHMW). 1 female “Papua New Guinea: Simbu/EHP, Crater Mountain, Wara Sera Station, 800m, 14IX2002, Balke & Sagata, (PNG 009)” (ZSM).

#### Diagnosis.

Beetle middle-sized (TL-H 4.8–5.0 mm, TL 5.1–5.3 mm, MW 2.6 mm), piceous, dull (Fig. 24); pronotum with distinct lateral bead; male antennomeres simple; male protarsomere 4 with large, thick, strongly curved anterolateral hook; median lobe without submedian constriction but with lateral folds in ventral view and apex pointed and curved downwards in lateral view; paramere without notch on dorsal side, with relatively dense, long, thin setae.

#### Additions and corrections to the description.

For the complete description and illustrations see Shaverdo et al. (2005).

*Structures*: Pronotum with distinct lateral bead. Base of prosternum and neck of prosternal process with sharp ridge and well developed anterolateral extensions. Blade of prosternal process lanceolate, rather narrow, with strong longitudinal convexity; neck and blade of prosternal process evenly jointed, except for weak concavity in front of jointion of protrochanters.

*Male*: Protarsomere 4 with large, thick, strongly curved anterolateral hook. Median lobe without submedian constriction but with lateral folds in ventral view and with apex pointed and curved downwards in lateral view. Paramere without notch on dorsal side, with relatively dense, long, thin setae. See Figs 10, 15a, b in Shaverdo et al. (2005).

*Female*: Without evident differences in external morphology from male, except for abdominal sternite 7 without striae.

#### Distribution.

Papua New Guinea. The species is known only from Crater Mountain, Simbu and Eastern Highlands Provinces (Fig. 50).

### 18. Exocelina oceai

Shaverdo, Hendrich & Balke sp. n.

urn:lsid:zoobank.org:act:4692229C-7DC6-4A67-84F1-7D86FD09772F

http://species-id.net/wiki/Exocelina_oceai

Figs 1A–E , 26

#### Type locality.

Indonesia: Papua Province: Nabire/Paniai Regencies, road Nabire-Enarotali, 65^th^ km, 05°46.50'S, 142°50.00'E.

#### Type material.

*Holotype*: male “IRIAN JAYA: Paniai Prov. road Nabire-Ilaga, km 65 29.8.1996, 250m leg. M. Balke (96 # 6)” (NHMW). *Paratypes*: 14 males, 23 females with the same label as the holotype, 2 females additionally with green labels “DNA M.Balke 3262”, “DNA M.Balke 3263” (NHMW). 2 males, 1 female “IRIAN JAYA: Paniai Prov. road Nabire-Ilaga, km 80 12.9.1996, 250m leg. M. Balke (96 # 21)” (NHMW). 1 male “IR 23-W. New Guinea, track Nabire-Ilaga, KM 62, 250m, 24.vii.1991 Balke & Hendrich leg.” (NHMW). 1 male “IR 21-W. New Guinea track Nabire-Ilaga KM 65, Kali Utowa, 250 M, 18-19.vii.1991 Balke & Hendrich leg.” (ZSM). 1 male “W.-Neuguinea/Paniai Prov. Strasse Nabire-Ilaga km 54 700m, 22.-25.9.1990/IR 11 leg: Balke & Hendrich” (CLH).

#### Diagnosis.

Beetle small, reddish-brown to brown; pronotum with narrow lateral bead; male antennomeres simple; male protarsomere 4 with large, thick, strongly curved anterolateral hook; median lobe slender, with weak submedian constriction in ventral view and elongate apex in lateral view; paramere with strong notch on dorsal side and subdistal part short and large, with long, dense, curved at apex setae.

#### Description.

*Size and shape*: Beetle small (TL-H 3.35–3.8 mm, TL 3.75–4.2 mm, MW 1.8–2.05 mm), with oblong-oval habitus, broadest at elytral middle. *Coloration*: Head reddish to reddish-brown, paler anteriorly (especially on clypeus); pronotum reddish-brown, with paler sides and darker (dark brown) disc, in some specimens almost uniformly reddish, reddish-brown; elytron uniformly reddish-brown to dark brown, darker than head and pronotum or only than head; head appendages yellowish-red, legs reddish (Fig. 26).

*Surface sculpture*: Head with dense punctation (spaces between punctures 1–4 times size of punctures), evidently finer and sparser anteriorly; diameter of punctures smaller than diameter of cells of microreticulation. Pronotum with finer, sparser, and more evenly distributed punctation than on head. Elytra with very sparse and fine punctation, almost invisible. Pronotum and elytra with weakly impressed microreticulation, dorsal surface, thus, shiny. Head with microreticulation stronger. Metaventrite and metacoxa distinctly microreticulate, metacoxal plates with longitudinal strioles and transverse wrinkles. Abdominal sternites with distinct microreticulation, strioles, and fine sparse punctation, coarser and denser on two last abdominal sternites.

*Structures*: Pronotum with distinct but narrow lateral bead, in some specimens reduced at posterior angles. Base of prosternum and neck of prosternal process with distinct ridge, smooth and rounded anteriorly, without anterolateral extensions. Blade of prosternal process lanceolate, relatively broad, convex, with distinct bead and few setae; neck and blade of prosternal process evenly jointed. Abdominal sternite 7 broadly rounded apically.

*Male*: Antenna simple (Fig. 1A). Protarsomere 4 with large, thick, strongly curved anterolateral hook. Protarsomere 5 ventrally with anterior row of 10 short setae and posterior row of 4 setae (Fig. 1B). Abdominal sternite 7 with 13–17 lateral striae on each side. Median lobe slender, with weak submedian constriction in ventral view and elongate apex in lateral view (Figs 1C, D). Paramere with strong notch on dorsal side and subdistal part short and large, with long, dense, curved at apex setae (Fig. 1E).

*Female*: Some specimens with pronotal lateral bead reduced at posterior angles; abdominal sternite 7 without striae.

#### Distribution.

Indonesia: Papua Province: Nabire and Paniai Regencies. This species is known only from the type locality area (Fig. 50).

#### Etymology.

The species is named for “Doc” Ocea Megay, one of our Ekari fellows and most enthusiastic fieldworker who died from a snake bite shortly after we have collected this species. He is buried in Topo. The species name is a noun in the genitive case.

### 19. Exocelina polita

(Sharp, 1882)

http://species-id.net/wiki/Exocelina_polita

Figs 11A–E , 37

Copelatus politus Sharp, 1882: 568.
Copelatus politus Sharp, 1882: Régimbart 1899: 292–293 (descr.); Zimmermann 1919: 198 (cat.), 1920: 145 (cat.); Guéorguiev 1968: 34 (cat.), 1978: 269 (key); Nilsson 2001: 66 (cat.).
Exocelina polita (Sharp, 1882): Nilsson and Fery 2006: 56 (n. comb.).

#### Type locality.

Indonesia: West Papua Province: Manokwari Regency, Arfak Mts., Hatam. *Note*: Hatam is situated in the Arfak Mountains north of Ransiki (A. Riedel, pers. comment).

#### Type material.

*Lectotype* (hereby designated): male “Type” [round, with red rim], “Hatam N. Guinea Luglio [= July] 1875. Beccari.” [hw, Beccari], “Sharp Coll 1905-313”, “Hatam, New-Guinea July 1875 Beccari 660” [hw, Sharp], “Type 660 Copelatus politus n. sp. New Guinea” [hw, Sharp], “Lectotype Copelatus politus Sharp des. Shaverdo, Hendrich & Balke 2012” [red, printed] (BMNH). *Notes*: The lectotype is designated in order to support the stability of nomenclature since it is not clear from the original description that it has been based on the single male. The genialia are partly damaged.

#### Diagnosis.

Beetle middle-sized, brown, shiny, with almost invisible dorsal punctation; pronotum with distinct lateral bead; male antennomeres 3–4 strongly enlarged and triangular (3 distinctly larger than 4), 5–6 distinctly enlarged, 7 somewhat enlarged; male protarsomere 4 with small, thin, slightly curved anterolateral hook; median lobe with strong submedian constriction in ventral view, apex of median lobe elongate in lateral view and broader in ventral view; paramere with notch on dorsal side and subdistal part short and small, with not numerous, relatively short, thick, and flattened setae.

#### Redescription.

*Size and shape*: Beetle middle-sized (TL-H 3.9 mm, TL 4.35 mm, MW 2.15 mm), with oblong-oval habitus, broadest at elytral middle. *Coloration*: Dorsally dark brown, with reddish head, pronotal sides, and sutural bands on elytra; head appendages yellowish red, legs darker, especially hind legs (Fig. 37).

*Surface sculpture*: Head with dense punctation (spaces between punctures 1–2 times size of punctures), evidently finer and sparser anteriorly; diameter of punctures smaller or equal than diameter of cells of microreticulation. Pronotum with distinctly finer, sparser, and more evenly distributed punctation than on head. Elytra with very sparse and fine punctation, almost invisible. Head, pronotum, and elytra with strongly impressed microreticulation, dorsal surface shiny. Head with microreticulation stronger. Metaventrite and metacoxa distinctly microreticulate, metacoxal plates with longitudinal strioles and transverse wrinkles. Abdominal sternites with distinct microreticulation, strioles, and fine sparse punctation, coarser and denser on two last abdominal sternites.

*Structures*: Pronotum with distinct lateral bead. Base of prosternum and neck of prosternal process with distinct ridge, rounded and smooth anteriorly, with very small anterolateral extensions. Blade of prosternal process lanceolate, relatively broad, convex, with distinct bead and few setae; neck and blade of prosternal process evenly jointed. Abdominal sternite 7 broadly rounded apically.

*Male*: Antennomeres 3–4 strongly enlarged and triangular (3 distinctly larger than 4), 5–6 distinctly enlarged, 7 somewhat enlarged (Fig. 11A); antennomeres 3–7 rugose ventrally. Protarsomere 4 with small, thin, slightly curved anterolateral hook. Protarsomere 5 ventrally with anterior row of 12 short setae and posterior row 5 short setae (Fig. 11B). Abdominal sternite 7 with 3–4 lateral striae on each side. Median lobe with strong submedian constriction in ventral view, apex of median lobe elongate in lateral view and broader in ventral view (Figs 11C, D). Paramere with notch on dorsal side and subdistal part short and small, with not numerous, relatively short, thick, and flattened setae (Fig. 11E).

*Female*: Unknown.

#### Distribution.

Indonesia: West Papua Province: Manokwari Regency. The species is known only from the lectotype from the Arfak Mountains, the eastern part of Bird’s head (Fig. 50).

### 20. Exocelina pseudosoppi

Shaverdo, Hendrich & Balke sp. n.

urn:lsid:zoobank.org:act:85647A40-EF25-4B70-B9E7-4FA422734981

http://species-id.net/wiki/Exocelina_pseudosoppi

Figs 19A–E , 45

#### Type locality.

Indonesia: Papua Province: Nabire/Paniai Regencies, road Nabire-Enarotali, 80^th^ km, 03°33.86'S, 135°46.47'E.

#### Type material.

*Holotype*: male “IRIAN JAYA: Paniai Prov. road Nabire-Ilaga, km 80 12.9.1996, 250m leg. M. Balke (96 # 21)” (NHMW). *Paratypes*: 1 male, 2 females with the same label as the holotype (NHMW). 1 male “Indonesia: Papua, Road Nabire-Enarotali KM 62, 340m, 22.x.2011, 03 31.684S 135 42.802E, Uncen (PAP11)”, “DNA M. Balke 4916” (ZSM).

#### Diagnosis.

Beetle small, very similar to *Exocelina soppi* sp. n., differing from it in the shape of the anterior part of the prosternum, simple male antennae, and male genitalia: truncate apex of the median lobe, stronger submedian constriction, and the shape and setation of the paramere: shallow notch on the dorsal side and subdistal part elongate, with a strong tuft of thicker, somewhat flattend, and strongly curved at apex setae.

#### Description.

*Size and shape*: Beetle small (TL-H 3.15 mm, TL 3.45–3.5 mm, MW 1.65–1.7 mm), with oblong-oval habitus, broadest at elytral middle. *Coloration*: Similar to *Exocelina soppi* sp. n. (Fig. 45).

*Surface sculpture*: Punctation and microreticulation as in *Exocelina soppi* sp. n.

*Structures*: Pronotum without lateral bead. Base of prosternum and neck of prosternal process with distinct ridge, anteriorly evidently less rounded and smooth than in *Exocelina soppi* sp. n., with very small anterolateral extensions. Blade of prosternal process lanceolate, relatively broad, slightly convex, with distinct bead and few setae; neck and blade of prosternal process evenly jointed. Abdominal sternite 7 broadly rounded apically.

*Male*: Antenna simple (Fig. 19A). Protarsomere 4 with middle-sized, slender, evidently curved anterolateral hook. Protarsomere 5 ventrally with anterior row of 15 short setae and posterior row of 4 short setae (Fig. 19B). Abdominal sternite 7 with 3–7 lateral striae on each side. Median lobe with strong submedian constriction in ventral view and truncate apex in lateral view (Figs 19C, D). Paramere with shallow notch on dorsal side and subdistal part elongate, with strong tuft of thicker, somewhat flattend, and strongly curved at apex setae (Fig. 19E).

*Female*: Without evident differences in external morphology from male, except for abdominal sternite 7 without striae.

#### Distribution.

Indonesia: Papua Province: Nabire and Paniai Regencies. This species is known only from the type locality area (Fig. 50).

#### Etymology.

Long time this species was mistaken for *Exocelina soppi* sp. n. The name is a noun in the nominative singular standing in apposition.

### 21. Exocelina soppi

Shaverdo, Hendrich & Balke sp. n.

urn:lsid:zoobank.org:act:A9510374-6CC3-481B-8136-EE8E732DDCFA

http://species-id.net/wiki/Exocelina_soppi

Figs 18A–E , 44

#### Type locality.

Indonesia: Papua Province: Nabire/Paniai Regencies, road Nabire-Enarotali, 80^th^ km, 03°33.86'S, 135°46.47'E.

#### Type material.

*Holotype*: male “IRIAN JAYA: Paniai Prov. road Nabire-Ilaga, km 80 12.9.1996, 250m leg. M. Balke (96 # 21)” (NHMW). *Paratypes*: 2 males, 1 female with the same label as the holotype, the female additionally with “DNA M.Balke 3260” (NHMW). 4 males “IR90-11: W. New Guinea, Trek Nabire-Ilaga, km55, 19.-25.ix.1990, Balke” (ZSM, NHMW). 4 males “W.-Neuguinea/Paniai Prov. Straße Nabire-Ilaga km 54 700m, 22.-25.9.1990/IR 11 leg: Balke & Hendrich” (CLH). 1 male “Indonesia: Papua, Road Nabire-Enarotali KM 80, 250m, 22.x.2011, 03 33.860S 135 46.473E, Uncen (PAP12)”, “DNA M. Balke 4910” (ZSM). 1 male “Indonesia: Papua, Road Nabire-Enarotali KM 52, 555m, 23.x.2011, 03 30.107S 135 42.971E, Uncen (PAP17)”, “DNA M. Balke 4911” (MZB).

#### Additional material.

See in the paragraph of *Exocelina irianensis* sp. n.

#### Diagnosis.

Beetle small, dark brown, often with paler head and pronotal sides, shiny; pronotum without lateral bead; male antennomeres 3–10 slightly stout; male protarsomere 4 with middle-sized, slender, evidently curved anterolateral hook; median lobe with weak submedian constriction in ventral view and elongate apex in lateral view; paramere with notch on dorsal side and subdistal part short and small, with large brush of thick, somewhat flattend, long, curved at apex setae.

#### Description.

*Size and shape*: Beetle small (TL-H 3.0–3.4 mm, TL 3.35–3.85 mm, MW 1.6–1.8 mm), with oblong-oval habitus, broadest at elytral middle. *Coloration*: Head reddish brown to dark brown; pronotum dark brown, with reddish sides; elytra uniformly dark brown, except narrow reddish sutural bands in some specimens; head appendages yellowish-red to reddish, legs darker, especially metathoracic legs; beetles generally paler if teneral (Fig. 44).

*Surface sculpture*: Head with dense punctation (spaces between punctures 1–3 times size of punctures), evidently finer and sparser anteriorly; diameter of punctures smaller than diameter of cells of microreticulation. Pronotum with much sparser and finer punctation than on head. Elytra with extremely sparse and fine punctation. Head, pronotum, and elytra with weakly impressed microreticulation, dorsal surface, thus, shiny. Head with microreticulation stronger. Metaventrite and metacoxa distinctly microreticulate, metacoxal plates with longitudinal strioles and transverse wrinkles. Abdominal sternites with distinct microreticulation, strioles, and fine sparse punctation, coarser and denser on two last abdominal sternites.

*Structures*: Pronotum without lateral bead. Base of prosternum and neck of prosternal process with distinct ridge, anteriorly rounded, smooth, with very small anterolateral extensions. Blade of prosternal process lanceolate, relatively broad, slightly convex, with distinct bead and few setae; neck and blade of prosternal process evenly jointed. Abdominal sternite 7 broadly rounded apically, in some species broadly truncate.

*Male*: Antennomeres 3–10 slightly stout (Fig. 18A). Protarsomere 4 with middle-sized, slender, evidently curved anterolateral hook. Protarsomere 5 ventrally with anterior row of 9 short setae and posterior row of 3 short setae (Fig. 18B). Abdominal sternite 7 with 3–7 lateral striae on each side. Median lobe with weak submedian constriction in ventral view and elongate apex in lateral view (Figs 18C, D). Paramere with notch on dorsal side and subdistal part short and small, with large brush of thick, somewhat flattend, long, curved at apex setae (Fig. 18E).

*Female*: Antennae slightly more slender, abdominal sternite 7 without striae.

#### Distribution.

Indonesia: Papua Province: Nabire and Paniai Regencies. This species is known only from the type locality area (Fig. 50).

#### Etymology.

This species is dedicated to our old buddy Michael Sopp (Berlin). The species name is a noun in the genitive case.

### 22. Exocelina unipo

Shaverdo, Hendrich & Balke sp. n.

urn:lsid:zoobank.org:act:92EF40EC-63DA-4CDA-8946-23B98C96C0E7

http://species-id.net/wiki/Exocelina_unipo

Figs 23A–E , 49

#### Type locality.

Indonesia: Papua Province: Nabire/Paniai Regencies, road Nabire-Enarotali, 117^th^ km, Unipo, approximately 03°31.83'S, 135°55.98'E.

#### Type material.

*Holotype*: male “IRIAN JAYA: Paniai Prov. road Nabire-Ilaga, km 117 Unipo, 2.9.1996, 150m leg. M. Balke (96 # 12)” (NHMW). *Paratypes*: 5 males with the same label as the holotype (NHMW). 5 males “Indonesia: Papua, Road Nabire-Enarotali KM 95, 160m, 22.x.2011, 03 34.193S 135 49.246E, Uncen (PAP13)” (NHMW, ZSM). 1 male, 3 females “Indonesia: Papua, Road Nabire-Enarotali KM 113, 150m, 23.x.2011, 03 31.827S 135 55.975E, Uncen (PAP14)” (MZB, ZSM). 1 male “Indonesia: Papua, Road Nabire-Enarotali KM 111, 100m, 23.x.2011, 03 31.192S 135 55.426E, Uncen (PAP15)” (ZSM). 22 males, 9 females “Indonesia: Papua, Road Nabire-Enarotali KM 108, 140m, 23.x.2011, 03 30.258S 135 54.840E, Uncen (PAP16)”, 1 male and 1 female with labels “DNA M. Balke 4902”, “DNA M. Balke 4903” (NHMW, ZSM).

#### Additional material.

See in the paragraph of *Exocelina irianensis* sp. n.

#### Diagnosis.

Beetles small, very similar to *Exocelina kakapupu* sp. n. except for male antennomeres 3–10 stout, male genitalia, weaker dorsal punctation and microreticulaton, and much more striated male sternite 7. Also protarsomere 4 with large, thick, strongly curved anterolateral hook, which apical part longer than in *Exocelina kakapupu* sp. n. Median lobe with weak submedian constriction in ventral view and elongate apex in lateral view; paramere with notch on dorsal side and elongate subdistal part, with numerous, dense, long, thin setae; setae of proximal part shorter, thiner, often hardly visible.

#### Description.

*Size and shape*: Beetles small (TL-H 3.0–3.5 mm, TL 3.35–3.9 mm, TW 1.6–1.85 mm), with oblong-oval habitus, broadest at elytral middle. *Coloration*: Head reddish-brown, reddish anteriorly; pronotum reddish-brown, with reddish sides; elytra dark brown, with paler (reddish-brown) sutural bands; head appendages yellowish, legs darker distally (reddish-brown) (Fig. 49).

*Surface sculpture*: Punctation and microreticulation evidently weaker than in *Exocelina kakapupu* sp. n., more similar to that in *Exocelina soppi* sp. n.

*Structures*: Pronotum without lateral bead or with weak traces of lateral bead. Base of prosternum and neck of prosternal process with distinct ridge, anteriorly rounded, with weak transverse lines, small anterolateral extensions. Blade of prosternal process lanceolate, relatively broad, slightly convex, with distinct bead and few setae; neck and blade of prosternal process evenly jointed. Abdominal sternite 7 broadly rounded apically.

*Male*: Antennomeres 3–10 stout (Fig. 23A). Protarsomere 4 with large, thick, strongly curved anterolateral hook, its apical part longer than in *Exocelina kakapupu* sp. n. Protarsomere 5 ventrally with anterior row of 12 short setae and posterior row of 6 short setae (Fig. 23B). Abdominal sternite 7 with 15 lateral striae on each side. Median lobe with weak submedian constriction in ventral view and elongate apex in lateral view (Figs 23C, D). Paramere with notch on dorsal side and elongate subdistal part, with numerous, dense, long, thin setae; setae of proximal part shorter, thiner, often hardly visible (Fig. 23E).

*Female*: Antennae more slender, abdominal sternite 7 without striae.

#### Distribution.

Indonesia: Papua Province: Nabire and Paniai Regencies. This species is known only from the type locality area (Fig. 50).

#### Etymology.

The species is named for the type locality. The name is a noun in the nominative singular standing in apposition.

### 23. Exocelina utowaensis

Shaverdo, Hendrich & Balke sp. n.

urn:lsid:zoobank.org:act:CD2B8904-6AE3-4D4A-BC61-07F0C6296A28

http://species-id.net/wiki/Exocelina_utowaensis

Figs 14A–F , 40

#### Type locality.

Indonesia: Papua Province: Nabire/Paniai Regencies, road Nabire-Enarotali, 80^th^ km, 03°33.86'S, 135°46.47'E.

#### Type material.

*Holotype*: male “IRIAN JAYA: Paniai Prov. road Nabire-Ilaga, km 80 12.9.1996, 200m leg. M. Balke (96 # 22)” (NHMW). *Paratypes*: 23 males, 12 females with the same label as the holotype (NHMW). 3 males, 4 females “IRIAN JAYA: Paniai Prov. road Nabire-Ilaga, km 80 1.9.1996, 200m leg. M. Balke (96 # 10)” (NHMW). 12 males, 6 females “IRIAN JAYA: Nabire Prov. Nabire-Ilaga, km 35 Kali Cemara, 27.9.1997 leg. M. Balke (96 # 5)” (NHMW). 7 males, 4 females “IRIAN JAYA: Paniai Prov. road Nabire-Ilaga, km 65 29.8.1996, 250m leg. M. Balke (96 # 7)” (NHMW). 15 males, 10 females “IR 21-W. New Guinea, track Nabire-Ilaga KM 65, Kali Utowa, 250M, 18.-19.vii.1991 Balke & Hendrich leg.” (ZSM, NHMW). 8 males, 14 females “West New Guinea/Paniai Prov./IR 21 track Nabire-Ilaga km 65 Kali Utowa, 250m, 18.&19.7.1991 leg: Balke & Hendrich” (CLH). 1 male “IR 20-W. New Guinea, track Nabire-Ilaga KM 59, ca.750m, 18.vii.1991, Balke & Hendrich leg.” (ZSM). 4 males, 7 females “West New Guinea/Paniai Prov./IR 22 track Nabire-Ilaga km 62 250m, 24.7.1991, forest pools leg: Balke & Hendrich” (CLH). 3 exs. “Indonesia: Papua, Road Nabire-Enarotali KM 55, 774m, 22.x.2011, 03 29.796S 135 43.885E, Uncen (PAP09)”, two of them additionally with labels “DNA M. Balke 4914”, “DNA M. Balke 4915”, (NHMW, ZSM). 24 exs. “Indonesia: Papua, Road Nabire-Enarotali KM 62, 340m, 22.x.2011, 03 31.684S 135 42.802E, Uncen (PAP11)”, two of them additionally with labels “DNA M. Balke 4904”, “DNA M. Balke 4905”, (NHMW, ZSM). 16 exs. “Indonesia: Papua, Road Nabire-Enarotali KM 95, 160m, 22.x.2011, 03 34.193S 135 49.246E, Uncen (PAP13)” (MZB, NHMW, ZSM). 3 exs. “Indonesia: Papua, Road Nabire-Enarotali KM 111, 100m, 23.x.2011, 03 31.192S 135 55.426E, Uncen (PAP15)” (NHMW, ZSM).

#### Diagnosis.

Beetle small, piceous, shiny; pronotum without lateral bead; male antennomeres 3–10 slightly enlarged; sternite 7 concave; male protarsomere 4 with large, thick, strongly curved anterolateral hook; median lobe long, with very weak submedian constriction and apex narrow in ventral view; paramere large, with strong notch on dorsal side and subdistal part very broad, subquadrate, with dense, long, relatively thick, curved at apex setae. The species is well recognizable by its characteristic male genitalia and concave (also in females) abdominal sternite 7.

#### Description.

*Size and shape*: Beetle small (TL-H 3.4–3.8 mm, TL 3.85–4.1 mm, MW 1.85–2.05 mm), with oblong-oval habitus, broadest at elytral middle. *Coloration*: Head uniformly piceous or with dark brown anterior part; pronotum piceous, with reddish-brown anterior parts of sides, yellowish in anterior angles; elytra uniformly piceous or with narrow dark brown sutural bands; head appendages yellow, legs distally darker (reddish-brown), hind legs to dark brown; teneral specimens dark brown (Fig. 40).

*Surface sculpture*: Head with dense punctation (spaces between punctures 1–3 times size of punctures), evidently finer and sparser anteriorly; diameter of punctures smaller than diameter of cells of microreticulation. Pronotum with much finer and sparser punctation than on head. Elytra with extremely sparse and fine punctation. Head, pronotum, and elytra with weakly impressed microreticulation, dorsal surface, thus, shiny. Head with microreticulation stronger. Metaventrite and metacoxa distinctly microreticulate, metacoxal plates with longitudinal strioles and transverse wrinkles. Abdominal sternites with distinct microreticulation, strioles, and fine sparse punctation, coarser and denser on two last abdominal sternites.

*Structures*: Pronotum without lateral bead. Base of prosternum and neck of prosternal process with distinct ridge, anteriorly smooth, without anterolateral extensions. Blade of prosternal process lanceolate, narrow, convex, with distinct bead and few setae; neck and blade of prosternal process evenly jointed. Abdominal sternite 7 concave apically.

*Male*: Antennomeres 3–10 slightly enlarged (Fig. 14A). Protarsomere 4 with large, thick, strongly curved anterolateral hook. Protarsomere 5 ventrally with anterior row of 15 short setae and posterior row of 4 short setae (Fig. 14B). Abdominal sternite 7 very distinctly concave apically, with 8–17 lateral striae on each side (Fig. 14C). Median lobe long, with very weak submedian constriction and apex narrow in ventral view (Figs 14D, E). Paramere large, with strong notch on dorsal side and subdistal part very broad, subquadrate, with dense, long, relatively thick, curved at apex setae (Fig. 14F).

*Female*: Antenna distinctly more slender than in male; abdominal sternite 7 only slightly concave apically, without striae.

#### Distribution.

Indonesia: Papua Province: Nabire and Paniai Regencies. This species is known from the lower Utowa River area and one of its tributaries (Fig. 50).

#### Etymology.

The species is named for Utowa River from which many specimens have been collected. The name is an adjective in the nominative singular.

### 24. Exocelina waigeoensis

Shaverdo, Hendrich & Balke sp. n.

urn:lsid:zoobank.org:act:DE6E08E6-5106-4F93-8E02-FF7C167A152D

http://species-id.net/wiki/Exocelina_waigeoensis

Figs 2A–E , 28

#### Type locality.

Indonesia: West Papua Province: Raja Ampat Regency, Waigeo Island, Mountain Nok.

#### Type material.

*Holotype*: male “N.DUTCH NEW GUINEA: Waigeu. Mt.Nok. Camp 2. (Buffelhorn.)vi.1938. L.E.Cheesman. B.M.1938-593.” (BMNH). *Paratypes*: 8 males with the same labels as the holotype (BMNH, NHMW). 13 males “N.DUTCH NEW GUINEA: Waigeu. Camp Nok. 2,500 ft. iv.1938. L.E.Cheesman. B.M.1938-593.”, one of them additionally with labels “collection 26”, “measured J.Parkin 77” (BMNH, NHMW, ZSM). 2 males “N.DUTCH NEW GUINEA: Waigeu.Camp 1.Mt.Nok. 2,500 ft. v.1938. L.E.Cheesman. B.M.1938-593.” (BMNH).

#### Additional material.

27 females “N.DUTCH NEW GUINEA: Waigeu. Camp Nok. 2,500 ft. iv.1938. L.E.Cheesman. B.M.1938-593.”, one of them additionally with labels “collection 27”, “measured J.Parkin 76” (BMNH). 6 females “N.DUTCH NEW GUINEA: Waigeu.Camp 1.Mt.Nok. 2,500 ft. v.1938. L.E.Cheesman. B.M.1938-593.” (BMNH). These females are a mixture of two species: *Exocelina waigeoensis* sp. n. and another new species, which are impossible to distinguish.

#### Diagnosis.

Beetle small, reddish-brown, shiny; pronotum with distinct lateral bead; male antennomeres 3–7 very slightly enlarged, antennomere 3 slightly more triangular than other antennomeres; male protarsomere 4 with middle-sized, slender, evidently curved anterolateral hook; median lobe with strong submedian constriction in ventral view and elongate apex in lateral view; paramere with notch on dorsal side and subdistal part short and small, with less numerous, relatively short, thick, and flattened setae.

#### Description.

*Size and shape*: Beetle small (TL-H 3.45–3.7 mm, TL 3.75–4.1 mm, MW 1.8–2.0 mm), with oblong-oval habitus, broadest at elytral middle. *Coloration*: Head and pronotum uniformly reddish-brown, darker posterior eyes and sometimes on anterior margin of pronotum, elytra dark brown, head appendages yellow to yellowish-red, legs distally darker than head appendages, hind legs to reddish-brown (Fig. 28). *Note*: Perhaps, the coloration can be darker: the type series includes several teneral beetles and it is possible that the rest specimens are not completely sclerotized.

*Surface sculpture*: Head with dense punctation (spaces between punctures 1–4 times size of punctures), evidently finer and sparser anteriorly; diameter of punctures smaller than diameter of cells of microreticulation. Pronotum with finer, sparser, and more evenly distributed punctation than on head. Elytra with very sparse and extremely fine punctation. Pronotum and elytra with weakly impressed microreticulation, dorsal surface, thus, shiny. Head with microreticulation stronger. Metaventrite and metacoxa distinctly microreticulate, metacoxal plates with longitudinal strioles and transverse wrinkles. Abdominal sternites with distinct microreticulation, strioles, and fine sparse punctation, coarser and denser on two last abdominal sternites.

*Structures*: Pronotum with distinct lateral bead, absent in anterior angles. Base of prosternum and neck of prosternal process with distinct ridge, smooth and rounded anteriorly, without anterolateral extensions. Blade of prosternal process lanceolate, relatively broad, slightly convex, with distinct bead and few setae; neck and blade of prosternal process evenly jointed. Abdominal sternite 7 broadly rounded apically.

*Male*: Antennomeres 3–7 very slightly enlarged, antennomere 3 slightly more triangular than other antennomeres (Fig. 2A); antennomeres 3–5 rugose ventrally. Protarsomere 4 with middle-sized, slender, evidently curved anterolateral hook. Protarsomere 5 ventrally with anterior row of 9 short setae and posterior row of 5 short setae (Fig. 2B). Abdominal sternite 7 with 3–8 lateral striae on each side. Median lobe with strong submedian constriction in ventral view and elongate apex in lateral view (Figs 2C, D). Paramere with notch on dorsal side and subdistal part short and small, with less numerous, relatively short, thick, and flattened setae (Fig. 2E).

*Female*: Antennae more slender, abdominal sternite 7 without striae.

#### Distribution.

Indonesia: West Papua Province: Raja Ampat Regency. The species is known only from the type locality (Fig. 50).

#### Etymology.

The species is named in reference to its distribution: Waigeo Island. The name is an adjective in the nominative singular.

### 25. Exocelina weylandensis

Shaverdo, Hendrich & Balke sp. n.

urn:lsid:zoobank.org:act:B8F60F6A-55F6-456A-9160-94E8B2D76A5A

http://species-id.net/wiki/Exocelina_weylandensis

Figs 17A–E , 43

#### Type locality.

Indonesia: Papua Province: Nabire/Paniai Regencies, road Nabire-Enarotali, 55^th^ km, 03°29.80'S, 135°43.89'E.

#### Type material.

*Holotype*: male “IR90-11: W. New Guinea, Trek Nabire-Ilaga, km55, 19- 25.ix.1990, Balke” (NHMW). *Paratypes*: 8 males with the same label as the holotype (NHMW, ZSM), 1 male “IR 11” [hw] (ZSM). 6 males “W.-Neuguinea/Paniai Prov. Straße Nabire-Ilaga km 54 700m, 22.-25.9.1990/IR 11 leg: Balke & Hendrich” (CLH). 1 male “West New Guinea/Paniai Prov./IR 19 track Nabire-Ilaga km 54 Basecamp, 750-800m, 16.-27.7.1991 leg: Balke & Hendrich” (CLH). 1 male, 2 females “West New Guinea/Paniai Prov./IR 24 track Nabire-Ilaga km 54 Basecamp, 750m, 25.7.1991 leg: Balke & Hendrich” (CLH). 12 males “IRIAN JAYA: Paniai Prov. road Nabire-Ilaga, km 54 10.9.1996, 900m leg. M. Balke (96 # 19)”, one of them additionally with a green label “DNA M.Balke 3259” (NHMW). 1 male “IRIAN JAYA: Paniai Prov. road Nabire-Ilaga, km 54 10.9.1996, 800m leg. M. Balke (96 # 20)” (NHMW). 17 males “Indonesia: Papua, Road Nabire-Enarotali KM 55, 774m, 22.x.2011, 03 29.796S 135 43.885E, Uncen (PAP09)” (MZB, NHMW, ZSM). 4 males “Indonesia: Papua, Road Nabire-Enarotali KM 60, 640m, 22.x.2011, 03 30.474S 135 42.611E, Uncen (PAP10)”, one of them additionally with a label “DNA M. Balke 4908” (ZSM).

#### Additional material.

See in the paragraph of *Exocelina irianensis* sp. n., *Exocelina ekari* sp. n., and *Exocelina kakapupu* sp. n.

#### Diagnosis.

Beetle small, reddish-brown to brown, with paler head and pronotal sides, shiny; pronotum without lateral bead; male antennomeres 3–10 stout; protarsomere 4 with middle-sized, slender, evidently curved anterolateral hook; median lobe slender, with very weak submedian constriction in ventral view; paramere with notch on dorsal side and subdistal part short and small, with not numerous, relatively short, thick, flattened, slightly curved at apex setae.

#### Description.

*Size and shape*: Beetle small (TL-H 3.15–3.5 mm, TL 3.5–3.9 mm, MW 1.65–1.85 mm), with oblong-oval habitus, broadest at elytral middle. *Coloration*: Head reddish to dark brown, usually paler anteriorly; pronotum dark brown, with reddish sides; elytra uniformly dark brown, except narrow reddish sutural bands; head appendages yellowish-red, legs darker, especially metathoracic legs (Fig. 43).

*Surface sculpture*: Punctation and microreticulation as in *Exocelina irianensis* sp. n.

*Structures*: Pronotum without lateral bead, in a very few specimens with indistinct traces of bead. Base of prosternum and neck of prosternal process with distinct ridge, without anterolateral extensions. Blade of prosternal process lanceolate, relatively broad, slightly convex, with distinct bead and few setae; neck and blade of prosternal process evenly jointed. Abdominal sternite 7 broadly rounded apically.

*Male*: Antennomeres 3–10 stout (Fig. 17A). Protarsomere 4 with middle-sized, slender, evidently curved anterolateral hook. Protarsomere 5 ventrally with anterior row of 14 short setae and posterior row of 5 short setae (Fig. 17B). Abdominal sternite 7 with 3–8 lateral striae on each side. Median lobe slender, with very weak submedian constriction in ventral view (Fig. 17C). Paramere with notch on dorsal side and subdistal part short and small, with not numerous, relatively short, thick, flattened, slightly curved at apex setae (Fig. 17E).

*Female*: Antennae more slender, abdominal sternite 7 without striae.

#### Distribution.

Indonesia: Papua Province: Nabire and Paniai Regencies. This species is known only from the type locality area (Fig. 50).

#### Etymology.

Derived from the name of the range, Weyland, at the northern edge of which the type locality is situated. The species name is an adjective in the nominative singular.

### 26. Exocelina wondiwoiensis

Shaverdo, Hendrich & Balke sp. n.

urn:lsid:zoobank.org:act:6743E99F-4D3E-46C5-8CA5-C89135BA9A63

http://species-id.net/wiki/Exocelina_wondiwoiensis

Figs 13A–E , 39

#### Type locality.

Indonesia: West Papua Province: Teluk Wondama Regency, Wandammen Peninsula, Wondiwoi Mts., Wasior, 2°45.94'S, 134°31.74'E.

#### Type material.

*Holotype*: male “Indonesia: West Papua: Wandammen, Wasior, 4-5.I.2001, leg. A. Riedel 2?[°]45.940'S 134?[°]31.738'E” (ZSM). *Paratypes*: 11 males, 20 females with same label as the holotype, 2 males with additional green labels “56 DNA M Balke”, “57 DNA M Balke” (NHMW, ZSM). 4 males, 6 females “IRIAN JAYA: Kabup. Nabire Wandammen penins. Wondiwoi-Mts., 29.-30.7.1998 6h from Yeretua, 560 m leg. M. Balke (WA 9)” (NHMW). 8 males, 9 females “IRIAN JAYA: Wandammen Bay, Wondiwoi Mts. Wasior, 250-600 m, 4.I.2001 leg. A. RIEDEL” (SMNS, ZSM, NHMW).

#### Diagnosis.

Beetle small, externally very similar to *Exocelina irianensis* sp. n. but darker: dark brown to piceous, with head (in some specimens only its anterior part) paler; shiny; pronotum without lateral bead; male antennomeres 3–5 distinctly enlarged; male protarsomere 4 with large, thick, evidently curved anterolateral hook; median lobe with very strong submedian constriction, distal and proximal parts equally broad, and asymmetrical apex in ventral view; paramere with shallow notch on dorsal side and subdistal part short and small, with not numerous, relatively short, thick, and flattened setae. The species is very similar to the previous one and differs from it only by the assymetrical shape of the apex of the median lobe.

#### Description.

*Size and shape*: Beetle small (TL-H 3.45–3.8 mm, TL 3.8–4.25 mm, MW 1.8–2.05 mm), with oblong-oval habitus, broadest at elytral middle. *Coloration*: Head reddish to piceous, with reddish anterior part; pronotum reddish to piceous, with reddish sides; elytra uniformly dark brown to piceous, in paler specimens with narrow reddish-brown sutural bands; head appendages yellowish-red, legs darker, metathoracic legs to dark brown (Fig. 39).

*Surface sculpture*: Dorsal punctation as in *Exocelina irianensis* sp. n. but slightly coarser, especially on head. Head, pronotum, and elytra with weakly impressed microreticulation, dorsal surface, thus, shiny. Head with microreticulation stronger. Metaventrite and metacoxa distinctly microreticulate, metacoxal plates with longitudinal strioles and transverse wrinkles. Abdominal sternites with distinct microreticulation, strioles, and fine sparse punctation, coarser and denser on two last abdominal sternites.

*Structures*: Pronotum without lateral bead, in some specimens with indistinct traces of lateral bead. Base of prosternum and neck of prosternal process with distinct ridge, without anterolateral extensions. Blade of prosternal process lanceolate, relatively broad, slightly convex, with distinct bead and few setae; neck and blade of prosternal process evenly jointed. Abdominal sternite 7 broadly truncate apically.

*Male*: Antennomeres 3 and 4 strongly enlarged, evidently larger than other, antennomere 5 distinctly enlarged, 6–9 robust (Fig. 13A); antennomeres 3–6 strongly and 7–9 somewhat rugose ventrally. Protarsomere 4 with large, thick, evidently curved anterolateral hook. Protarsomere 5 ventrally with anterior row of 11 short setae and posterior row with 5 short setae (Fig. 13B). Abdominal sternite 7 with 7–8 lateral striae on each side. Median lobe with very strong submedian constriction, distal and proximal parts equally broad, and asymmetrical apex in ventral view (Fig. 13C). Paramere with shallow notch on dorsal side and subdistal part short and small, with not numerous, relatively short, thick, and flattened setae (Fig. 13E).

*Female*: Antenna simple; traces of bead on pronotal sides are more often observed than in males; abdominal sternite 7 without striae.

#### Distribution.

Indonesia: West Papua Province: Teluk Wondama Regency. This species is known from the Wondiwoi Mountains of Wandammen Peninsula (Fig. 50).

#### Etymology.

The species is named for the type area, Wondiwoi Mountains. The name is an adjective in the nominative singular.

##### Key to species of the *Exocelina ekari*-grou*p*

The key is based mostly on the male characters. In many cases females cannot be assigned to species due to similarity of their external and internal structures (for female genitalia see Figs 17a and 17b in Shaverdo et al. (2005). Some species are rather similar in point of external morphology, therefore, in most cases the male genitalia need to be studied for reliable species identification. Numbers in brackets refer to an arrangement of the species descriptions above.

**Table:** 

1	Pronotum with distinct lateral bead, broad or narrow	2
–	Pronotum without lateral bead or with weak traces of lateral bead	15
2	Male antennomeres simple or slightly modified: antennomere 3–7 very slightly enlarged (almost indistinctly), antennomere 3 slightly more triangular than other antennomeres	3
–	Male antennomeres 3–5 evidently enlarged	8
3	Beetle larger, TL-H: 3.9–5.0 mm, piceous	4
–	Beetle smaller, TL-H: 3.35–4.1 mm, reddish-brown to piceous	5
4	Beetle larger, TL-H: 4.8–5.0 mm (Fig. 24), male protarsomere 4 with large, thick, strongly curved anterolateral hook, apex of median lobe pointed and curved downwards in lateral view (Figs 10, 15a in Shaverdo et al. (2005)	(17) *Exocelina munaso*
–	Beetle smaller, TL-H: 3.9–4.1 mm (Fig. 25), male protarsomere 4 with middle-sized, slender, evidently curved anterolateral hook, apex of median lobe almost rounded in lateral view (Figs 9, 14a in Shaverdo et al. (2005)	(5) *Exocelina atowaso*
5	Beetle smaller, TL-H: 3.35–3.8 mm (Fig. 26), male antenna simple, male protarsomere 4 with large, thick, strongly curved anterolateral hook, median lobe more slender in ventral view, and paramere with strong notch on dorsal side and subdistal part short and large (Fig. 1)	(18) *Exocelina oceai* sp. n.
–	Beetle larger, TL-H: 3.45–4.1 mm (Figs 27–29), male antenna simple or slightly modified, male protarsomere 4 with middle-sized, evidently curved anterolateral hook, median lobe broader in ventral view, and paramere different	6
6	Beetle dorsally submatt, with distinct punctation (Fig. 27), male antennomeres simple, median lobe short and with extremely strongly discontinuous (curved, plicate) outline, paramere with shallow notch on dorsal side and subdistal part elongate, with dense, long, thin setae (Figs 37, 46, 64 in Balke (1998)	(4) *Exocelina astrophallus*
–	Beetle dorsally shiny, with very fine punctation (Figs 28, 29), male antennomere 3–7 very slightly enlarged, antennomere 3 slightly more triangular than other antennomeres, median lobe longer and without such a strong modification, paramere with notch on dorsal side and subdistal part short and small, with less numerous, shorter, thick, and flattened setae (Figs 2, 3)	7
7	Beetle smaller, TL-H: 3.45–3.7 mm, MW: 1.8–2.0 mm (Fig. 28), apex of median lobe elongate in lateral view (Fig. 2D)	(24) *Exocelina waigeoensis* sp. n.
–	Beetle larger, TL-H: 3.75–4.1 mm, MW: 1.9–2.2 mm (Fig. 29), apex of median lobe truncate in lateral view (Fig. 3D)	(12) *Exocelina evelyncheesmanae* sp. n.
8	Male antennomeres 3–5 enlarged, rounded, almost equally in size and shape	9
–	Male antennomeres 3 or 3–4 distinctly more modified in shape (triangular) and larger than other antennomeres	11
9	Male antennomeres 3–5 strongly enlarged (Fig. 4A), beetle dorsally piceous (Fig. 30), male sternite 7 slightly to distinctly concave apically, median lobe with very strong median constriction and proximal part very broad in ventral view, apex of median lobe pointed and strongly curved downwards in lateral view, subdistal part of paramere with numerous, dense, long setae (Figs 4C–F)	(9) *Exocelina edeltraudae* sp. n.
–	Male antennomeres 3–5 evidently less enlarged (Figs 5A, 6A), beetle dorsally ferrugineous to dark brown, male sternite 7 slightly truncate apically, median lobe with weaker median constriction in ventral view, apex of median lobe different, subdistal part of paramere with less numerous setae	10
10	Beetle dorsally brightly ferrugineous to castaneous, submatt, with punctation coarse and dense (Fig. 31), apex of median lobe broader in ventral view, paramere with shallow notch on dorsal side (Figs 5C, E)	(13) *Exocelina hansferyi* sp. n.
–	Beetle dorsally dark brown, almost shiny, with punctation less coarse and dense (Fig. 32), apex of median lobe narrower in ventral view, paramere with distinct notch on dorsal side (Figs 6C, E)	(8) *Exocelina bundiensis* sp. n.
11	Male antennomere 3 much larger than other antennomeres, triangular (Fig. 7A), beetle larger, TL-H: 4.5–4.8 mm, MW: 2.35–2.55 mm, dark brown to blackish brown (Fig. 33), male protarsomere 4 with anterolateral hook very small (smaller than more laterally situated large seta), thin, and slightly curved, median lobe with very weak median constriction in ventral view and apex almost rounded in lateral view, paramere distinctly longer than median lobe, without notch on dorsal side, with relatively short, sparse, thin setae (Figs 7B–E)	(16) *Exocelina knoepfchen* sp. n.
–	Male antennomeres 3 and 4 much larger than other antennomeres, triangular, beetle smaller, TL-H: 3.7–4.3 mm, MW: 2.05–2.3 mm, of different color, male protarsomere 4 with anterolateral hook thin or thick, slightly curved but larger than more laterally situated large seta, median lobe with stronger median constriction in ventral view and apex different, paramere equal or shorter than median lobe, with notch on dorsal side, setae of subdistal part not numerous, relatively short, thick, and flattened	12
12	Male antennomeres 3 and 4 more strongly elongated, more equal in size and shape (Fig. 8A), elytral punctation fine, coloration dark brown to piceous (Fig. 34), apex of median lobe almost truncate in lateral view, paramere narrower (Figs 8D, E)	(1) *Exocelina alexanderi* sp. n.
–	Male antennomeres 3 and 4 less elongated, antennomere 3 larger than 4, coloration and elytral punctation different, median lobe with apex elongate in lateral view, paramere broader	13
13	Beetle dorsally ferrugineous, submatt, with coarse punctuation (Fig. 35), male protarsomere 4 with anterolateral hook thin (Fig. 9B), median lobe and paramere as in Figs 9C–E	(2) *Exocelina anggiensis* sp. n.
–	Beetle dorsally brown to piceous, shiny, with distinctly finer punctation, male protarsomere 4 with anterolateral hook thin or thick, median lobe and paramere different	14
14	Beetle dorsally piceous, with elytral punctation fine but distinct (Fig. 36), male protarsomere 4 with thick anterolateral hook (Fig. 10B), median lobe and paramere as in Figs 10C–E	(3) *Exocelina arfakensis* sp. n.
–	Beetle dorsally brown, with elytral punctation almost invisible (Fig. 37), male protarsomere 4 with thin anterolateral hook (Fig. 11B), median lobe and paramere as in Figs 11C–E	(19) *Exocelina polita*
15	Male antennomeres 3 and 4 strongly enlarged, 5 less enlarged, and 2, 6–9 slightly enlarged	16
–	Male antennomeres simple or antennomeres 3–10 slightly enlarged (stout)	17
16	Beetle reddish-brown to brown (Fig. 38), male antenna and protarsomeres 4–5 as in Figs 12A, B, apex of median lobe symmetrical in ventral view (Fig. 12C) and paramere as in Fig. 12E	(14) *Exocelina irianensis* sp. n.
–	Beetle dark brown to piceous (Fig. 39), male antenna and protarsomeres 4–5 as in Figs 13A, B, apex of median lobe asymmetrical in ventral view (Fig. 13C) and paramere as in Fig. 13E	(26) *Exocelina wondiwoiensis* sp. n.
17	Sternite 7 slightly or strongly concave apically, median lobe long, with very weak submedian constriction and narrow apex in ventral view, paramere large, with strong notch on dorsal side and subdistal part very broad, subquadrate (Figs 14C–F)	(23) *Exocelina utowaensis* sp. n.
–	Sternite 7 broadly rounded or truncate apically, median lobe distinctly shorter, paramere smaller, with weaker notch on dorsal side and subdistal part small and short or elongate (e.g. Figs 16E, 15E)	18
18	Apex of median lobe bifid: with small dorsal extension (Figs 15C, D)	(6) *Exocelina bifida* sp. n.
–	Apex of median lobe not bifid	19
19	Beetle larger, TL-H: 3.4–3.7 mm, MW: 1.75–1.95 mm (Fig. 42), apical part of median lobe very broad in ventral view and slightly flattened in lateral view, paramere with subdistal part small and short, with not numerous, relatively short, thick, and flattened setae (Figs 16C–E)	(10) *Exocelina ekari* sp. n.
–	Beetle smaller, TL-H: 3.0–3.5 mm, MW: 1.6–1.8 mm, apical part of median lobe narrower in ventral view and not flattened in lateral view, paramere with subdistal part small and short or elongate, setation different	20
20	Median lobe slender, especially its apical part, with very weak submedian constriction in ventral view, paramere with subdistal part small and short, with not numerous, relatively short, thick, flattened, slightly curved at apex setae (Figs 17C–E)	(25) *Exocelina weylandensis* sp. n.
–	Median lobe more robust, with stronger submedian constriction in ventral view, paramere with subdistal part small and short or elongate, setation different	21
21	Male protarsomere 4 with middle-sized, slender anterolateral hook (Figs 18B, 19B)	22
–	Male protarsomere 4 with large, thick, strongly curved anterolateral hook (Figs 20B, 21B, 22B, 23B)	23
22	Prosternal ridge evidently rounded and smooth, male antenna stout, median lobe with apex elongate in lateral view and submedian constriction weaker in ventral view, paramere with notch on dorsal side and subdistal part short and small, with large brush of thick, somewhat flattend, long, curved at apex setae (Figs 18A, C–E)	(21) *Exocelina soppi* sp. n.
–	Prosternal ridge anteriorly evidently less rounded and smooth, male antenna simple, median lobe with apex truncate in lateral view and submedian constriction stronger in ventral view, paramere with shallow notch on dorsal side and subdistal part elongate, with strong tuft of thicker, somewhat flattend, and strongly curved at apex setae (Figs 19A, C–E)	(20) *Exocelina pseudosoppi* sp. n.
23	Subdistal part of paramere with two kinds of setae: thin upper setae and thick and flattened lower setae, proximal part of paramere with sparse setae (Figs 20E, 21E)	24
–	Subdistal part of paramere only with thin setae, proximal part of paramere with dense setae (Figs 22E, 23E)	25
24	Male antennomeres 3–10 stout, median lobe shorter, its apex broader in ventral view and slightly elongate in lateral view, subdistal part of paramere with upper setae less numerous and lower setae long, thick, flattend, and curved at apex (Figs 20A, C–E)	(11) *Exocelina eme* sp. n.
–	Male antennomeres simple, median lobe longer, its apex narrower in ventral view and almost truncate in lateral view, subdistal part of paramere with upper setae more numerous and lower setae shorter, thicker, and flattend (Figs 21A, C–E)	(7) *Exocelina brahminensis* sp. n.
25	Male antennomeres simple, median lobe with apex almost truncate in lateral view and submedian constriction stronger in ventral view, paramere with setae of proximal part longer, thicker, distinctly visible (Figs 22A, C–E)	(15) *Exocelina kakapupu* sp. n.
–	Male antennomeres 3–10 stout, median lobe with apex elongate in lateral view and submedian constriction weaker in ventral view, paramere with setae of proximal part shorter, thiner, often hardly visible (Figs 23A, C–E)	(22) *Exocelina unipo* sp. n.

**Figure 6.**
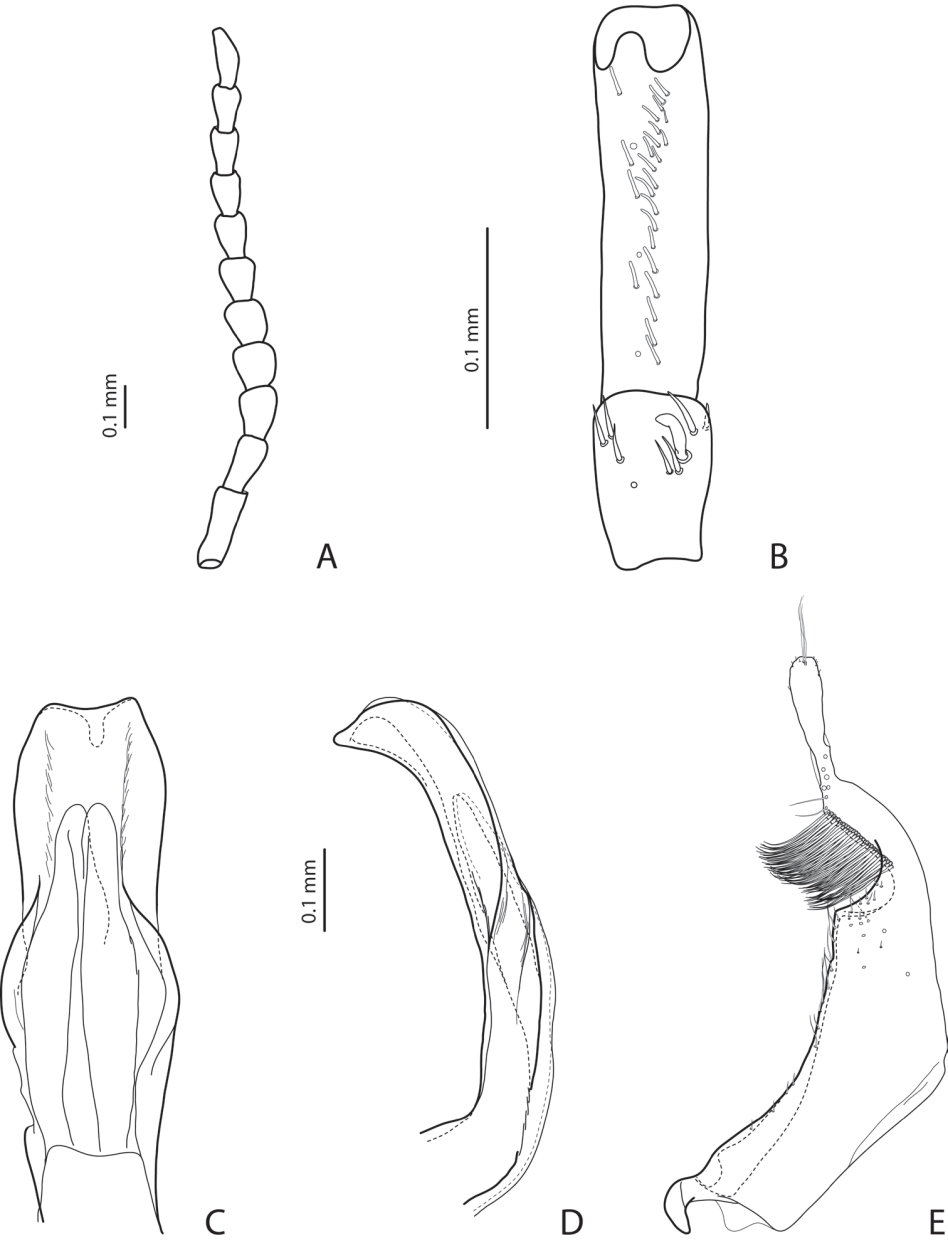
*Exocelina bundiensis* sp. n. **A** male antenna **B** protarsomeres 4–5 in ventral view **C** median lobe in ventral view **D** median lobe in lateral view **E** paramere in external view.

**Figure 7.**
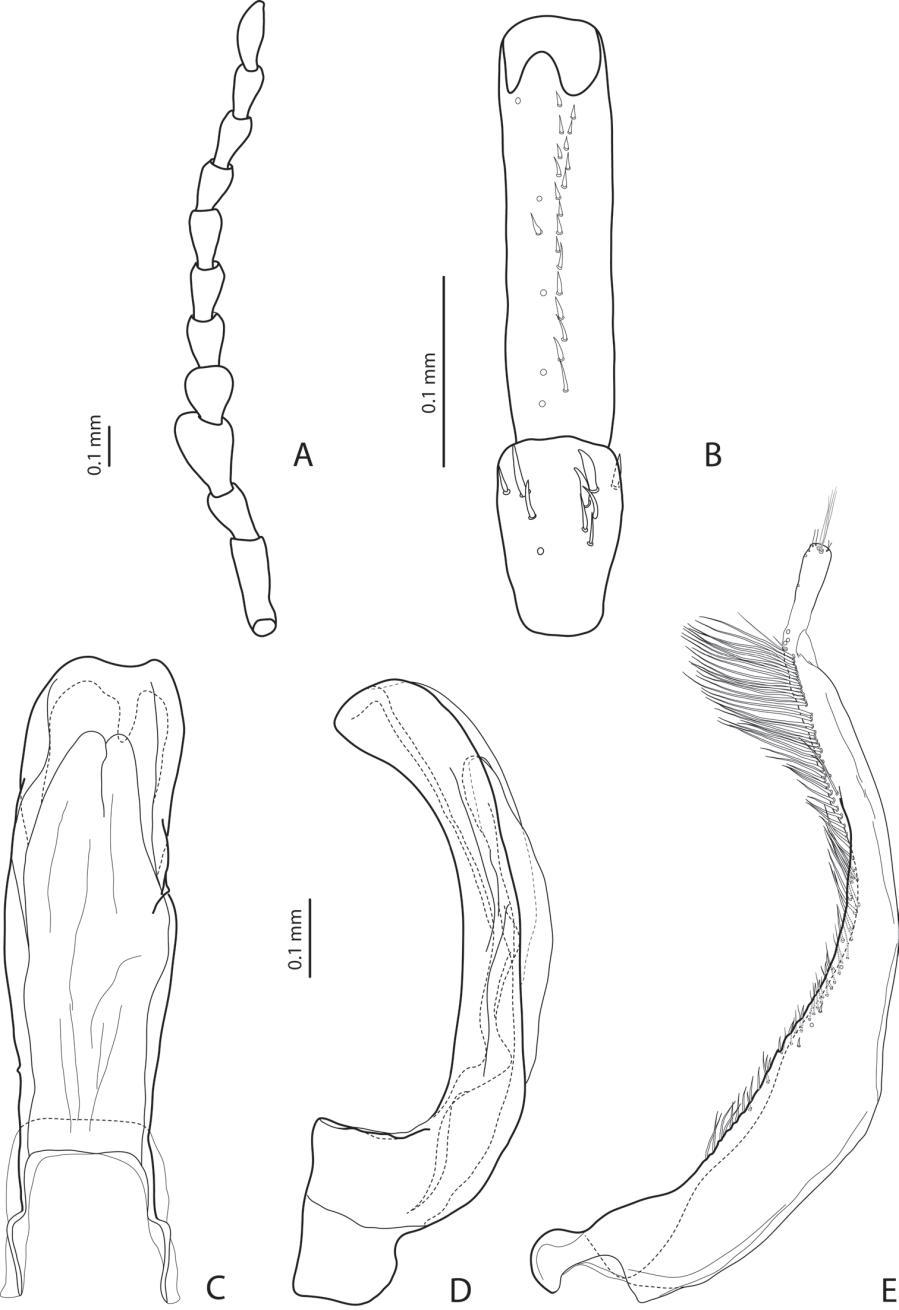
*Exocelina knoepfchen* sp. n. **A** male antenna **B** protarsomeres 4–5 in ventral view **C** median lobe in ventral view **D** median lobe in lateral view **E** paramere in external view.

**Figure 8.**
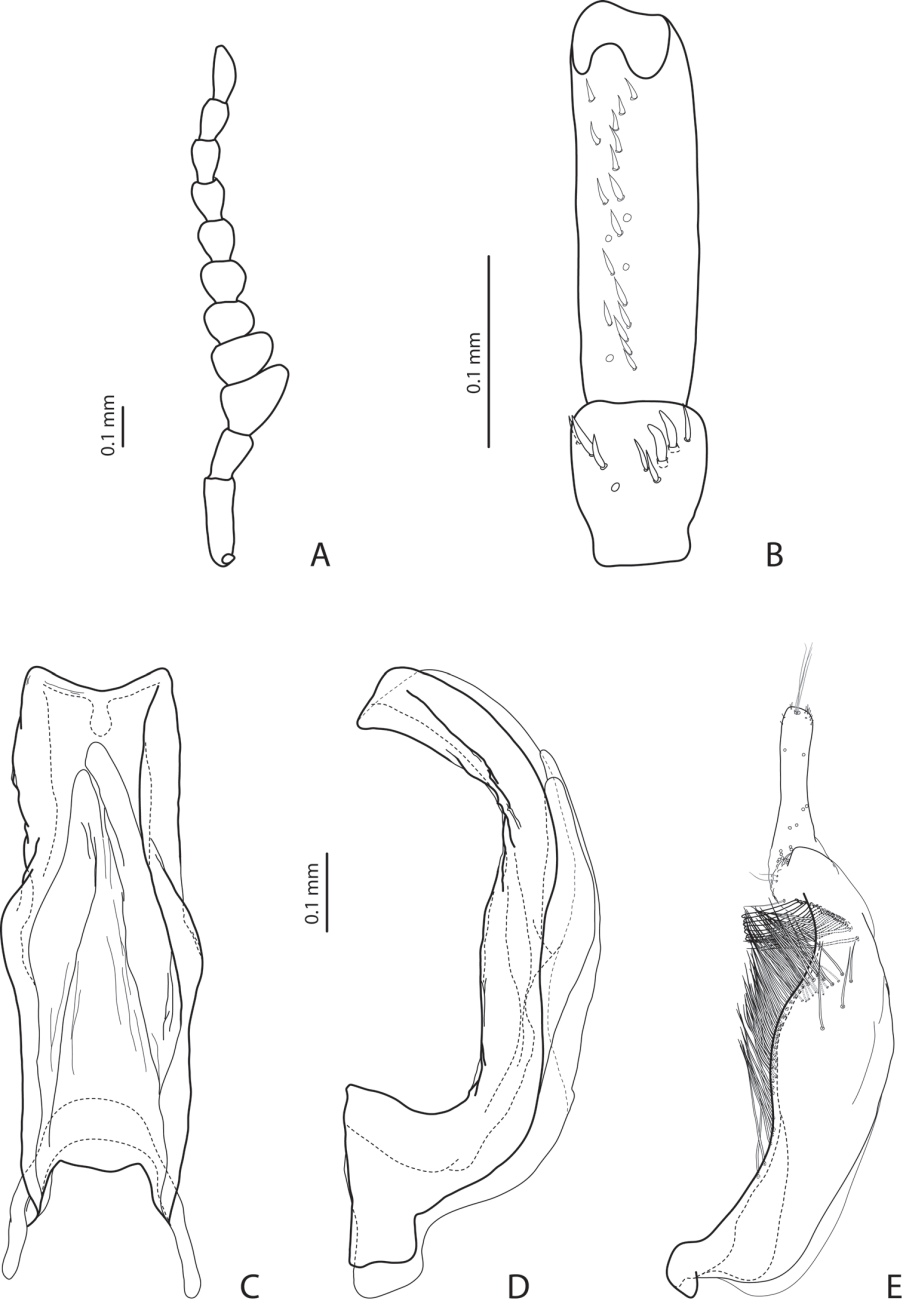
*Exocelina alexanderi* sp. n. **A** male antenna **B** protarsomeres 4–5 in ventral view **C** median lobe in ventral view **D** median lobe in lateral view **E** paramere in external view.

**Figure 9.**
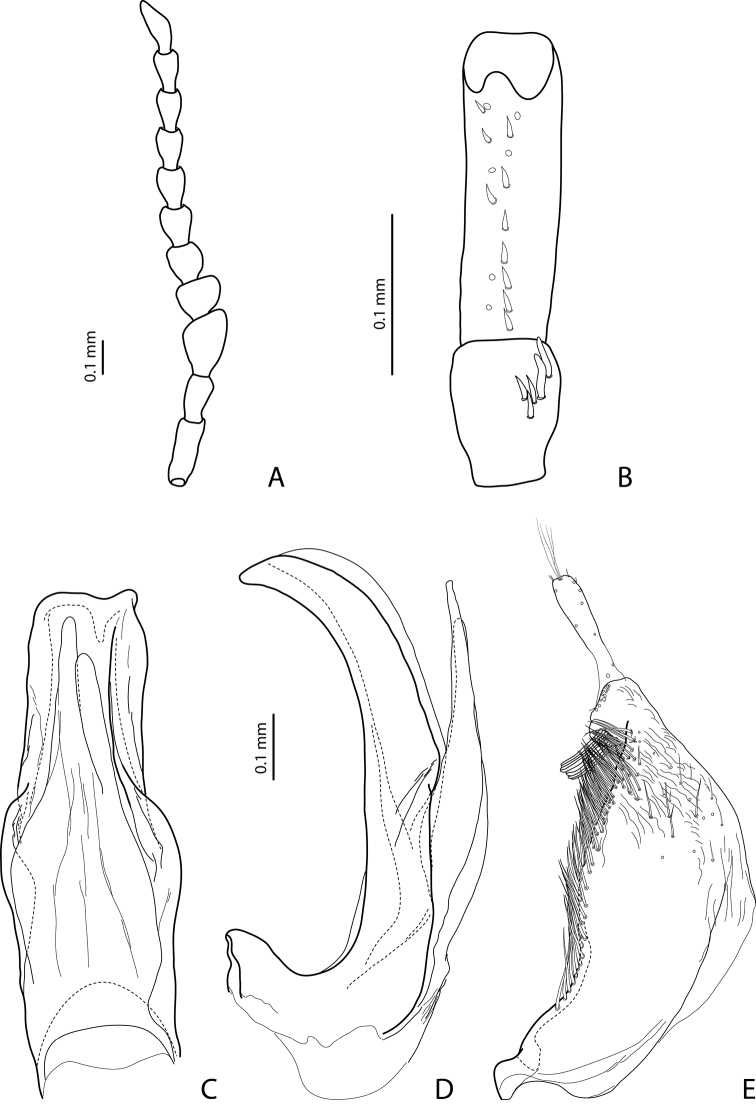
*Exocelina anggiensis* sp. n. **A** male antenna **B** protarsomeres 4–5 in ventral view **C** median lobe in ventral view **D** median lobe in lateral view **E** paramere in external view.

**Figure 10.**
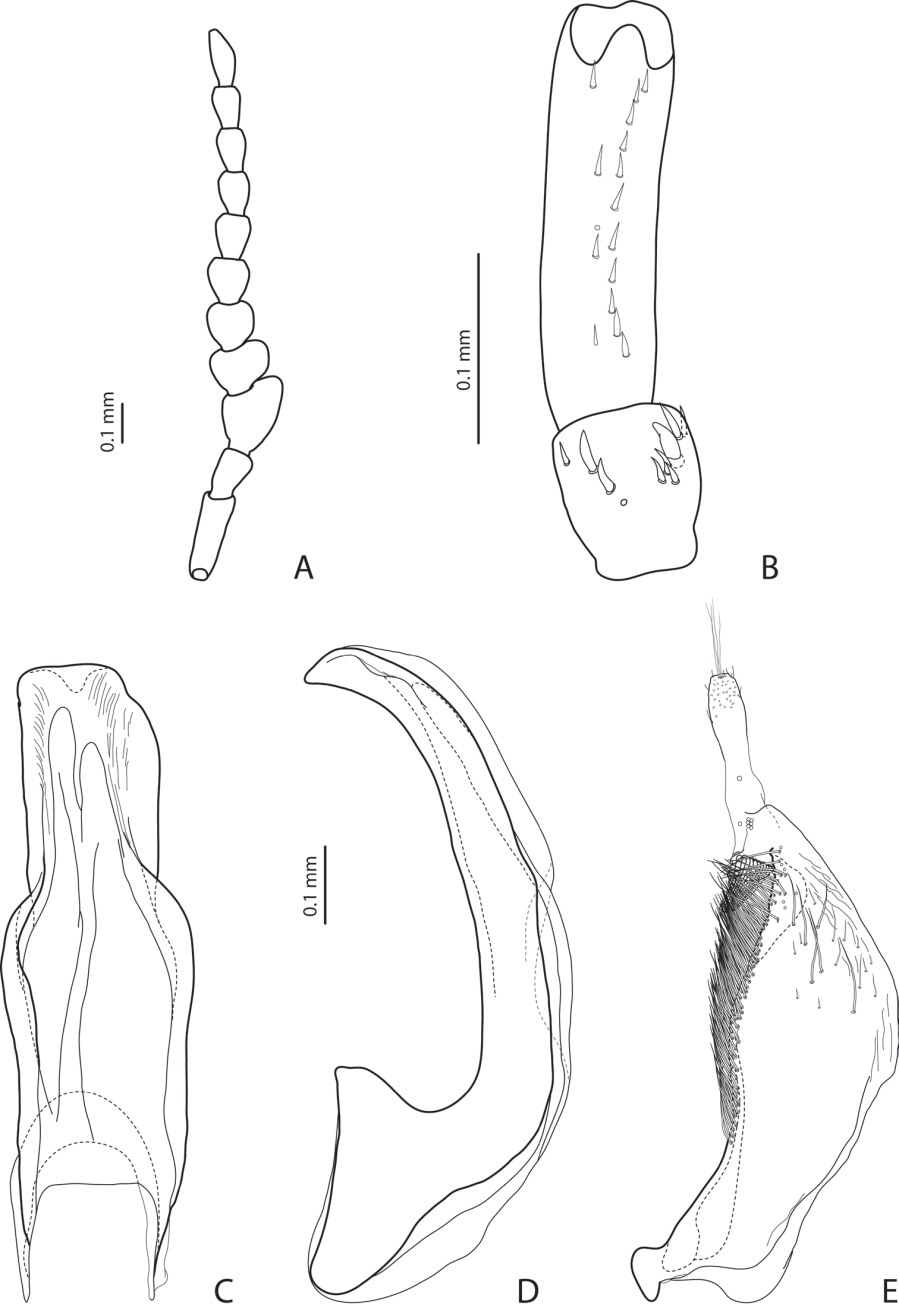
*Exocelina arfakensis* sp. n. **A** male antenna **B** protarsomeres 4–5 in ventral view **C** median lobe in ventral view **D** median lobe in lateral view **E** paramere in external view.

**Figure 11.**
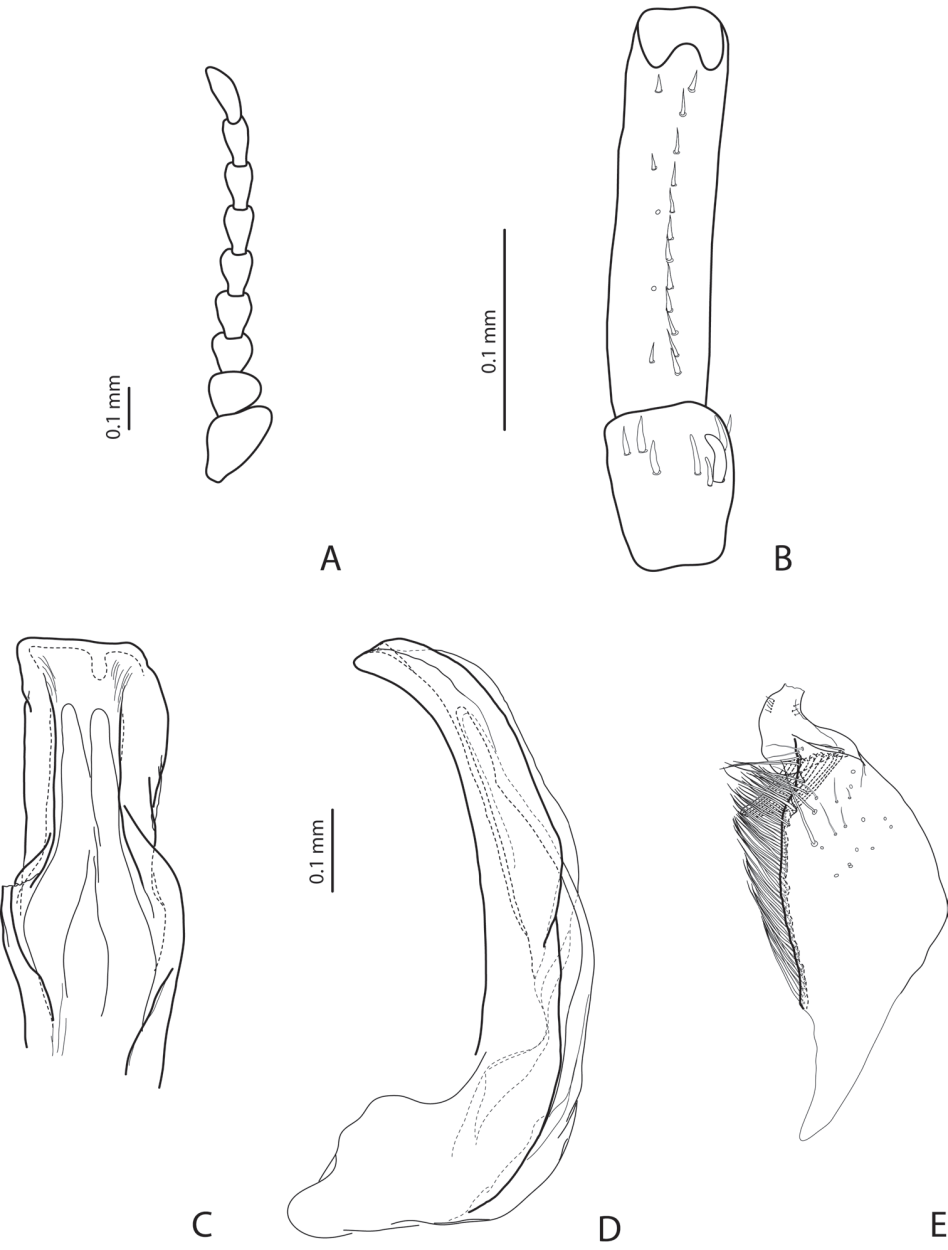
*Exocelina polita* (Sharp, 1882) **A** male antenna (without antennomeres 1, 2) **B** protarsomeres 4–5 in ventral view **C** median lobe in ventral view **D** median lobe in lateral view **E** paramere in external view.

**Figures 12–13.**
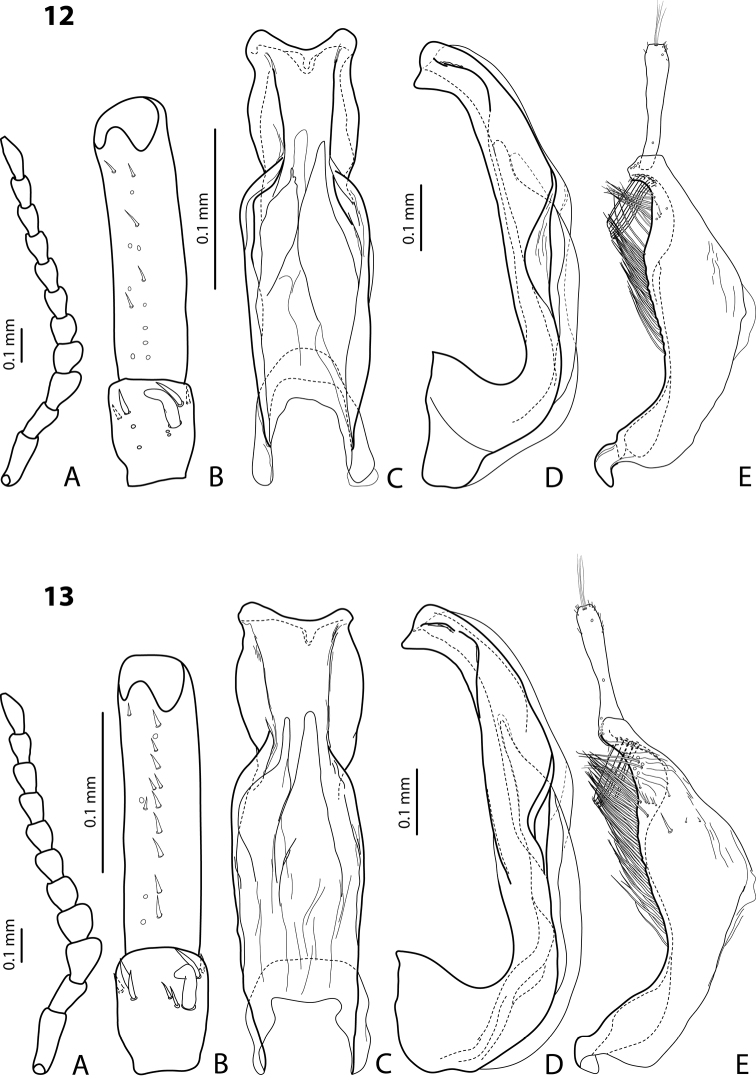
**12** *Exocelina irianensis* sp. n. **13** *Exocelina wondiwoiensis* sp. n. **A** male antenna **B** protarsomeres 4–5 in ventral view **C** median lobe in ventral view **D** median lobe in lateral view **E** paramere in external view.

**Figure 14.**
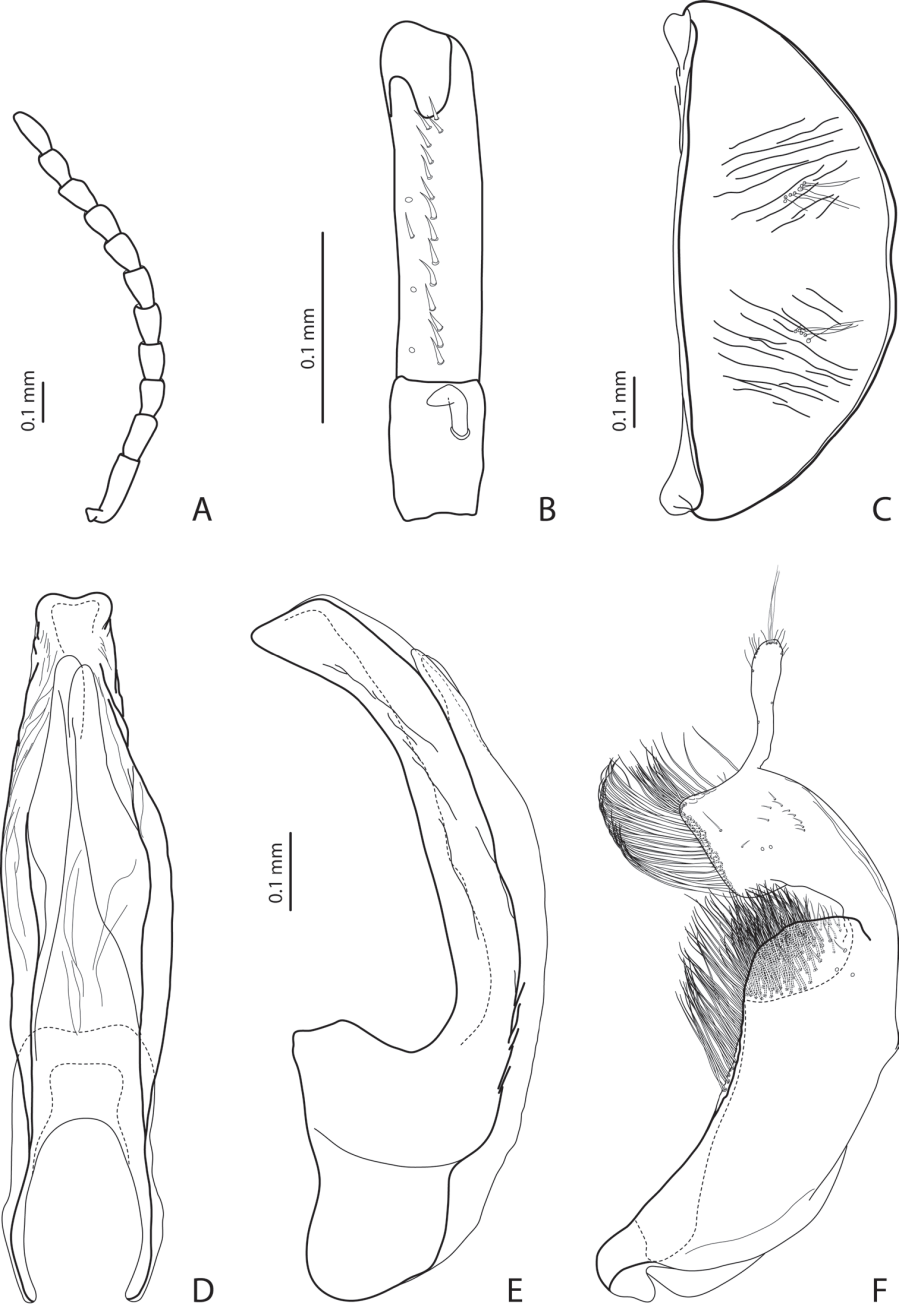
*Exocelina utowaensis* sp. n. **A** male antenna **B** protarsomeres 4–5 in ventral view **C** abdominal sternite 7 **D** median lobe in ventral view **E** median lobe in lateral view **F** paramere in external view.

**Figure 15.**
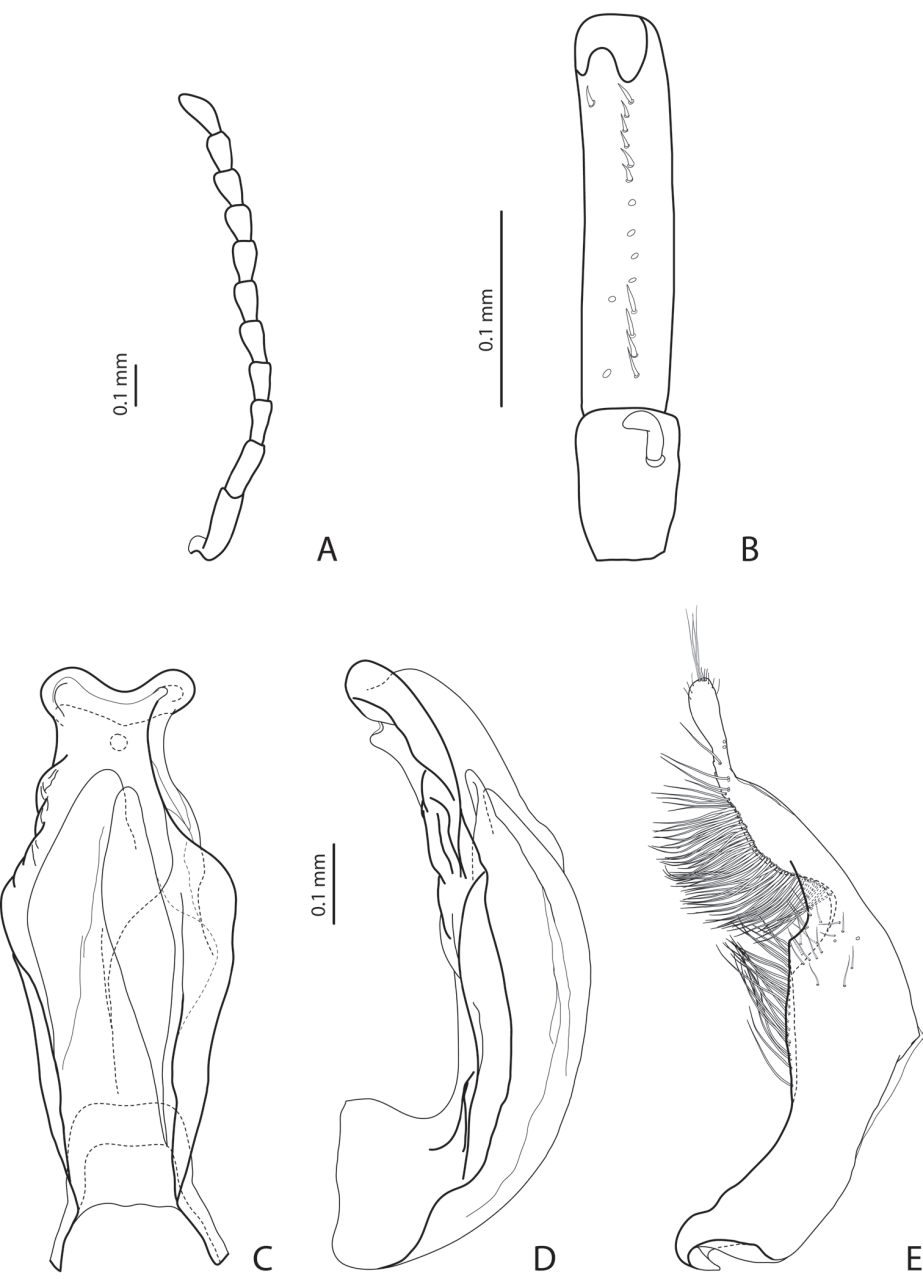
*Exocelina bifida* sp. n. **A** male antenna **B** protarsomeres 4–5 in ventral view **C** median lobe in ventral view **D** median lobe in lateral view **E** paramere in external view.

**Figures 16–17.**
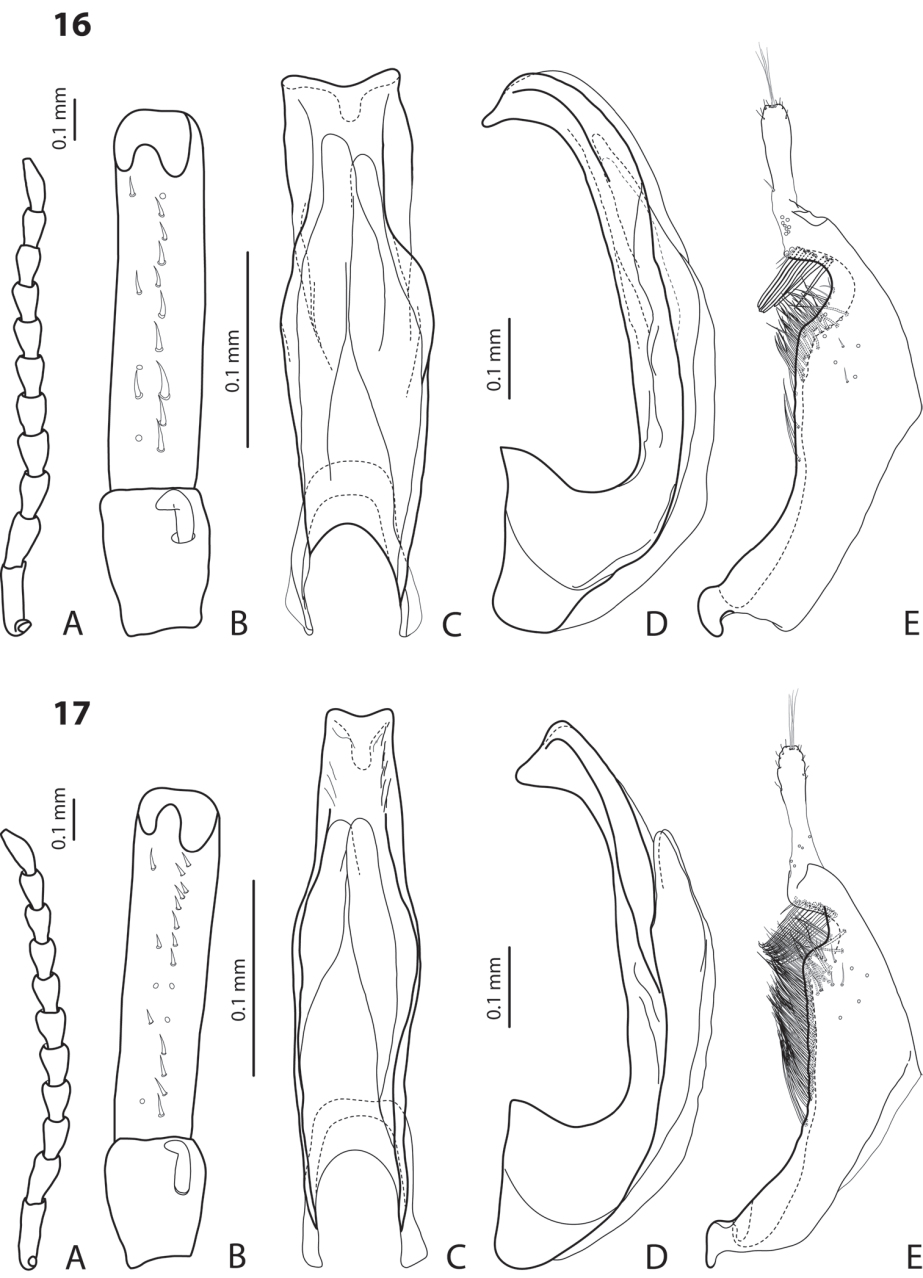
**16** *Exocelina ekari* sp. n. **17** *Exocelina weylandensis* sp. n. **A** male antenna **B** protarsomeres 4–5 in ventral view **C** median lobe in ventral view **D** median lobe in lateral view **E** paramere in external view.

**Figures 18–19.**
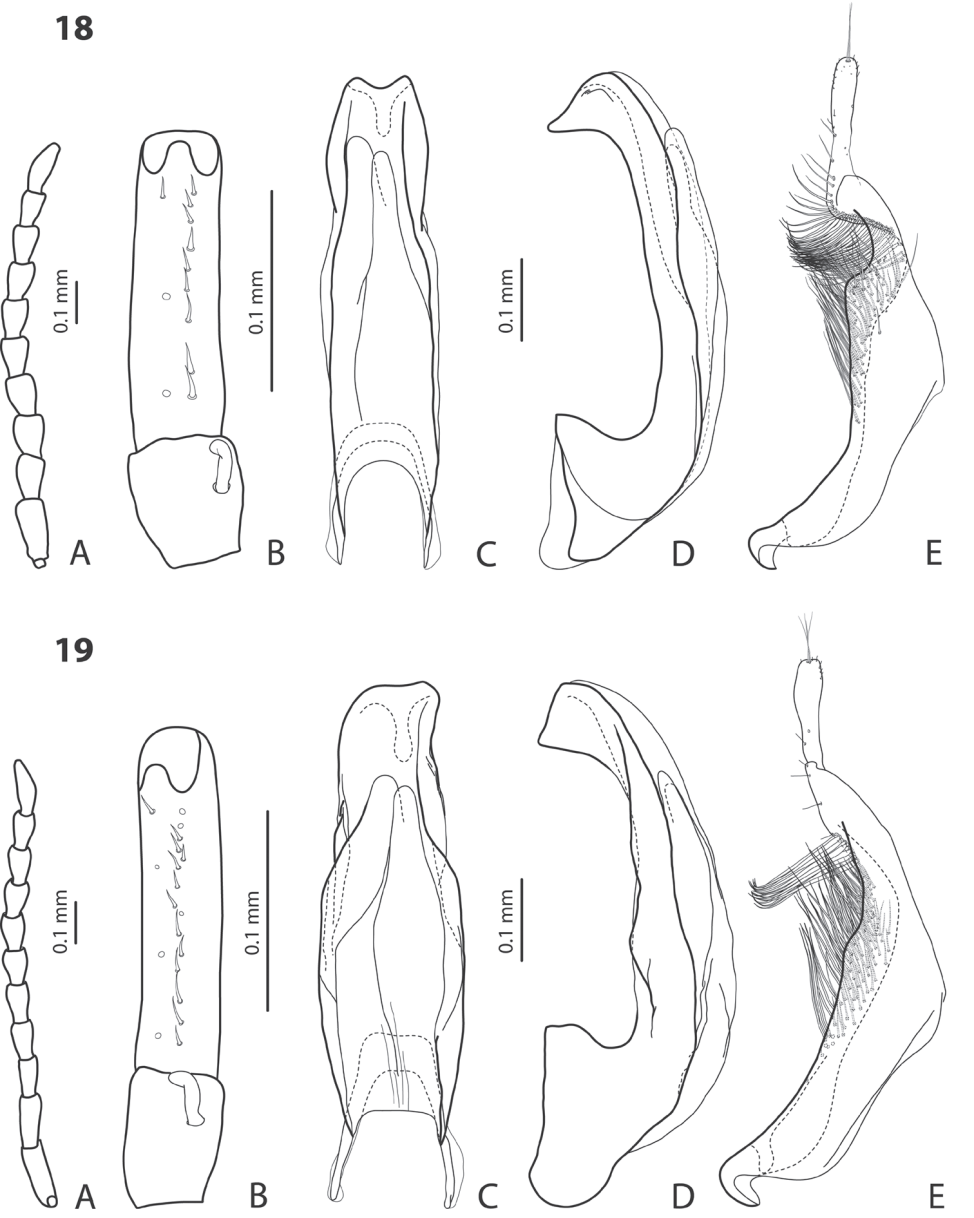
**18** *Exocelina soppi* sp. n. **19** *Exocelina pseudosoppi* sp. n. **A** male antenna **B** protarsomeres 4–5 in ventral view **C** median lobe in ventral view **D** median lobe in lateral view **E** paramere in external view.

**Figures 20–21.**
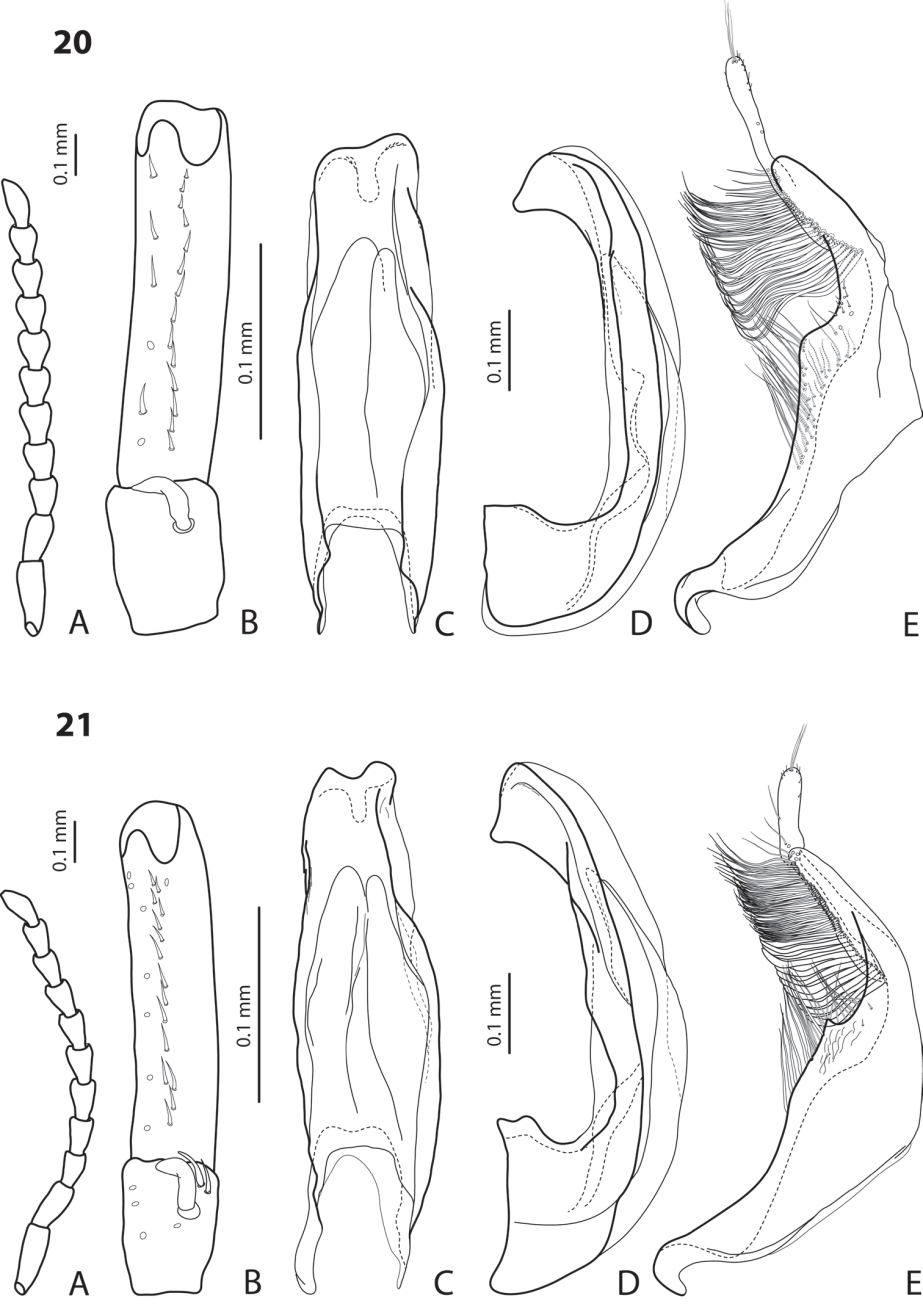
**20** *Exocelina eme* sp. n. **21** *Exocelina brahminensis* sp. n. **A** male antenna **B** protarsomeres 4–5 in ventral view **C** median lobe in ventral view **D** median lobe in lateral view **E** paramere in external view.

**Figures 22–23.**
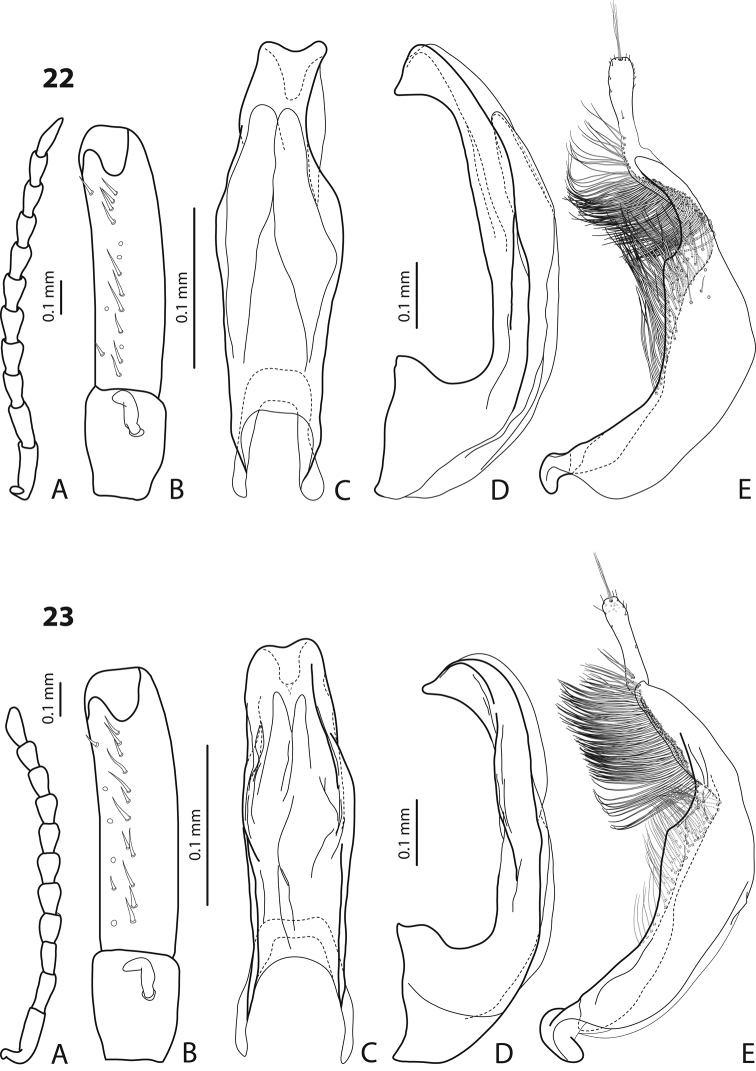
**22** *Exocelina kakapupu* sp. n. **23** *Exocelina unipo* sp. n. **A** male antenna **B** protarsomeres 4–5 in ventral view **C** median lobe in ventral view **D** median lobe in lateral view **E** paramere in external view.

**Figures 24–27.**
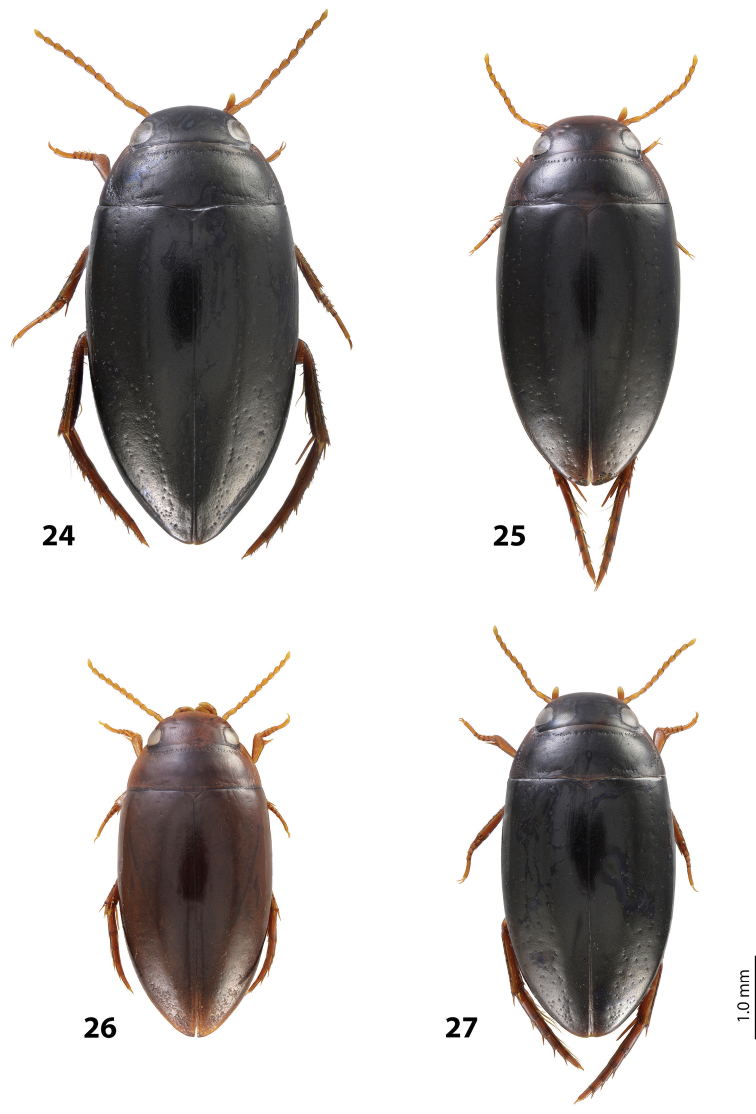
Habitus and coloration. **24** *Exocelina munaso* (Shaverdo, Sagata & Balke, 2005) **25** *Exocelina atowaso* (Shaverdo, Sagata & Balke, 2005) **26** *Exocelina oceai* sp. n. **27** *Exocelina astrophallus* (Balke, 1998).

**Figures 28–31.**
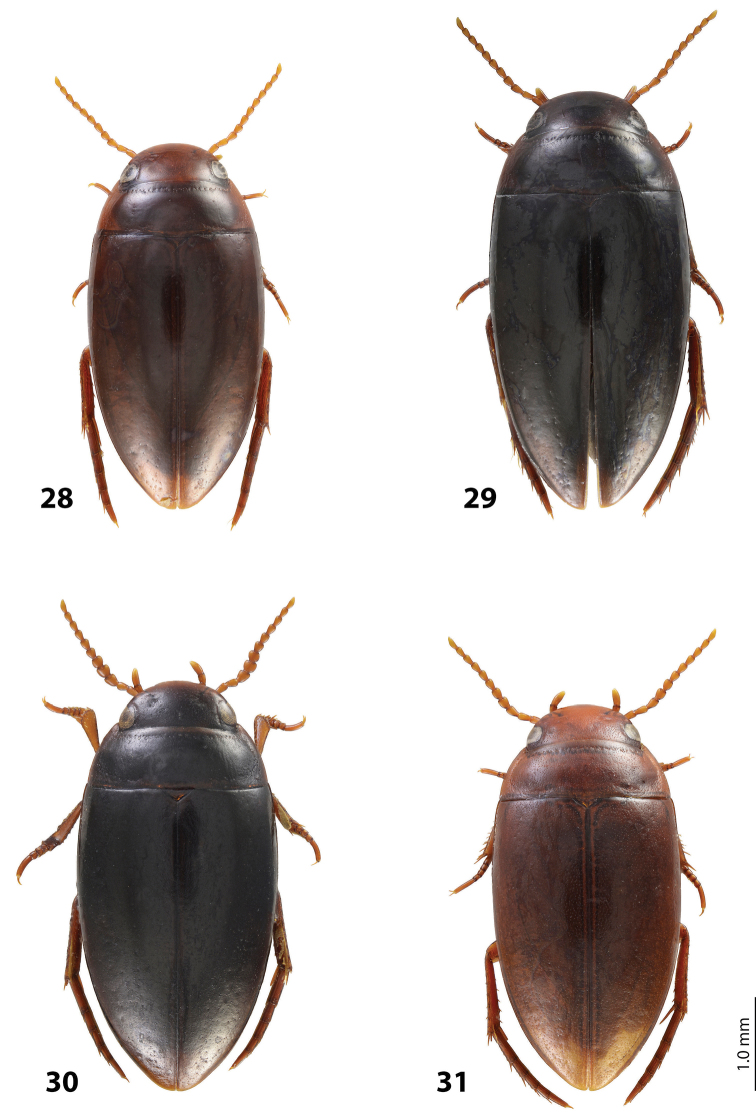
Habitus and coloration. **28** *Exocelina waigeoensis* sp. n. **29** *Exocelina evelyncheesmanae* sp. n. **30** *Exocelina edeltraudae* sp. n. **31** *Exocelina hansferyi* sp. n.

**Figures 32–35.**
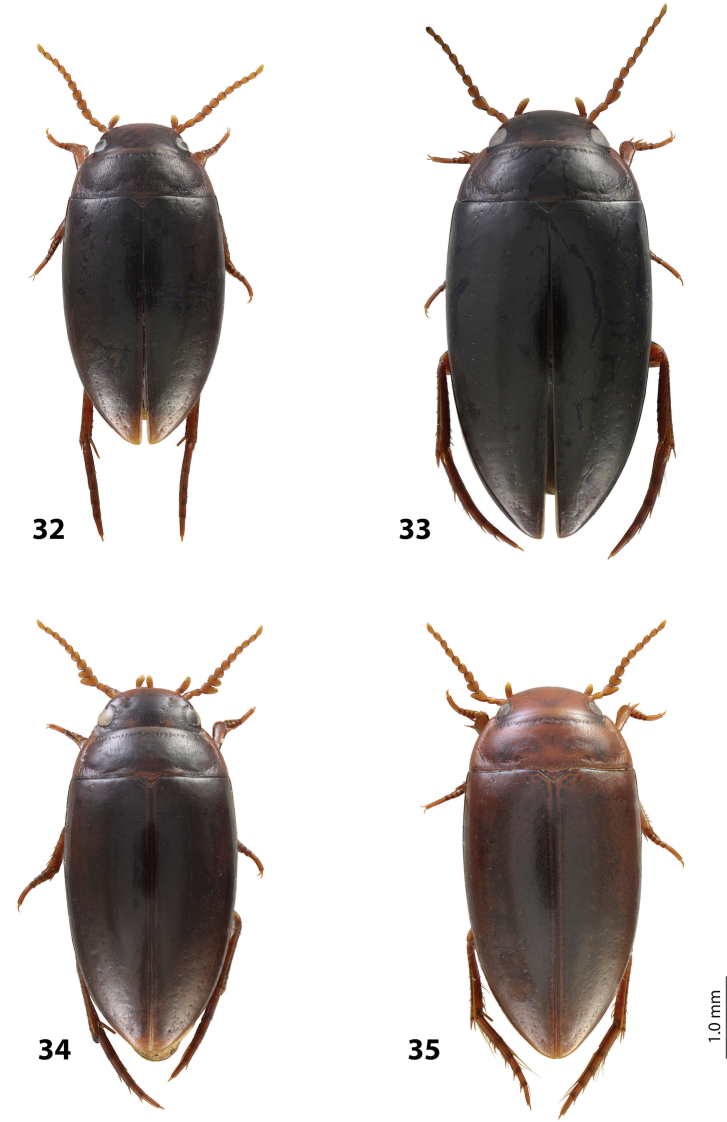
Habitus and coloration. **32** *Exocelina bundiensis* sp. n. **33** *Exocelina knoepfchen* sp. n. **34** *Exocelina alexanderi* sp. n. **35** *Exocelina anggiensis* sp. n.

**Figures 36–39.**
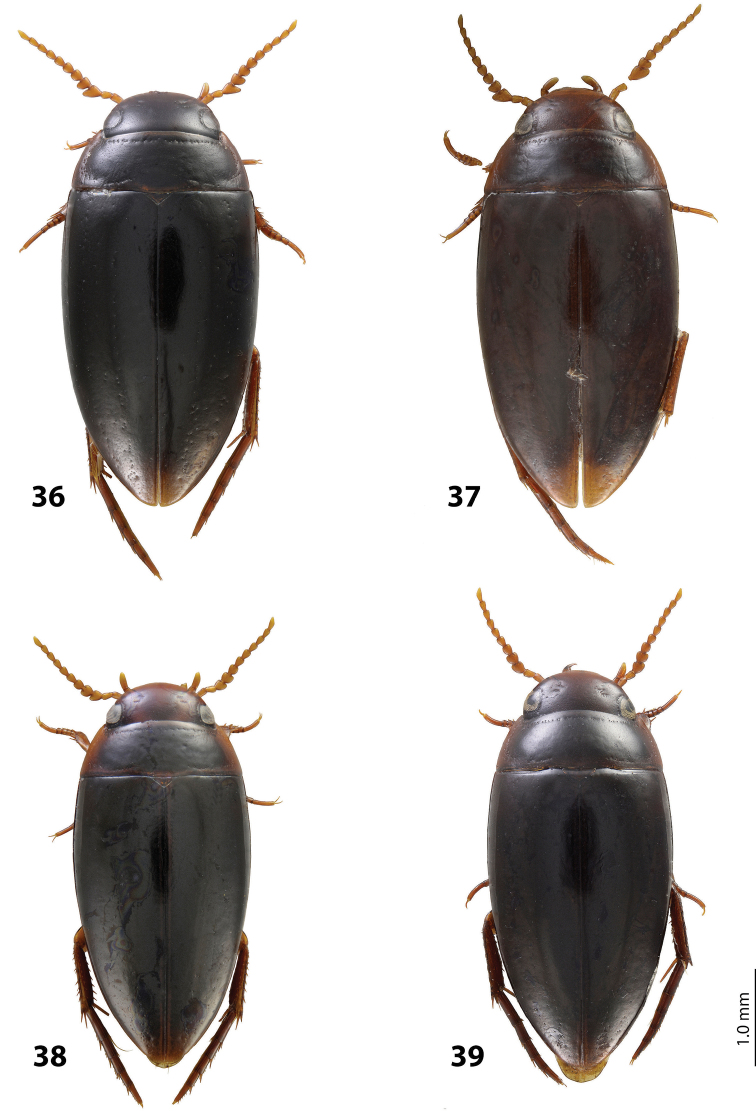
Habitus and coloration.**36** *Exocelina arfakensis* sp. n. **37** *Exocelina polita* (Sharp, 1882) **38** *Exocelina irianensis* sp. n. **39** *Exocelina wondiwoiensis* sp. n.

**Figures 40–43.**
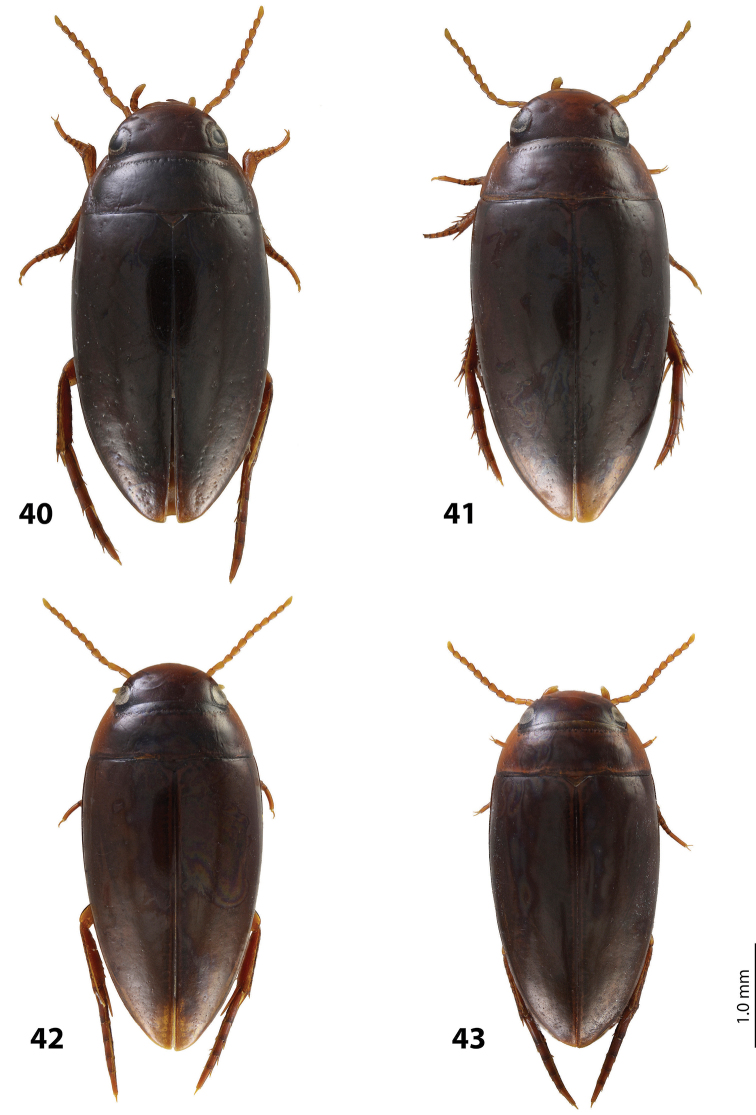
Habitus and coloration. **40** *Exocelina utowaensis* sp. n. **41** *Exocelina bifida* sp. n. **42** *Exocelina ekari* sp. n. **43** *Exocelina weylandensis* sp. n.

**Figures 44–47.**
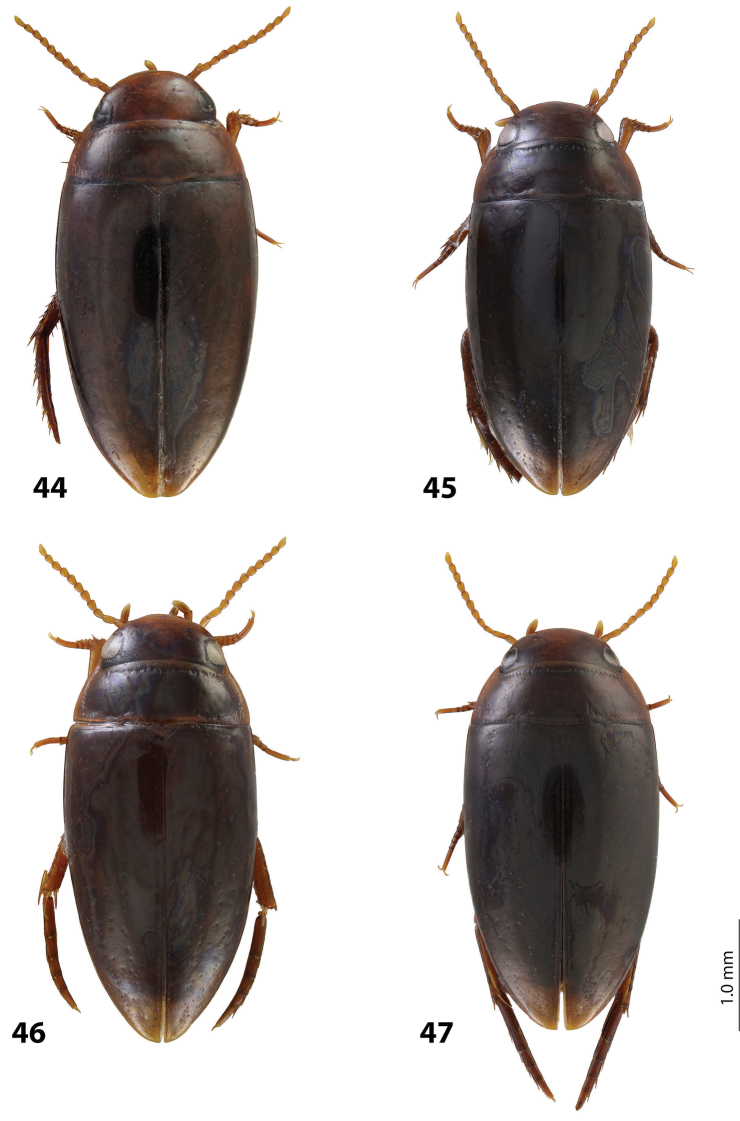
Habitus and coloration. **44** *Exocelina soppi* sp. n. **45** *Exocelina pseudosoppi* sp. n. **46** *Exocelina eme* sp. n. **47** *Exocelina brahminensis* sp. n.

**Figures 48–49.**
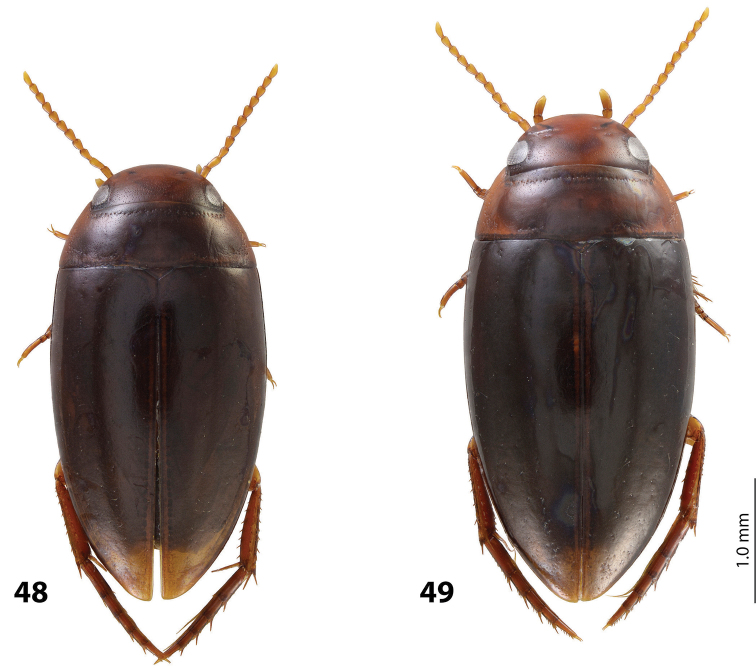
Habitus and coloration. **48** *Exocelina kakapupu* sp. n. **49** *Exocelina unipo* sp. n.

**Figure 50.**
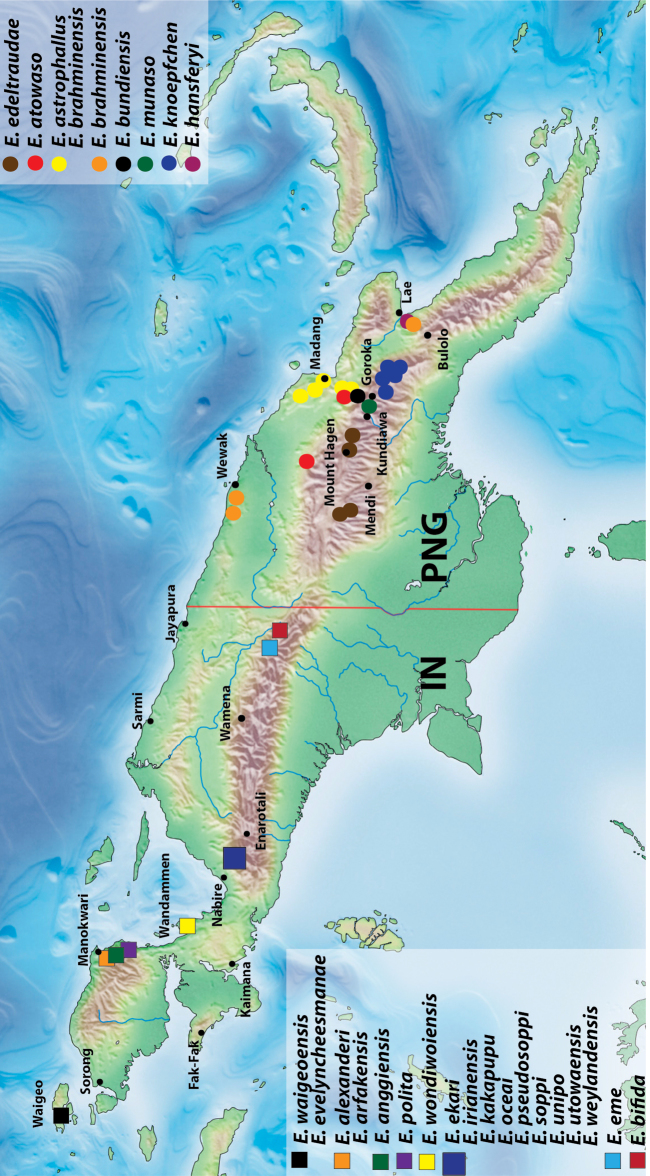
Map of New Guinea showing distribution of the species of the *Exocelina ekari*-group.

## Habitats

The species are always running water associated, but they do avoid water movement. This appears paradox, but it is a very important microhabitat preference especially in many tropical ecosystems. New Guinean *Exocelina* species are found in streambeds, usually the smaller the better, at the edge where there are small backflows. There, the beetles are only found where there is no current at all. Small isolated water holes, usually on heavy red clay, are another preferred habitat. The beetles may be found on wet rocks along streambeds, hiding under leaves, in rock pools, or tiny water filled holes in rocks, along the streams, as well as in rest pools of intermittent streams, in wet gravel, underneath of rocks in dry streambeds, and in tiny, shallow water holes on slopes above the actual spring of a stream. It is worth to note that it is often the best approach to climb a ridge up to a suitable point and then descend a steep slope until the first temporary water holes occur. This yields often large series of specimens – such as on Waigeo Island, on Mountain Nok, where *Exocelina* beetles were extremely common in such habitats: the first stagnant water availabel underneath the summit, often where roots and rocks contained water and formed small puddles on the clay. Few meters below, where the first order stream was already running slowly, we did hardly encounter specimens. In limestone areas, such as on Batanta Island and in Fak Fak (species treated in a future paper), we followed a stream up to a point where the entire, 10 m wide, streambed was dry. Specimens were found in small water holes on large limestone boulders. Sometimes, *Exocelina* species occur on sandy/gravelly river banks, where they can either be seen swimming around in small pools, or are collected by removing rocks from the wet ground where a small puddle will form in the imprint, and specimens can then be washed out of the gravel by hand and with a strainer. At the edge of large (montane) rivers, we found large numbers of specimens in pools along the river.

In general, clay, mossy rocks, as well as presence of rough sand/gravel indicate rich *Exocelina* fauna, as this allows the beetles to hide well and dig into the ground to avoid flooding of streams. Shaded locations are preferred – often, the only shaded spot along a stretch of river, otherwise fully exposed, contains all *Exocelina* beetles that can be found.

**Figure 51.**
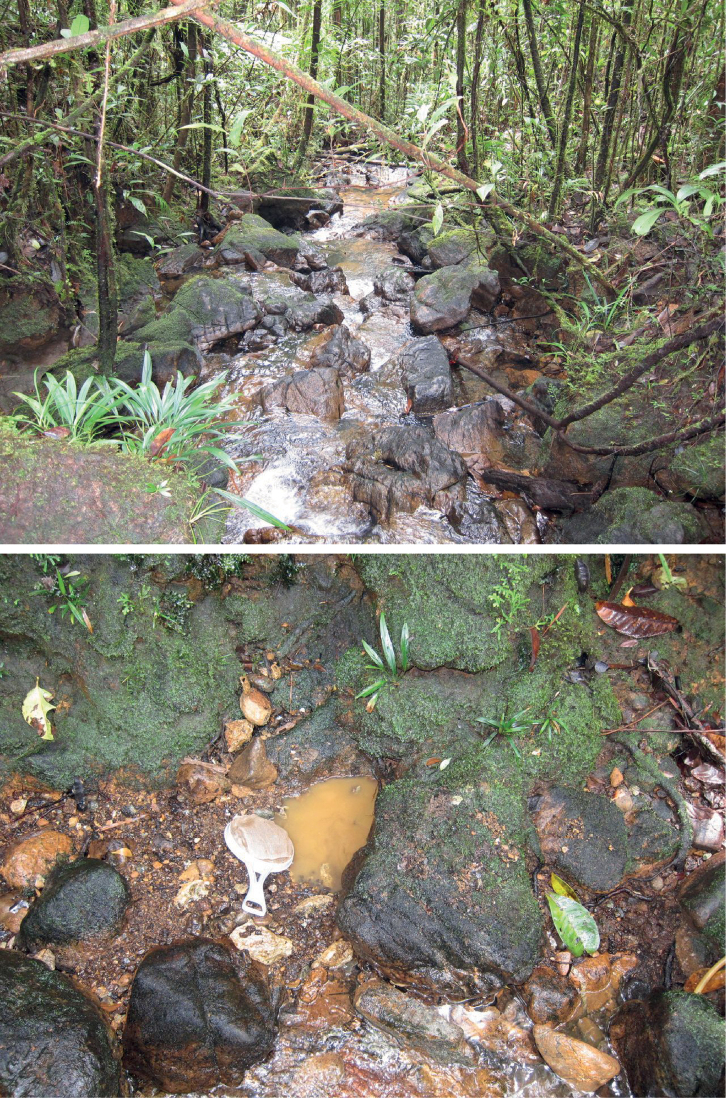
Habitat of *Exocelina irianensis* sp. n.: Indonesia, Papua Province, Nabire-Enarotali Road, 55^th^ km, stream on the steep slope of the montane forest with the heavy red clay, mossy boulders, and abundant rough sand/gravel that being characteristic for this area (Mount Gamey).

**Figure 52.**
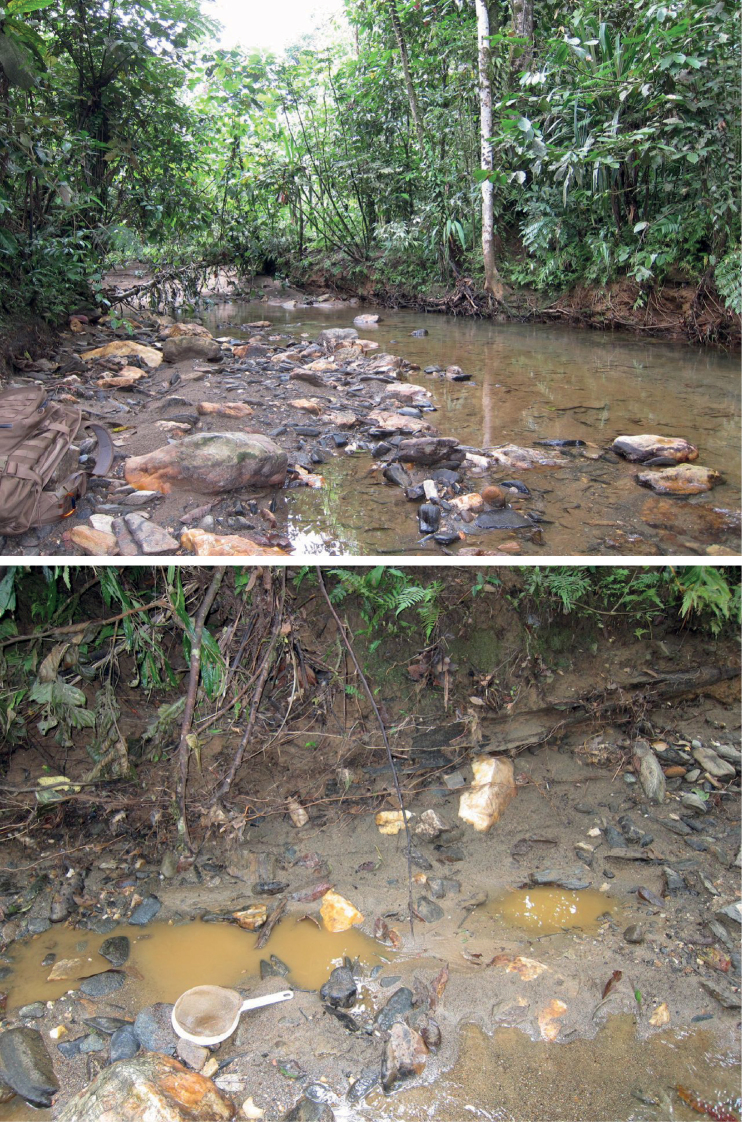
Habitat of *Exocelina utowaensis* sp. n. and *Exocelina pseudosoppi* sp. n.: Indonesia, Papua Province, Nabire-Enarotali Road, 62^nd^ km, stream in the flat stretch of the lowland forest.

**Figures 53.**
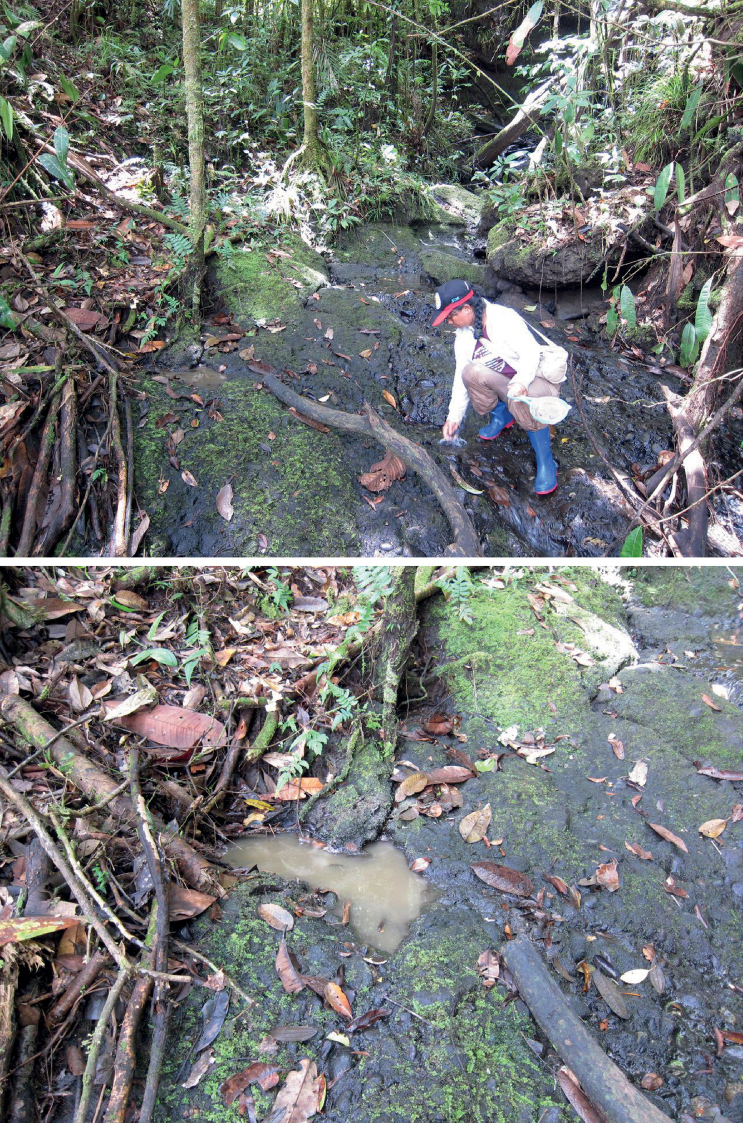
Habitat of *Exocelina unipo* sp. n.: Indonesia, Papua Province, Nabire-Enarotali Road, 108^th^ km, stream on the steep slope (Suriani Surbakti).

Figures 51–53 illustrate some typical habitats and their microhabitats, but there are countless variations of the general theme. Fast flowing streams on steep slopes such as in Figs 51, 53 are notoriously hard to sample, as this requires hiking up the stream in the water, on very slippery ground and cutting through vegetation across the streambed in order to find suitable microhabitats. In situations like that, it might be advisable also to travel around and try to find possibly dry lower order streams, which might contain the same species. Riverbank habitats (Fig. 52) are, on the other hand, much easier to access and to sample, and it is helpful to utilize strong gloves to dig through the gravel, removing stone by stone, and excavate gravel (which is often very sharp) in order to crate small puddles which will eventually reveal *Exocelina* beetles out of the interstitial. For more information see http://zsm-entomology.de/wiki/Coleoptera_Fieldwork .

## Supplementary Material

XML Treatment for Exocelina alexanderi

XML Treatment for Exocelina anggiensis

XML Treatment for Exocelina arfakensis

XML Treatment for Exocelina astrophallus

XML Treatment for Exocelina atowaso

XML Treatment for Exocelina bifida

XML Treatment for Exocelina brahminensis

XML Treatment for Exocelina bundiensis

XML Treatment for Exocelina edeltraudae

XML Treatment for Exocelina ekari

XML Treatment for Exocelina eme

XML Treatment for Exocelina evelyncheesmanae

XML Treatment for Exocelina hansferyi

XML Treatment for Exocelina irianensis

XML Treatment for Exocelina kakapupu

XML Treatment for Exocelina knoepfchen

XML Treatment for Exocelina munaso

XML Treatment for Exocelina oceai

XML Treatment for Exocelina polita

XML Treatment for Exocelina pseudosoppi

XML Treatment for Exocelina soppi

XML Treatment for Exocelina unipo

XML Treatment for Exocelina utowaensis

XML Treatment for Exocelina waigeoensis

XML Treatment for Exocelina weylandensis

XML Treatment for Exocelina wondiwoiensis
